# Advancing dynamic quantum crystallography: enhanced models for accurate structures and thermodynamic properties

**DOI:** 10.1107/S2052252524011862

**Published:** 2025-01-01

**Authors:** Helena Butkiewicz, Michał Chodkiewicz, Anders Ø. Madsen, Anna A. Hoser

**Affiliations:** a Faculty of Chemistry University of Warsaw Pasteura 1 Warsaw 02-093 Poland; b Department of Pharmacy University of Copenhagen Copenhagen Denmark; Universidad de Oviedo, Spain

**Keywords:** anisotropic displacement parameters, ADPs, entropy, lattice dynamics, aspherical atom model, com­putational modelling, density functional theory, polymorphism

## Abstract

X-ray diffraction (XRD) has evolved significantly since its inception, becoming a crucial tool for material structure characterization. Advancements in theory, experimental techniques, diffractometers and detection technology have led to the acquisition of highly accurate diffraction patterns, surpassing previous expectations. Extracting com­prehensive information from these patterns necessitates different models due to the influence of both electron density and thermal motion on diffracted beam intensity. While electron-density modelling has seen con­siderable progress [*e.g.* the Hansen–Coppens multipole model and Hirshfeld Atom Refinement (HAR)], the treatment of thermal motion has remained largely unchanged. We have developed a novel method that combines the strengths of the advanced charge-density models [Aspherical Atom Models (AAMs), such as HAR or the Transferable Aspherical Atom Model (TAAM)] and the thermal motion model (normal modes refinement, NoMoRe). We denote this approach AAM_NoMoRe, wherein instead of refining routine anisotropic displacement parameters (ADPs) against single-crystal X-ray diffraction data, we refine the frequencies obtained from periodic density functional theory (DFT) calculations. In this work, we demonstrate the effectiveness of this model by presenting its application to model com­pounds, such as alanine, xylitol, naphthalene and glycine polymorphs, highlighting the influence of our method on the H-atom positions and shape of their ADPs, which are com­parable with neutron data. We observe a significant decrease in the similarity index for H-atom ADPs after AAM_NoMoRe in com­parison to only AAM, aligning more closely with neutron data. Due to the use of aspherical form factors (AAM), our approach demonstrates better fitting performance, as indicated by consistently lower *wR*2 values com­pared to the Independent Atom Model (IAM) refinement and a significant decrease com­pared to the traditional NoMoRe model. Furthermore, we present the estimation of a key thermodynamic property, namely, heat capacity, and demonstrate its alignment with experimental calorimetric data.

## Abstract 2

By implementing the aspherical atom model to normal mode refinement, we obtained accurate structures [including H-atom positions and anisotropic displacement parameters (ADPs)] and heat capacity from single-crystal X-ray diffraction data.

## 1. Introduction

The stability of a crystal structure is determined by its Gibbs free energy, which encom­passes not only the lattice energy calculated for the static structure but also the enthalpic and entropic contributions originating from thermal vibrations and from disorder. Single-crystal X-ray diffraction experiments provide valuable insights into both atomic coordinates and thermal vibrations. While atomic coordinates are often utilized in lattice-energy calculations, the information regarding thermal motion is often neglected. It has been demonstrated that, when a structure does not exhibit signs of disorder, it is possible to extract low-frequency modes using data from single-crystal X-ray diffraction measurements. Consequently, this enables the estimation of vibrational entropy and other thermodynamic properties. This field’s pioneer was Cruickshank, who, in 1956, estimated the entropy of crystalline naphthalene (Cruickshank, 1956 ▸). Building upon Cruickshank’s work, Schomaker and Trueblood introduced the translation–libration–screw (**TLS**) analysis (Schomaker & Trueblood, 1968 ▸), which allows the estimation of vibrational entropy based on frequencies obtained for translational and librational modes (Madsen & Larsen, 2007 ▸; Madsen *et al.*, 2011 ▸; Jarzembska *et al.*, 2014 ▸). Moreover, Bürgi and Cappelli extended the **TLS** model and developed an elegant approach known as the normal mode coordinate analysis (NKA) (Bürgi & Capelli, 2000 ▸). Some time ago, Aree, Bürgi and co-workers undertook a com­prehensive examination of multi-tem­per­a­ture data sets for three polymorphs of glycine in a series of articles (Aree *et al.*, 2012 ▸, 2013 ▸, 2014 ▸). Using synchrotron radiation data, they applied different charge-density models to extract optimal ADPs and employed the NKA model to analyse the thermodynamics of all solid forms. Users of NKA are required to make decisions regarding the frequencies for which modes should be obtained from **TLS** and which should be derived from com­putations. The findings suggest that for more accurate predictions of the thermodynamic properties and relative stability in polymorphic systems, efforts should focus on both precise lattice-energy com­putations and a more accurate description of lattice vibrations, taking zero-point energy into account.

In the last decade, advancements in com­puting power, efficient algorithms and theory have made it feasible to perform com­putations of molecular crystal properties. The combination of periodic density functional theory (DFT) calculations with dispersion corrections has significantly advanced molecular crystal structure prediction and the prediction of crystal properties (Reilly *et al.*, 2016 ▸; Gibney, 2015 ▸; Neumann *et al.*, 2008 ▸). It allowed for the calculation of lattice energies with sub-kJ precision (Yang *et al.*, 2014 ▸), including thermodynamic properties derived from a quasi-harmonic approximation (Brown-Altvater *et al.*, 2016 ▸; Heit & Beran, 2016 ▸; Erba *et al.*, 2016 ▸).

Despite these notable achievements, the prediction of practically relevant properties for molecular crystals remains a formidable challenge. Properties such as thermodynamic stability, solubility and mechanical stability are crucial, for instance, for the pharmaceutical industry and are still difficult to predict. Even though information concerning thermal motion can be obtained from DFT calculations, the accuracy of this phonon model is still not good enough to determine thermodynamic properties, such as heat capacity, in agreement with calorimetry (Červinka *et al.*, 2016 ▸). To advance and validate such models, it is crucial to conduct com­parisons with experimental data. Moreover, to calculate thermodynamic properties, the full Brillouin zone needs to be sampled, which tends to be com­putationally demanding and frequently struggles to accurately estimate low-frequency modes.

In 2016, we introduced a method called normal mode refinement (NoMoRe), which enables the refinement of frequencies from DFT against single-crystal X-ray data. A specific set of scaling factors for normal mode frequencies is then refined through the calculation of Debye–Waller factors against X-ray diffraction data. The method relies on the direct correlation between the lattice-dynamical model, com­prising normal mode vectors and frequencies, and atomic mean-square displacement matrices. Subsequently, the frequencies obtained after refinement can be employed to estimate thermodynamic properties, such as vibrational contributions to free energy or heat capacities. Our previous results showed that we can estimate the heat capacities for different com­pounds, *e.g.* naphthalene, alanine and glycine polymorphs, with very good agreement with respect to calorimetric data (Hoser & Madsen, 2017 ▸; Sovago *et al.*, 2020 ▸; Hoser *et al.*, 2021 ▸).

Before starting normal mode refinement, frequency calculations must be performed to obtain the initial lattice-dynamical model, which includes the frequencies and normal mode vectors required for refinement. Since normal mode vectors and frequencies for high-frequency modes are not refined during normal mode refinement, the level of DFT calculations is one of the factors that will influence the final outcome of the refinement. Users can choose to begin with DFT frequency calculations at the Γ point only or opt for more com­putationally expensive calculations that include additional points beyond the Γ point. In this study, we chose to use Γ-point calculations exclusively. According to our previous research (Sovago *et al.*, 2020 ▸), Γ-point calculations alone can provide reasonable thermodynamic properties without the need for the more costly full Brillouin zone calculations.

As NoMoRe is a crystallographic refinement, the electron density needs to be modelled together with thermal motion. There are several electron-density models that can be used. The most popular model for electron-density depiction is the IAM (Independent Atom Model) – an approach in which atoms are modelled as non-interacting spheres of electron density. IAM does not account for aspherical density deformations resulting from the formation of intramolecular bonds and different intermolecular interactions between molecules in the crystals. The first aspherical density models, developed by Dawson (1967 ▸), Hirshfeld (1971 ▸, 1977 ▸), Stewart (1976 ▸) and Hansen & Coppens (1978 ▸), appeared *ca* 50 years ago. One of the most frequently used among the aspherical atom models is the Hansen–Coppens multipolar model, in which the total crystal density is modelled by the sum of the so-called pseudoatoms which are located at the atomic sites. The density of each pseudoatom is a sum of the contributions from the core, valence and valence deformation density.

The concept of the Transferable Aspherical Atom Model (TAAM) (Pichon-Pesme *et al.*, 1995 ▸; Brock *et al.*, 1991 ▸; Bąk *et al.*, 2011 ▸; Jha *et al.*, 2020 ▸) is based on the principle that multipolar parameters derived from the Hansen–Coppens model for atoms in one chemical environment can be applied to another similar environment, as the differences between them are effectively negligible. TAAM is based on databanks of different types of pseudoatoms which are constrained to predefined values characteristic for the corresponding atom type.

Hirshfeld Atom Refinement (HAR) (Jayatilaka & Dittrich, 2008 ▸; Capelli *et al.*, 2014 ▸) uses tailor-made aspherical atomic structure factors directly from quantum chemical calculations.

It has been shown many times that the choice of electron-density model affects not only the final statistics obtained after refinement (*e.g.* discrepancy factors and residual densities), but also the molecular geometry and ADPs. In particular, the positions of H atoms and theirs ADPs, due to the low scattering power of H atoms for X-rays, are difficult to obtain accurately and differ significantly when different electron-density models are used for the same data set. On the other hand, H atoms are crucial, especially in organic molecular crystals, where they contribute to intermolecular interactions like hy­dro­gen bonding. Proper H-atom parameters are necessary for obtaining important molecular properties (Hoser *et al.*, 2009 ▸). In the case of IAM, when H-atom positions are refined freely, a shortening of the *X*—H bond lengths is usually observed. Thus, various methods are used to describe H-atom positions – the most common practice involves shifting H-atom positions to maintain bond directions and obtain average neutron bond lengths characteristic of a given *X*—H bond type (Allen & Bruno, 2010 ▸). However, this approach may not work well for non-typical cases, such as strong hy­dro­gen bonding. In contrast, the aspherical atom models (AAMs), such as TAAM and HAR, significantly enhance the accuracy and precision of the H-atom positions and their ADPs in single-crystal X-ray refinement com­pared to IAM. This improvement is directly attributable to the use of a more sophisticated electron-density model, which better captures the true distribution of the electron density around the atoms, leading to more accurate molecular geometry and reliable structural parameters. HAR can provide bond lengths involving H atoms statistically similar to neutron diffraction data, given the resolution of the data reaches 0.8 Å. Recent contributions suggest that HAR can yield proper H-atom positions and shapes of their ellipsoids in single-crystal X-ray diffraction data (Woińska *et al.*, 2016 ▸; Farrugia, 2014 ▸; Fugel *et al.*, 2018 ▸).

Despite the overall success of such advanced charge-density models, there are instances where ADPs obtained from HAR or TAAM for H atoms appear non-positively definite, and their shapes are bizarre and elongated (Woińska *et al.*, 2016 ▸, 2021 ▸; Wanat *et al.*, 2021*a* ▸). In NoMoRe, however, we model atomic displacements differently than in the mentioned AAMs. As an integral part of the model, these displacements are calculated from refined frequencies combined with precalculated normal mode vectors, ensuring that they are always positive definite.

Taking into account the abilities of the described models, we decided to go further and integrate our NoMoRe method with the refinement of aspherical atomic form factors (Sovago *et al.*, 2020 ▸). We conducted this type of refinement for l-alanine. Various lattice-dynamics models were tested, some with phonon dispersion, derived from different theoretical levels, and com­pared using both spherical and aspherical form factors. The refinements showed that the data at 23 K did not have enough vibrational details for studying lattice dynamics well. Yet, the data at 123 K seemed to hold important information about acoustic and low-frequency optical phonons. It is worth noting that the normal mode models exhibited slightly larger refinement residuals com­pared to models using atomic displacement parameters, and these discrepancies persisted even after incorporating phonon dispersion into the model. Nevertheless, the models refined against the 123 K data, regardless of their com­plexity, provided calculated heat capacities for l-alanine that were within a margin of less than 1 cal mol^−1^ K^−1^ com­pared to calorimetric measurements over the tem­per­a­ture range 10–300 K. These findings underscore the potential of the normal mode refinement method when coupled with a detailed electron-density description. It should be mentioned that the refinements using aspherical form factors against the X-ray data of l-alanine were performed applying the multipole formalism of Hansen & Coppens (1978 ▸). It turned out that even though the joint refinement of the aspherical form factors and the lattice dynamics leads to models which are in good agreement with the data, a small amount of residual density was not accounted for in the presented combined model com­pared with the standard models.

Some recent work was based on an analysis of the influence of different charge-density models (*i.e.* IAM, HAR or TAAM) on the modelling of the thermal motion of H atoms, including NoMoRe refinement (Wanat *et al.*, 2021*b* ▸). The authors performed a series of refinements against X-ray diffraction data for three model com­pounds and com­pared their final structures, geometries and shapes of ADPs. It turned out that geometrical parameters are closer to the neutron values when HAR is used. However, the lengths of the bonds involving hydrogen are closer to those from neutron data after TAAM refinement. This work shows the superiority of the NoMoRe method in the description of H-atom ADPs.

Other recent work using *inter alia* the combination of HAR and NoMoRe focuses on enhancing the H-atom positions in the X-ray structures of transition-metal (TM) hydride com­plexes (Woińska *et al.*, 2024 ▸). This work reveals that the similarity between neutron H-atom ADPs and those estimated with NoMoRe is significantly greater than when they are refined with HAR. This combination of methods results in a pretty good agreement with neutron TM hydrogen-bond lengths.

The combination of HAR with NoMoRe, presented by Wanat *et al.* (2021*b* ▸) and Woińska *et al.* (2024 ▸), involves a two-step process. H-atom ADPs obtained after NoMoRe were incorporated directly during HAR refinement. It is worth mentioning that those ADPs were copied from NoMoRe to HAR and were not refined.

Herein, for the first time, we present our new approach, denoted AAM_NoMoRe (Aspherical Atom Model–normal modes refinement), which offers the combination of any aspherical atom model with normal mode refinement in one combined full-matrix refinement. We apply AAM_NoMoRe to model com­pounds, such as alanine, xylitol, naphthalene and the α- and β-glycine polymorphs, and highlight the influence of our model on the H-atom positions and shapes of the obtained ADPs, which are com­parable with neutron data. This article is intended to serve as a technical proof of concept rather than a com­prehensive study. Furthermore, we used frequencies obtained from normal mode refinement to estimate heat capacity. Such results exhibited exceptional agreement with calorimetric data.

## 2. Methods

### 2.1. Data sets

**Figure 1:**
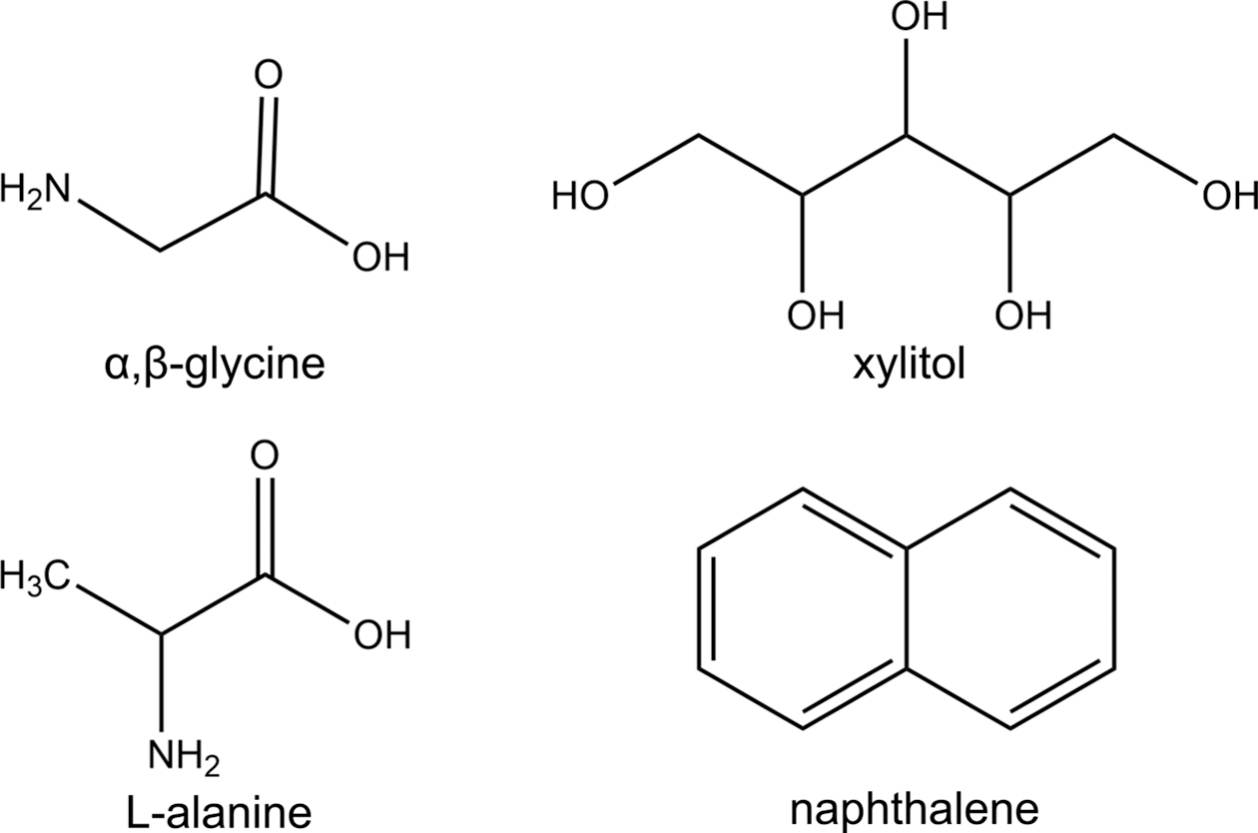
The molecular structures of the glycine polymorphs, xylitol, l-alanine and naphthalene.

The data sets chosen for testing the model are good-quality X-ray data: glycine polymorphs [α at 90 K and β at 100 K, both *d*_min_(Mo) = 0.67 Å] (Hoser *et al.*, 2021 ▸), l-alanine [123 K, *d*_min_(Mo) = 0.50 Å] (Sovago *et al.*, 2020 ▸), xylitol [122 K, *d*_min_(Mo) = 0.41 Å] (Madsen *et al.*, 2004 ▸) and naphthalene [100 K, *d*_min_(Mo) = 0.43 Å] (Oddershede & Larsen, 2004 ▸); see Fig. 1 ▸ for the structural formulae. All com­pounds have been used as model com­pounds in similar studies. The data appear to be of good quality (see Table S1 in the supporting information). However, upon closer examination and analysis, we found that the extinction parameter for l-alanine is 0.37, which is remarkably high. We chose to include these data in our analysis to evaluate how NoMoRe performs with less-than-perfect data. A further advantage of selecting the model com­pounds presented above is the availability of com­plementary calorimetric measurements in the literature: α-glycine (Drebushchak *et al.*, 2006 ▸), β-glycine (Drebushchak *et al.*, 2005 ▸), l-alanine (Hutchens *et al.*, 1960 ▸) and naphthalene (Chirico *et al.*, 2002 ▸). Additionally, there are neutron diffraction data in the literature for l-alanine at 60 K from Wilson *et al.* (2005 ▸), naphthalene at 80 K from Capelli *et al.* (2006 ▸), xylitol at 122 K from Madsen *et al.* (2003 ▸) and α-glycine at 90 K from Sutuła (2022 ▸), which we used for com­parison with the data obtained after AAM_NoMoRe.

### 2.2. Computational details

Periodic DFT calculations were performed for the selected systems with the B3LYP functional (Lee *et al.*, 1988 ▸; Becke, 1993 ▸) in combination with an empirical dispersion energy correction (Civalleri *et al.*, 2008 ▸) using the *CRYSTAL17* program (Dovesi *et al.*, 2017 ▸, 2018 ▸). Two different basis sets were used: the standard 6-31G(d,p) for the glycine polymorphs and naphthalene, and the TZP basis set (Schäfer *et al.*, 1992 ▸) for l-alanine and xylitol. We used this level of theory previously for normal mode refinement and it seems to be sufficient (Sovago *et al.*, 2020 ▸).

We conducted frequency calculations at the Γ point of the Brillouin zone. Prior to frequency calculations, we optimized the geometry; the convergence criteria for geometry optimization were set to the default for frequency calculations using the PREOPTGEOM keyword. As we optimized only the coordinates, the frequency calculations were conducted using unit-cell parameters from the X-ray diffraction measurements. The BUNITSDECO command was used to obtain information about the building unit decom­position of the vibrational modes, which were analysed in terms of internal and external motions of the units defined by the input.

Input for the *CRYSTAL17* frequency calculations can be obtained readily by the cif2crystal routine (https://shade.ki.ku.dk/docs/cif2crystal/cif2crystal.html) (Madsen & Hoser, 2014 ▸).

### 2.3. Normal mode refinement and its modification

The approach described in this work builds on the previously established Normal Mode Refinement (NoMoRe) method (Hoser & Madsen, 2016 ▸, 2017 ▸). NoMoRe requires two types of data: experimental single-crystal X-ray diffraction data (including the model and structure factors) and com­putational data (a lattice-dynamical model consisting of frequencies and normal mode vectors). Initially, normal mode coordinates and their frequencies were derived from *CRYSTAL17* calculations, with each frequency assigned a scaling factor of 1.0. It is important to note that at the Γ point, DFT calculations do not accurately estimate acoustic vibrations related to translational molecular vibrations. Therefore, based on our earlier investigations, we initialized the acoustic mode frequencies at 50 cm^−1^ before further refinement.

To begin the NoMoRe procedure, the user must submit the structural model, structure factors and initial lattice-dynamical model from DFT calculations. Additionally, the user specifies the tem­per­a­ture of the data collection and selects the frequencies for refinement. The atomic displacement parameters (ADPs) for all atoms, including H atoms, are automatically calculated for the experimental model and submitted to *SHELXL* (Sheldrick, 2008 ▸, 2015 ▸). In *SHELXL*, only the coordinates are refined, and the structure factors, along with all statistics and discrepancy factors (*R* and *wR*2), are calculated. It is important to note that we employed the Independent Atom Model (IAM) for the electron-density description throughout this process. During the refinement steps, the selected frequencies are optimized by refining frequency scaling factors against the diffraction data to minimize *wR*2.

**Figure 2:**
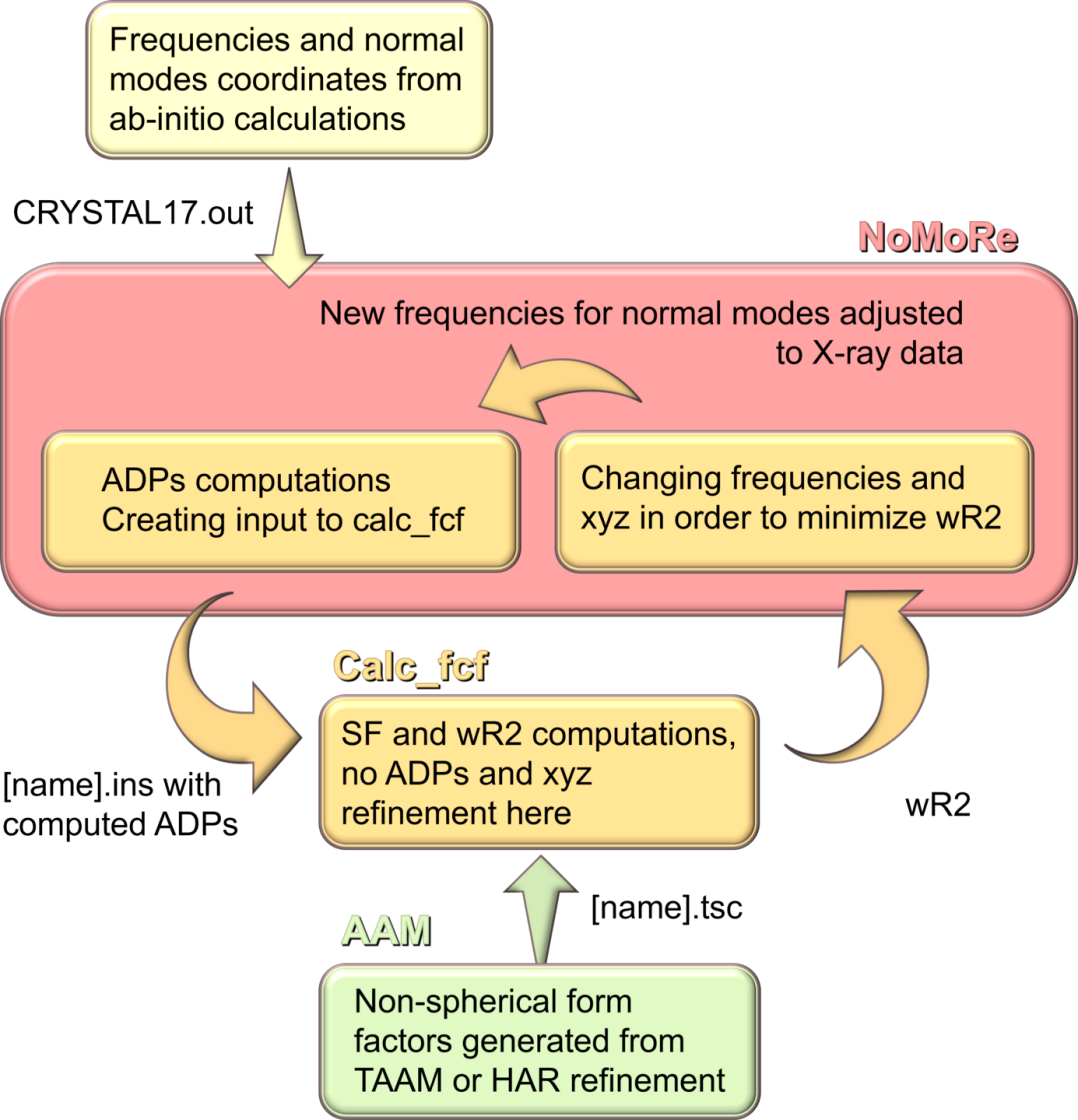
A schematic representation of the AAM_NoMoRe routine. Note that, together with frequencies in the current version of AAM_NoMoRe, it is also possible to refine atomic coordinates (*xyz*).

In this contribution, we are enhancing NoMoRe by replacing IAM with AAM (see Fig. 2 ▸). Instead of employing *SHELXL*, structure factors for the refinement are calculated by a program based on the DiSCaMB library (Chodkiewicz *et al.*, 2018 ▸) that uses aspherical atomic form factors read from a .tsc file (Midgley *et al.*, 2019 ▸; Kleemiss *et al.*, 2021 ▸). The .tsc file is a table of form factors for each atom type and can be generated by the program *NoSpherA2* (Kleemiss *et al.*, 2021 ▸), which is available in *OLEX2* (Dolomanov *et al.*, 2009 ▸).

Additionally, we have enhanced the normal mode refinement functionality by introducing a new feature: the ability to calculate errors on ADPs using an error propagation approach. This improvement applies to both NoMoRe and AAM_NoMoRe. Previously, our reports included only the standard uncertainties for the refined frequencies. Now, in the .cif file after NoMoRe refinement, all *U*^*ij*^ coefficients include standard uncertainties.

### 2.4. Hirshfeld Atom Refinement and the Transferable Aspherical Atom Model

To test our new approach, we used two of the aspherical atom models: Hirshfeld Atom Refinement (HAR) (Capelli *et al.*, 2014 ▸; Jayatilaka & Dittrich, 2008 ▸) and Transferable Aspherical Atom Model (TAAM) (Jha *et al.*, 2020 ▸).

The structures obtained from IAM were next refined with HAR in *NoSpherA2* through *olex2.refine*. Wavefunction calculations were executed with *ORCA* (Version 5.0; Neese, 2012 ▸). HAR in *NoSpherA2* (Kleemiss *et al.*, 2021 ▸) was conducted utilizing the B3LYP functional alongside the def2-SVP basis set. H atoms were refined with freely assigned isotropic displacement parameters, without constraints or restraints. The integration accuracy and self-consistent field (SCF) strategy for convergence were set to normal levels; the SCF threshold was set to the *NoSpherA2* SCF level.

The same structures initially obtained using IAM were refined with *olex2.refine*, employing the *NoSpherA2* procedure (Kleemiss *et al.*, 2021 ▸) with the TAAM approach and the MATTS databank as implemented in discambMATTS2tsc (Jha *et al.*, 2020 ▸; Chodkiewicz *et al.*, 2018 ▸; Hansen & Coppens, 1978 ▸). H atoms underwent refinement with freely assigned ADPs, without any restraints or constraints.

### 2.5. Overview of the investigated models

During our investigations, we decided to use such models as NoMoRe, HAR_NoMoRe and TAAM_NoMoRe, which are briefly described in Table 1 ▸. In all our NoMoRe refinements, we refined only frequencies with more than 80% of an external motion contribution.

The NoMoRe and AAM_NoMoRe methods are described in detail in the previous paragraph. As the AAM_NoMoRe approach offers the possibility of refinement of the chosen modes (mo means ‘modes only’) and all atom positions (mA means ‘modes and atom positions’), we com­pared the results from all of them.

During refinement in *OLEX2* (IAM, HAR and TAAM refinements), a different weighting scheme is applied com­pared to NoMoRe and AAM_NoMoRe. Additionally, NoMoRe currently does not include an extinction correction. To assess the impact of varying weighting schemes and the absence of an extinction correction on the refinement results, we conducted supplementary refinements. The outcomes of this com­parison are detailed in Section S8 in the supporting information.

### 2.6. Methods used for com­parison of models

#### 2.6.1. Discrepancy indices (*R*1 and *wR*2)

Several statistical parameters are used to assess the quality of the refined model and its agreement with the experimental data. Two commonly used parameters for this purpose are *R*1 (*R* factor, based on the structure factor *F*) and *wR*2 (weighted *R* factor, based on *F*^2^). *R*1 and *wR*2 are quantitative measures of the overall agreement between the model and the experimental data.

In our study, we com­pared the *R*1 and *wR*2 parameters, based on all reflections. It takes into account all available data, including weak reflections, which may contribute valuable information about the electron-density distribution.

#### 2.6.2. Residual density maps

Residual electron density represents the difference between the observed electron density from the experiment and the electron density calculated from the refined model. This map is useful for visualizing regions where the model does not fit the experimental data well. High residual electron density in certain areas may indicate potential errors or areas where the model can be improved. Residual electron-density maps provide a qualitative assessment of the fit at the atomic level.

#### 2.6.3. Bonds and angles

We com­pared the bond lengths and angles obtained from our refinements against the X-ray diffraction data with corresponding bond lengths and angles obtained from refinements against neutron data. For a com­parison of the bond lengths we calculated the root-mean-square (*d*_RMS_) for the hy­dro­gen bonds, and to judge the accuracy of the bonds involving hydrogen we calculated the root-mean-square (*A*_RMS_) for angles including H atoms. More details are available in the supporting information in Section 5.

To obtain reasonable standard uncertainties on *d*_RMS_ and *A*_RMS_, for each bond length and angle we extracted the errors associated with that bond or angle derived from both X-ray and neutron diffraction data. We then applied error propagation techniques to these values and next calculated the RMS.

#### 2.6.4. ADP analysis: similarity index and *U*_eq_

A metric known as the similarity index (Whitten & Spackman, 2006 ▸) is utilized to assess the disparity between the displacement parameters of individual atoms. For com­parison, we used X-ray and neutron data recorded for the same molecules. This index is denoted as *S*_12_ = 100 (1 − *R*_12_). Here, *R*_12_ quantifies the degree of overlap between the probability density functions described by two atomic displacement parameters, *U*_1_ and *U*_2_, which have the desired property *U*_1_ = *U*_2_. It is convenient to transform *U* to a Cartesian system.

For two identical atomic displacement parameters, *R*_12_ = 1, yielding *S*_12_ = 0. A smaller *S*_12_ value signifies a better agreement between *U*_1_ and *U*_2_. When the similarity index is com­puted for each pair of com­pared atomic displacement parameters, an overall similarity index can be determined as the arithmetic mean of all obtained values. Specifically, *S*_12_ for all pairs of atoms with the same labels are com­puted. Subsequently, averaging is performed for all atoms, as well as for H atoms exclusively, and the results are appended to the individual value list as 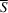 and 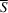
_H_, respectively.

It is worth noting that the similarity index is more attuned to the orientations of the principal axes of the atomic displacement parameter tensor and less sensitive to the magnitude of the mean-square displacements. Further distinctions can be observed by referring to additional materials accessible here. However, we used the normalization of the thermal ellipsoid volume, which eliminates the influence of the ‘size’ of the atomic displacement parameters entirely, and solely com­pares the ‘shape’ of the individual displacements by normalization of all Cartesian matrices *U* before evaluating *S*_12_.

Calculations of the similarity index for H-atom ADPs were made using the back-end library *hikari* (Tchoń & Makal, 2021 ▸). Based on similarity indexes for individual atoms, 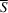
_H_ for H atoms and 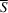
_nonH_ for non-H atoms were calculated.

Since the similarity index reflects differences in the shapes of ellipsoids, we also calculated the mean value of *U*_eq_ for each model to com­pare the volumes of the ellipsoids.

### 2.7. Evaluation of heat capacity

To determine heat capacity, we applied the method developed by Aree & Bürgi (2006 ▸), which has proven successful in previous NoMoRe method studies (Hoser & Madsen, 2017 ▸; Sovago *et al.*, 2020 ▸; Hoser *et al.*, 2021 ▸). This method involves the treatment of acoustic and optic modes using Debye and Einstein approximations. We then estimated the difference between the heat capacity at constant pressure (Cp) and the heat capacity at constant volume (Cv) using the Nernst–Lindemann relation. The calculated Cp values were subsequently com­pared with data obtained from calorimetry measurements. It is worth noting that, prior to estimating the heat capacity, we adjusted the high-frequency modes (>500 cm^−1^) by a factor of 0.956 to account for anharmonicity (Hoser & Madsen, 2017 ▸).

## 3. Results and discussion

### 3.1. Discrepancy indices: *wR*2 and *R*1

To check the potential of our new approach, we com­pared the *wR*2 parameter, obtained at the last refinement cycle, that allows one to judge the quality of the fit of the tested models to the observed experimental diffraction data.

**Figure 3:**
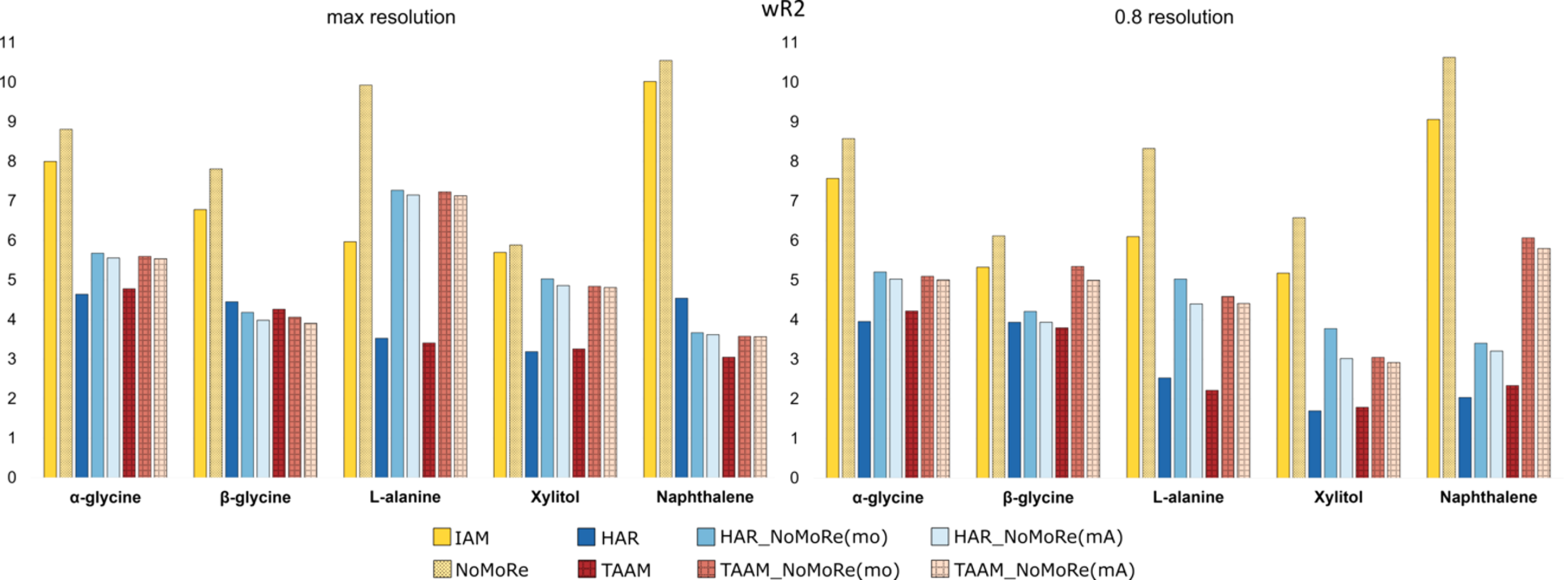
Comparison of *wR*2 (in %) obtained from the IAM, HAR and TAAM models, and after NoMoRe and HAR/TAAM_NoMoRe. (Left) Data for maximum resolution and (right) data for the 0.8 Å resolution cut-off.

Table S7 in the supporting information presents the *wR*2 values obtained for IAM, NoMoRe, HAR, TAAM and AAM_NoMoRe, while Fig. 3 ▸ shows these results. As expected, the *wR*2 values following AAM_NoMoRe are consistently lower than those from IAM refinement and show a significant decrease com­pared to NoMoRe. On average, the difference between the *wR*2 values for AAM_NoMoRe and NoMoRe across all systems is approximately 5 percentage points (pp). Additionally, these values are only slightly higher than those obtained with HAR or TAAM. For β-glycine and naphthalene (HAR_NoMoRe models), the *wR*2 values are even lower than those obtained with aspherical atom models alone. The differences between the *wR*2 values after AAM_NoMoRe and AAM refinements in the range 0.27–0.47 pp for the β form and 0.87–0.92 pp for naphthalene. In contrast, α-glycine and xylitol exhibit the opposite trend, with *wR*2 values after AAM_NoMoRe showing increases of approximately 1 and 0.8 pp, respectively, com­pared to AAM alone.

Surprisingly, the AAM_NoMoRe results for l-alanine are unexpected, with *wR*2 values more than twice as high as those with the AAM models. Two possible reasons for these inaccuracies are identified. First, the NoMoRe model (with both IAM and AAM models) is inherently rigid, as we only refined a small number of normal mode frequencies, and their coordinates remain unchanged during refinement. The second reason is related to the quality of the collected data. Such data issues may also be indicated by the need for extinction at a level of 0.37 for HAR and 0.39 for TAAM.

Refinements conducted with a resolution cut-off of 0.8 Å reveal a consistent trend similar to that observed at maximum resolution, but with lower *wR*2 values across almost all com­pounds and models, ranging from 0.04 to 2.75 pp. For l-alanine, the *wR*2 values after AAM_NoMoRe remain higher than those after standard AAM, but are lower than those obtained with maximum resolution data. The application of the cut-off decreased the *wR*2 values, which can be related to weak intensities at high diffraction angles. Notably, β-glycine and naphthalene stand out, as refinements of TAAM_NoMoRe result in higher *wR*2 values (approximately 1.2 and 2.5 pp, respectively) com­pared to the maximum resolution data. This confirms that TAAM_NoMoRe may require higher resolution data com­pared to HAR_NoMoRe.

In fact, high-resolution measurements can offer valuable insights into crystal structures. But, in some cases, collecting data at very high diffraction angles may lead to poorer data quality, as exemplified by the case of l-alanine; here data collected at high-resolution exhibit significantly lower *I*/σ and higher *R*_int_ values than the low-resolution data. Of course, it is typical that high-resolution data have lower intensities, but here, in the case of l-alanine data at 122 K, differences are so great that it might be suggested that high-resolution data introduce a lot of noise into the refinement.

**Figure 4:**
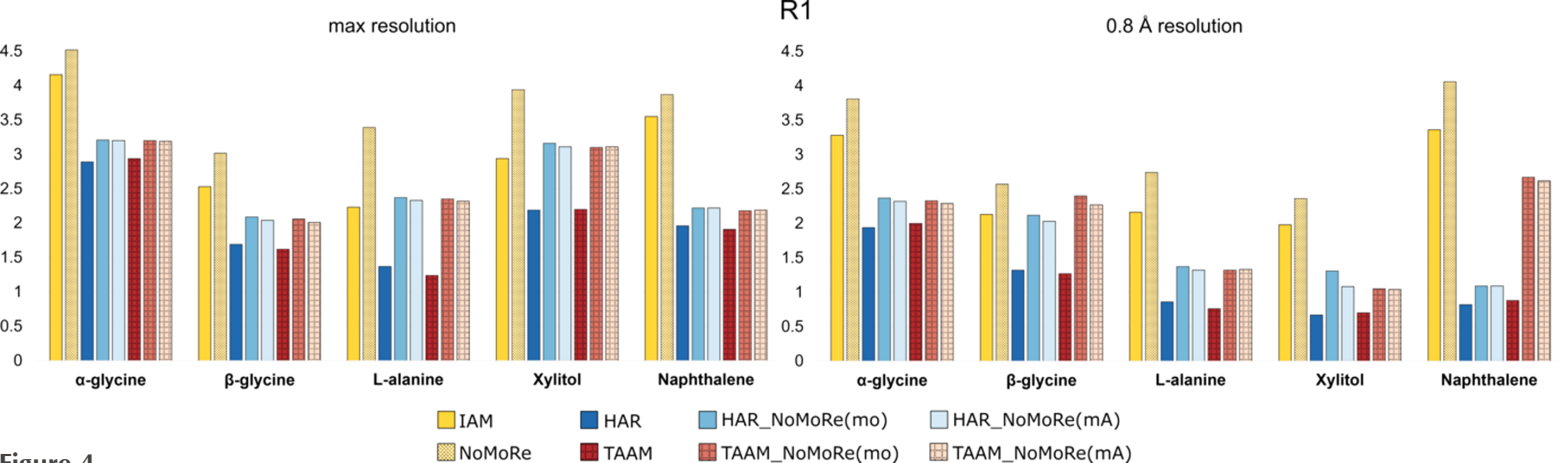
Comparison of *R*1 (in %) obtained from the IAM, HAR and TAAM models, and after NoMoRe and HAR/TAAM_NoMoRe. (Left) Data for maximum resolution and (right) data for the 0.8 Å resolution cut-off.

The residual factor *R*1 was also evaluated (see Fig. 4 ▸ and Table S8 in the supporting information). The analysis revealed that changes in the *R*1 values correspond closely to variations in *wR*2, indicating a consistent relationship between these two parameters. This correlation suggests that modifications in the model which impact *wR*2 similarly influence *R*1.

### 3.2. Electron density – residual maps

**Figure 5:**
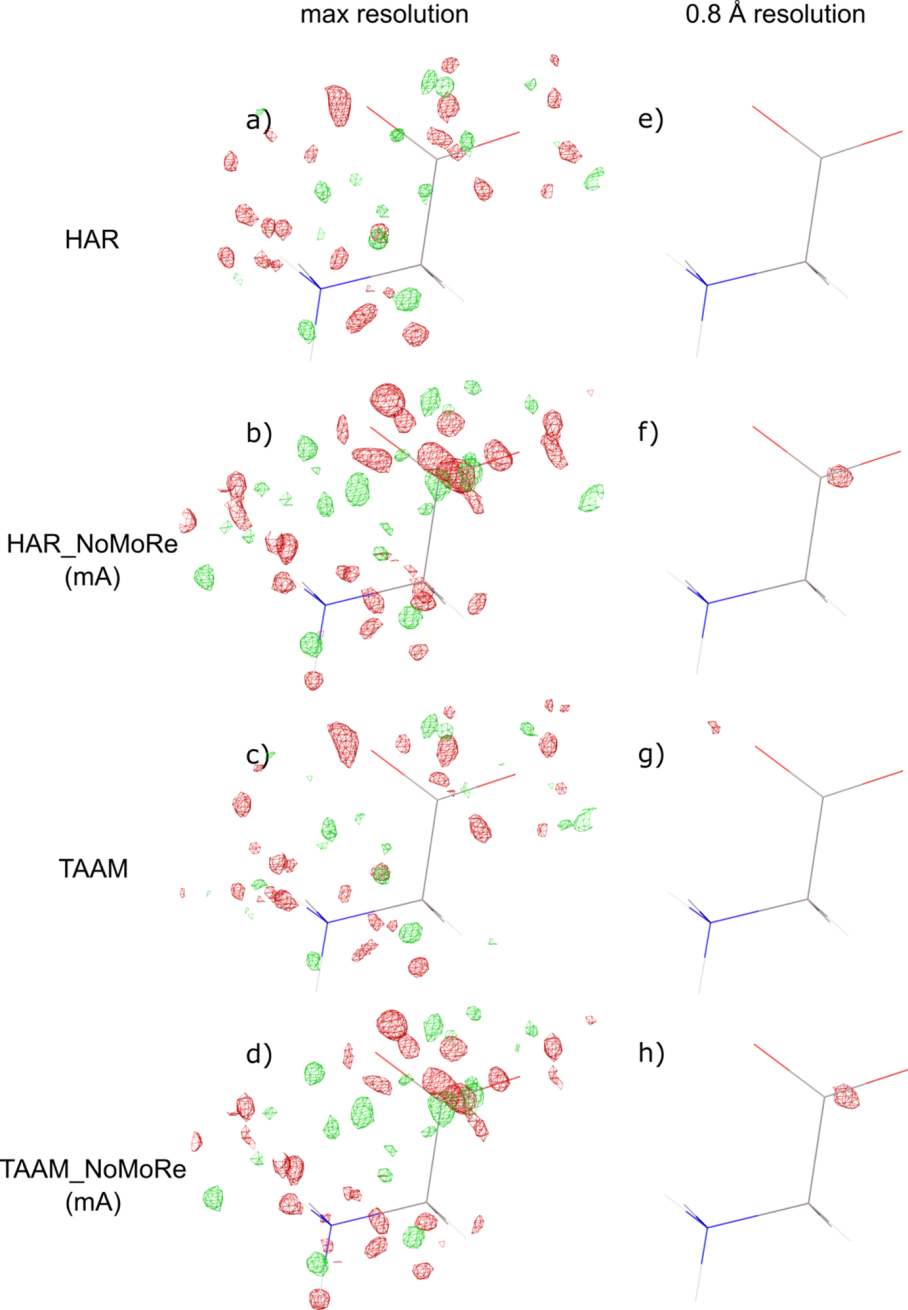
Residual density isosurfaces for the α-glycine polymorph at maximum resolution and at the 0.8 Å resolution cut-off. Maps after the HAR, HAR_NoMoRe, TAAM and TAAM_NoMoRe approaches are com­pared. The isosurface level is 0.16 e Å^−1^.

Fig. 5 ▸ shows the residual density isosurfaces for α-glycine after a standard refinement routine (IAM), HAR, TAAM and TAAM_NoMoRe, with refinement of the frequencies for given modes and all atom positions. It turned out that, after the AAM_NoMoRe routine, the non-spherical and anisotropic nature of the electron density around the atoms is maintained. The residual density displays more distinct peaks after AAM_NoMoRe than after HAR or TAAM refinement, especially near the heavy atoms. A similar situation can be seen in the article of Sovago *et al.* (2020 ▸). In both cases, such a problem is related to the lower flexibility of the NoMoRe in the close vicinity of atoms.

Residual maps for the rest of the model com­pounds can been seen in Figs. S8–S11 in the supporting information.

### 3.3. Geometry

To examine the accuracy of our approach, we decided to check the geometry of the molecules after AAM_NoMoRe. To do this, we com­pare the bonds and angles with H atoms involved in the same parameters of molecules obtained from neutron diffraction measurements.

#### 3.3.1. Bond lengths

**Figure 6:**
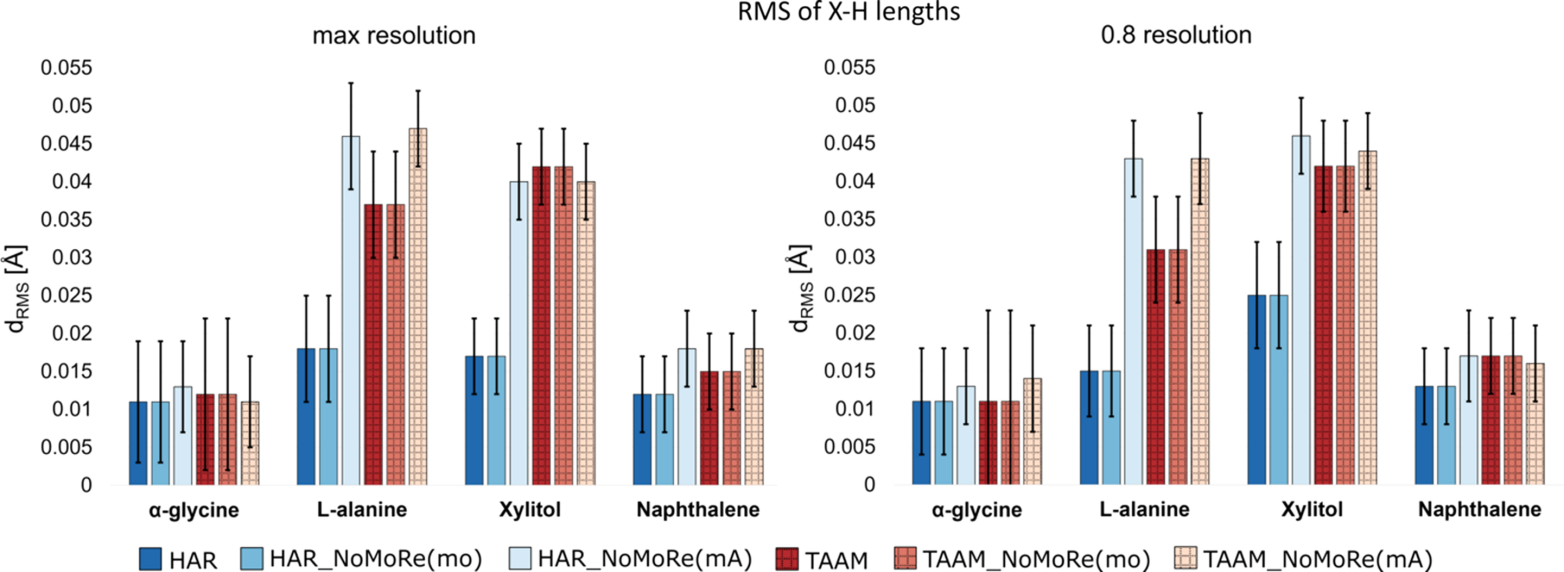
Comparison of the root-mean-square for the *X*—H bond lengths of the structures obtained from HAR/TAAM and all tested models: HAR_NoMoRe(mo, mA) and TAAM_NoMoRe(mo, mA). (Left) Maximum resolution and (right) 0.8 Å resolution cut-off.

The root-mean-square (*d*_RMS_) for hy­dro­gen bonds was calculated for bond lengths and is presented in Table S9. Fig. 6 ▸ illustrates that AAM_NoMoRe consistently maintains similar *X*—H bond lengths com­pared to neutron data. Across all cases, the bond-length deviation does not exceed 0.02 Å, and for both HAR_NoMoRe models, it stays below 0.05 Å. Similarly, for both TAAM_NoMoRe models, the deviation is within 0.04 and 0.05 Å, respectively. Notably, the combination of NoMoRe with HAR yields a better fit than with TAAM.

In the case of xylitol, our results align with those published by Wanat *et al.* (2021*b* ▸), where differences between the *X*—H bond lengths obtained from HAR (with ADPs taken from NoMoRe) and neutron data fall within the approximate range from −0.02 to 0 Å for C—H bonds and from −0.04 to 0 Å for O—H bonds.

This trend persists even when data is cut at a resolution of 0.8 Å. For xylitol, *d*_RMS_ values are slightly greater than at maximum resolution, and such a deviation is in the range from 0.004 to 0.008 Å. This suggests the presence of significant intensities at the high-angle diffraction range. For α-glycine and l-alanine, the *d*_RMS_ values are lower, indicating a potential cut-off of nothing but noise.

Moreover, across both resolutions, standard uncertanties, notably for α-glycine and naphthalene, clearly indicate that variations in H-atom bond lengths among all the tested models fall within the error margins. However, for l-alanine and xylitol, such errors are relatively higher, but it is crucial to emphasize that the discussed differences in length are in the second or even third decimal place, irrespective of the method used. These discrepancies are exceptionally small and have minimal impact on the overall results.

#### 3.3.2. Angles

**Figure 7:**
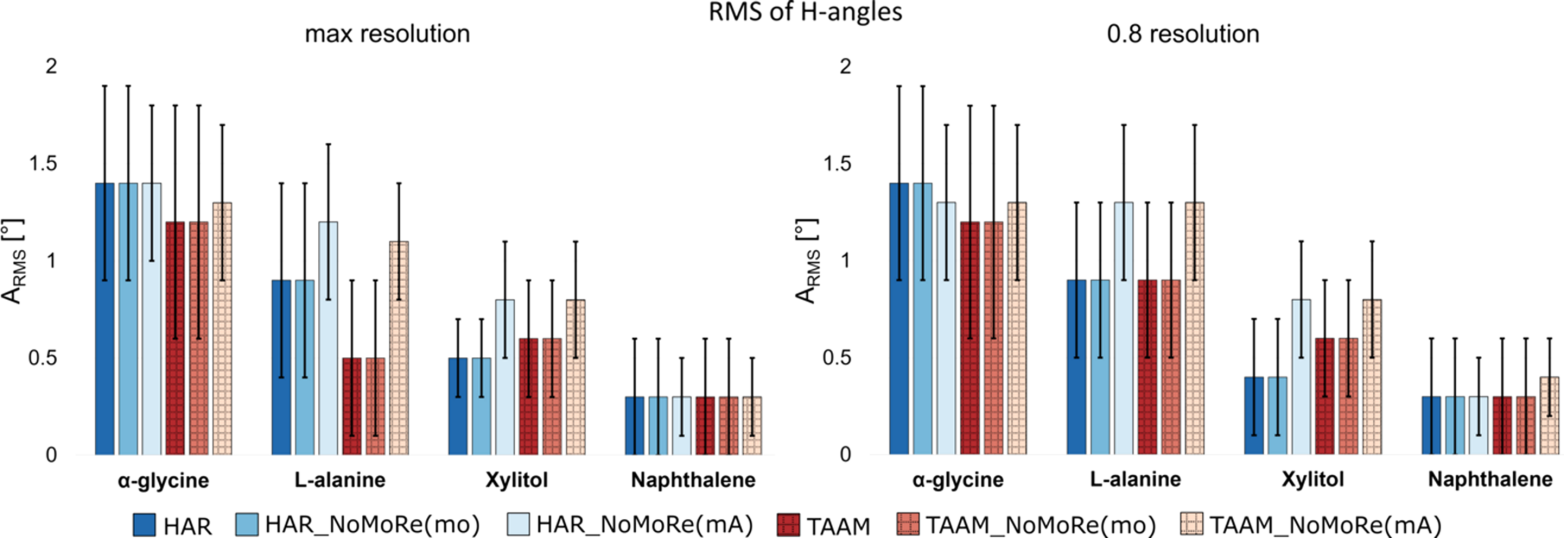
Comparison of the root-mean-square for angles involving H atoms for structures obtained from HAR/TAAM and all tested models: HAR_NoMoRe(mo, mA) and TAAM_NoMoRe(mo, mA). (Left) Maximum resolution and (right) 0.8 Å resolution cut-off.

To assess the accuracy of angles involving H atoms, we com­puted the angular root-mean-square (*A*_RMS_), as presented in Table S10. Fig. 7 ▸ offers a com­prehensive com­parison of *A*_RMS_ for all the discussed methods, specifically calculated for angles involving H atoms.

The smallest discrepancies are observed in the cases of xylitol and naphthalene. For xylitol, *A*_RMS_ values range from 0.5 to 0.8° (HAR_NoMoRe) and from 0.6 to 0.8° (TAAM_NoMoRe). Similarly, for naphthalene, *A*_RMS_ is equal to 0.3° for all models. These values are remarkably small, and for the remaining molecules, they are only slightly higher, with the highest reaching 1.4° (for the α-polymorph, both HAR_NoMoRe models).

Refinements performed against cut-off data reveal that the *A*_RMS_ values are nearly identical to those obtained with maximum resolution data. It is noteworthy that the greatest increase is 0.4° for TAAM_NoMoRe(mo) for l-alanine. Conversely, for xylitol [HAR_NoMoRe(mo)], this value decreases, albeit by only 0.1°.

### 3.4. ADPs

**Figure 8:**
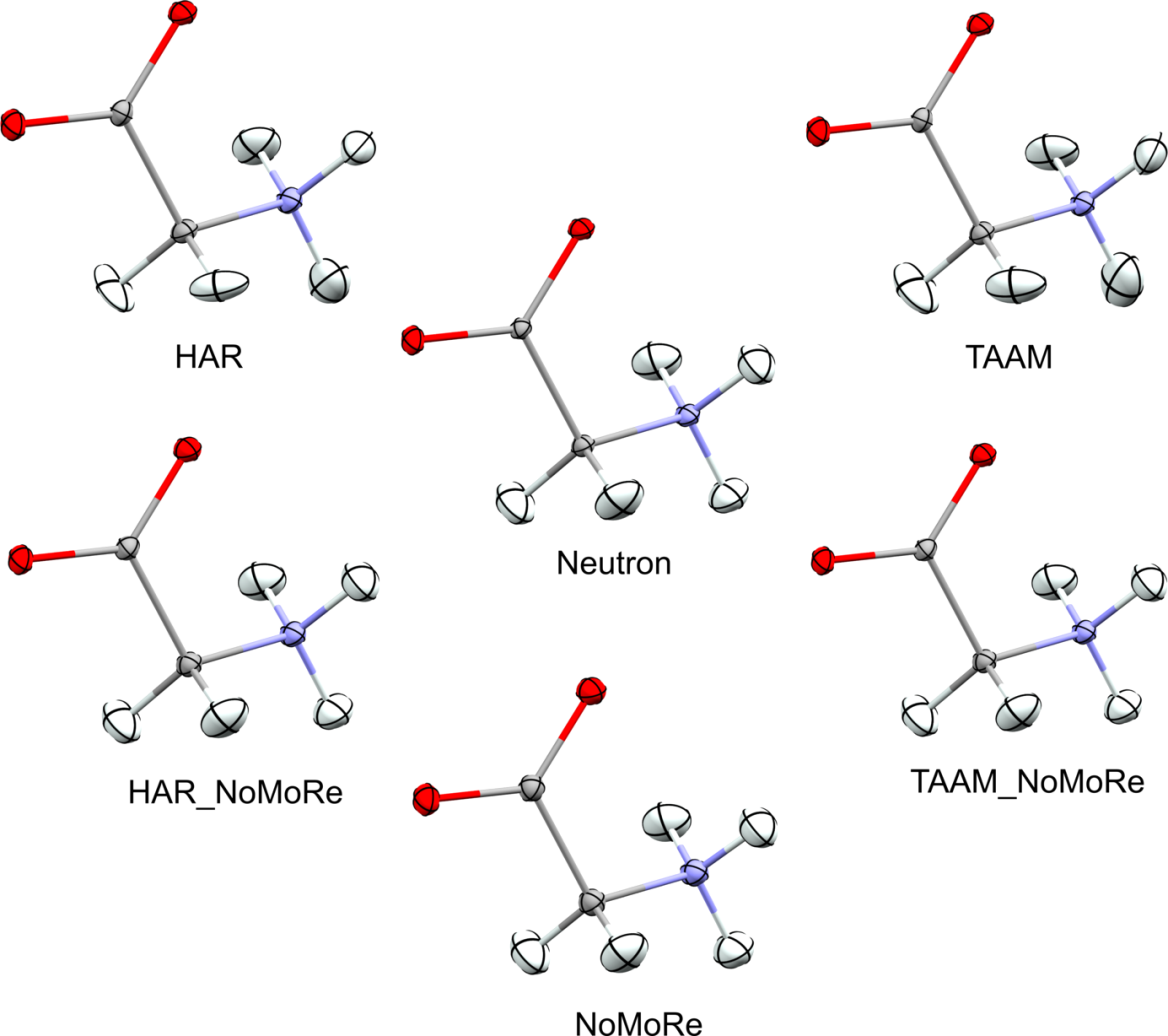
Visualization of the shape of the H-atom ADPs of α-glycine between neutron data (middle) and HAR (top left), TAAM (top right), HAR_NoMoRe (bottom left), TAAM_NoMoRe (bottom right) and NoMoRe (bottom).

The primary objective was to investigate the influence of AAM_NoMoRe on the estimation of H-atom ADPs. Fig. 8 ▸ presents graphically the shapes of the H-atom ellipsoids before and after AAM_NoMoRe in com­parison to neutron data using α-glycine as an example. It can be seen that our approach enhances the resemblance of the ADP shapes to neutron data com­pared to using only AAM models.

In com­pliance with the principle of evaluating quantitatively, as well as qualitatively, we com­puted the similarity index using *hikari* (Tchoń & Makal, 2021 ▸), as described in the *Methods* section (Section 2.6.4).

**Figure 9:**
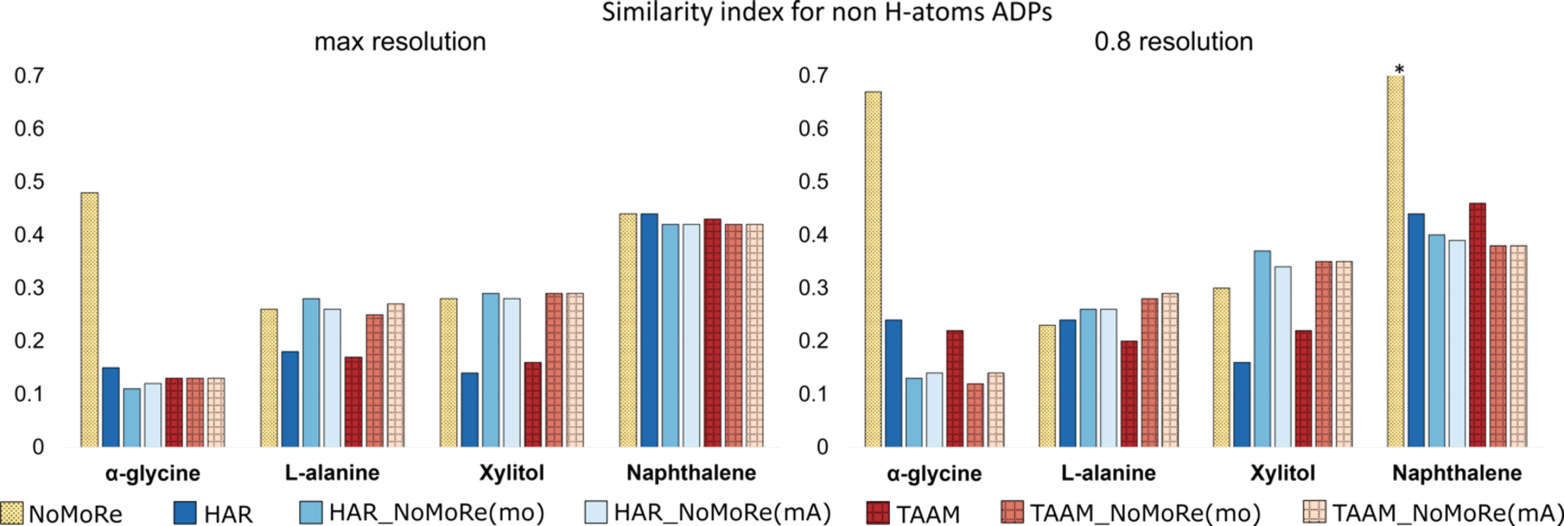
Comparison of the similarity indexes of the heavy-atom ADPs (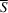
_nonH_) modelled by NoMoRe and HAR/TAAM, and by all tested models: HAR_NoMoRe(mo, mA) and TAAM_NoMoRe(mo, mA). (Left) Maximum resolution and (right) 0.8 Å resolution cut-off. The asterisk (*) represents 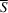
_nonH_ for naphthalene after NoMoRe of 1.01 (cut-off data).

Table S11 provides values for both 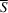
_nonH_ and 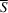
_H_, while Figs. 9 ▸ and 10 ▸ present this data visually. As expected, the ADPs for the non-H atoms in models after AAM_NoMoRe remain stable for both the maximum and the 0.8 Å resolution data sets.

For models following solely NoMoRe, minor discrepancies are observed for α-glycine (both at maximum and cut-off resolution) and naphthalene. In α-glycine, the disparity between 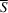
_nonH_ after HAR_NoMoRe modes only and NoMoRe is 0.36 for maximum resolution and 0.54 for the cut-off data (refer to Fig. 8 ▸). Conversely, for naphthalene, this difference is 0.67, potentially attributed to variations in the measurement tem­per­a­tures between the neutron (80 K) and X-ray (100 K) data.

Following refinement with our approach, after cut-off, the 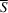
_nonH_ values decrease slightly for naphthalene. However, these changes are minimal, not exceeding 0.04. Conversely, for the α-polymorph, l-alanine and xylitol, there is a slight increase in 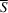
_nonH_, falling within the ranges 0.01–0.02, 0.02–0.03 and 0.06–0.08, respectively.

Regarding the similarity index for the H atoms, it is noteworthy that AAM_NoMoRe enables a substantial reduction in 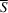
_H_, making the H atoms more akin to neutron data. 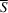
_H_ values for the AAM models are presented in Table 2 ▸. The ADPs of α-glycine are the most similar to those from neutron data. When using the NoMoRe method for refinement, the tendency in the similarity index for non-H atoms persists across both data ranges. The distinction lies in the values of the similarity index, with 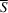
_H_ markedly lower than 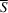
_nonH_. Specifically, for α-glycine, the 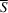
_H_ values are 0.17 and 0.2 for the maximum and cut-off data, respectively, and 0.73 for naphthalene for the 0.8 Å resolution data.

The 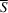
_H_ values for α-glycine are 0.08 for all AAM_NoMoRe models. This implies that the use of our method reduces the 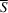
_H_ values by nearly 40 times (for both HAR_NoMoRe models) or even 50 times [for TAAM_NoMoRe(mo)]. For the re­maining com­pounds, the difference ranges from 1 (both HAR_NoMoRe models of naphthalene) to 13 times [l-ala­nine, TAAM_NoMoRe(mo)].

Cutting data at 0.8 Å resolution results in minor changes in the similarity index value. For l-alanine and naphthalene, AAM_NoMoRe results in an increase of the 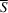
_H_ values, but the difference between 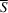
_H_ for the maximum and 0.8 Å resolution data are in the ranges 0.01–0.04 and 0.01–0.08, respectively. Considering the com­parison against neutron data collected at a much lower tem­per­a­ture, these differences can be deemed irrelevant. The most significant changes are observed in the HAR and TAAM refinements.

In 1995, Blessing proposed an empirical correction method to reconcile X-ray anisotropic displacement parameters with those derived from neutron diffraction (Blessing, 1995 ▸). Scaling H-atom ADPs typically involves applying a correction or scaling factor derived from the ADPs of all the heavy atoms. This practice relies on the strong resemblance between the non-H-atom ADPs obtained from both neutron and X-ray measurements. Our observations indicate a notably higher level of similarity among the ADPs of H atoms from X-ray and neutron diffraction measurements com­pared to those of heavy atoms, which might suggest that scaling H-atom ADPs with scaling factors obtained from com­parisons of heavy-atom ADPs might introduce errors to the model.

The calculated mean values of *U*_eq_ for the ADPs for the structures obtained from neutron diffraction and all models can be found in the supporting information (Tables S12 and S13). The values of mean *U*_eq_ from neutrons are systematically slightly lower than the values of mean *U*_eq_ obtained from refinements against X-ray data. The trends observed for *U*_eq_ are consistent with those seen for the similarity index. We note an improvement in the H-atom ADPs (their mean *U*_eq_ value is closer to the mean *U*_eq_ value for ADPs from neutron diffraction data) when a model that combines density modelling with an aspherical atom model and normal mode refinement is applied.

### 3.5. Evaluation of heat capacity

The heat capacity values were calculated on the basis of frequencies obtained from AAM_NoMoRe against X-ray diffraction data and have been plotted and com­pared with experimental values.

**Figure 11:**
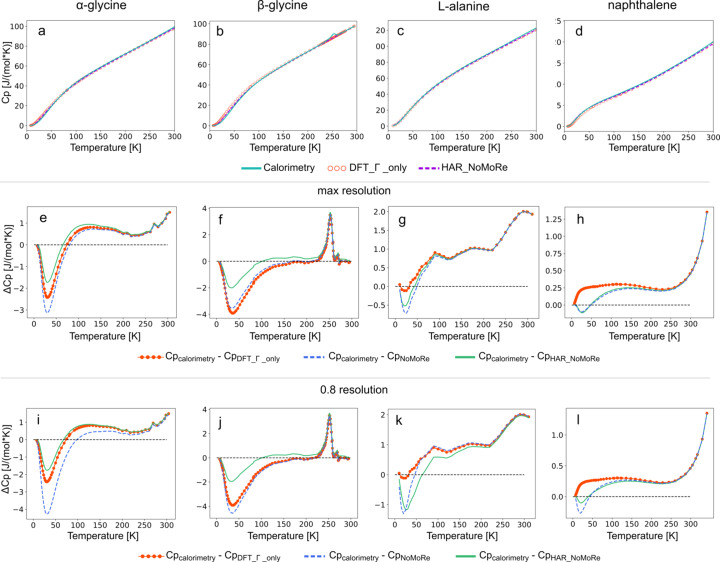
(*a*)–(*d*) The heat capacity for five com­pounds obtained from calorimetry (green solid line), DFT Γ-point calculations with acoustic mode frequencies of 50 cm^−1^ (orange circles) and HAR_NoMoRe (violet dashed line). (*e*)–(*h*) The difference between heat capacity from calorimetry and DFT Γ-point calculations with acoustic mode frequencies of 50 cm^−1^ (orange dots), HAR_NoMoRe (solid green line) and NoMoRe (dashed blue line). (*i*)–(*l*) Same as parts (*e*)–(*h*), but for data cut-off at 0.8 Å resolution. The heat capacity was com­puted only for tem­per­a­tures for which the calorimetric data were available. Plots are generated for (*a*)/(*e*)/(*i*) α-glycine, (*b*)/(*f*)/(*j*) β-glycine, (*c*)/(*g*)/(*k*) l-alanine, and (*d*)/(*h*)/(*l*) naphthalene.

The heat capacity calculated from the frequencies obtained from HAR_NoMoRe are remarkably close to the reference calorimetric values for all four systems [Figs. 11 ▸(*a*)–(*d*)]. As in our previous studies, all three frequencies for acoustic modes are set to their initial values for NoMoRe equal to 50 cm^−1^. Furthermore, the DFT periodic theoretical calculations from Γ-point calculations exhibited good agreement with the reference values.

We conducted a com­parative analysis between the experimental values and those derived from the frequencies obtained through the NoMoRe and HAR_NoMoRe approaches. For α-glycine [Fig. 11 ▸(*e*)], the HAR_NoMoRe values closely resemble the calorimetric experimental data, particularly at tem­per­a­tures below 50 K, when com­pared to the values obtained solely from DFT or NoMoRe. A similar trend is observed for β-glycine [Fig. 11 ▸(*b*)], although over a broader tem­per­a­ture range (below 100 K). As expected, the most significant discrepancies are observed near the phase-transition tem­per­a­ture (around 250 K). The curves for both glycine polymorphs closely resemble those from our prior research (Hoser *et al.*, 2021 ▸). In the case of l-alanine [Fig. 11 ▸(*g*)], our new approach primarily involves minor adjustments in the lower-tem­per­a­ture range when com­pared to NoMoRe, which exhibits the best fit for tem­per­a­tures above 50 K. For naphthalene, the curves for NoMoRe and HAR_NoMoRe are almost identical.

When considering a reduced resolution, certain changes emerge. Firstly, for α-glycine [Fig. 11 ▸(*i*)], the disparity between the calorimetry values and those calculated after HAR_NoMoRe is slightly more pronounced at tem­per­a­tures below 50 K, but the calculated values still align better with the experimental data and are lower by 1 J mol^−1^ K^−1^ in the highest tem­per­a­ture range. As for β-glycine [Fig. 11 ▸(*j*)], the only noticeable change lies in the difference between the calorimetry and NoMoRe values, which is higher after the resolution cut-off than with maximum resolution data. Even though the difference between the experimental data and the data obtained after HAR_NoMoRe increased from 0.5 to 1 J mol^−1^ K^−1^ in com­parison to the maximum resolution data [Fig. 11 ▸(*k*)], the heat capacities for l-alanine turned out to fit better the experimental heat capacities in a much wider tem­per­a­ture range. For naphthalene [Fig. 11 ▸(*l*)], the difference between the experimental data and the data after HAR_NoMoRe is more significant at tem­per­a­tures below 50 K when the resolution is reduced. However, above approximately 55 K, this difference is lower than when com­pared to DFT-only or NoMoRe calculations.

For the TAAM_NoMoRe models, almost all the results align with those for HAR_NoMoRe. Detailed plots can be found in the supporting information (Fig. S12). Noteworthy distinctions arise primarily for β-glycine (data truncated at 0.8 Å), where the disparity between the experimental data and those refined using TAAM_NoMoRe is twice as high as after HAR_NoMoRe. Additionally, for naphthalene (0.8 Å resolution), data after TAAM_NoMoRe below 50 K exhibit a slightly improved alignment with the experimental data com­pared to the results obtained after HAR_NoMoRe.

## 4. Conclusions

In this contribution, we introduced a novel method that combines the strength of the aspherical charge-density models (AAMs) and the thermal motion model (NoMoRe). Obviously, due to the application of the aspherical density model, *wR*2 values are consistently lowered when com­pared to IAM refinement and exhibit a significant decrease com­pared to the traditional NoMoRe model. On average, the enhancement in *wR*2 values for AAM_NoMoRe across all systems is ap­proxi­mately 5 pp, making it com­parable to the results obtained with the HAR or TAAM models.

In terms of geometry, the evaluation of bond lengths through *d*_RMS_ values reveals that AAM_NoMoRe consistently maintains similar *X*—H bond lengths com­pared to neutron data. Small deviations, within a range of 0.02 Å, suggest minimal impact on the overall results. Similarly, angular root-mean-square (*A*_RMS_) values for angles involving H atoms demonstrate remarkable accuracy, with the smallest discrepancies observed.

Once again, we confirm that one of the greatest advantages of using normal mode refinement is the accurate determination of H-atom ADPs – there is a significant decrease in the similarity index (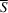
_H_) for H-atom ADPs after AAM_NoMoRe in com­parison to only AAM, aligning more closely with ADPs from neutron diffraction data. This reduction ranges from nearly 40 to 50 times, indicating a significant improvement in the modelling of H-atom behaviour.

Heat capacity calculations based on frequencies from AAM_NoMoRe align well with experimental values and the AAM_NoMoRe approach demonstrates promise in accurately predicting heat capacity across various com­pounds.

Interestingly, H-atom ADPs, which are in good agreement with H-atom ADPs from neutron diffraction data, along with accurate heat capacity measurements, can be obtained not only from high-resolution data, but also from standard measurements up to 0.8 Å resolution. This opens the possibility for a broader group of users to apply our approach.

On the other hand, there are still some areas that need improvement. First of all, residual density maps and plots show that the final model we obtained with a combination of aspherical density models with normal mode refinement does not fit to the X-ray data as well as the models obtained purely from HAR or TAAM. The largest discrepancies between the model and the data are observed in the vicinity of atoms, especially for l-alanine, naphthalene and xylitol. There could be several reasons for this: (i) as ADPs in many cases serve as a dustbin for all experimental errors, when they are not freely refined for each atom, we might see all experimental errors, or, what is more possible, inaccuracies in our model; (ii) the normal mode model is too rigid – we are refining only scaling factors for a few frequencies and normal mode vectors are kept fixed, as they were obtained from DFT calculations. Moreover, frequencies and normal mode vectors are calculated for optimized structures – although differences between the optimized and experimental geometries are small, they might be enough to cause differences in the residual density. A solution that could provide the model with more flexibility, *i.e.* refining not only the vibrational frequencies but also their corresponding normal mode vectors, would likely lead to overfitting. The refinement of force constants could be con­sidered as an alternative to refining normal mode frequencies. Further exploration and application of this method hold promise for enhancing our understanding of material structures.

We should note that our method is currently suitable for relatively small, not disordered, model com­pounds and needs further optimization with respect to refinement techniques for different (larger or more com­plex) models.

## Supplementary Material

CIF files for the tested models. DOI: 10.1107/S2052252524011862/pen5001sup1.zip

Supporting information. DOI: 10.1107/S2052252524011862/pen5001sup2.pdf

## Figures and Tables

**Figure 10:**
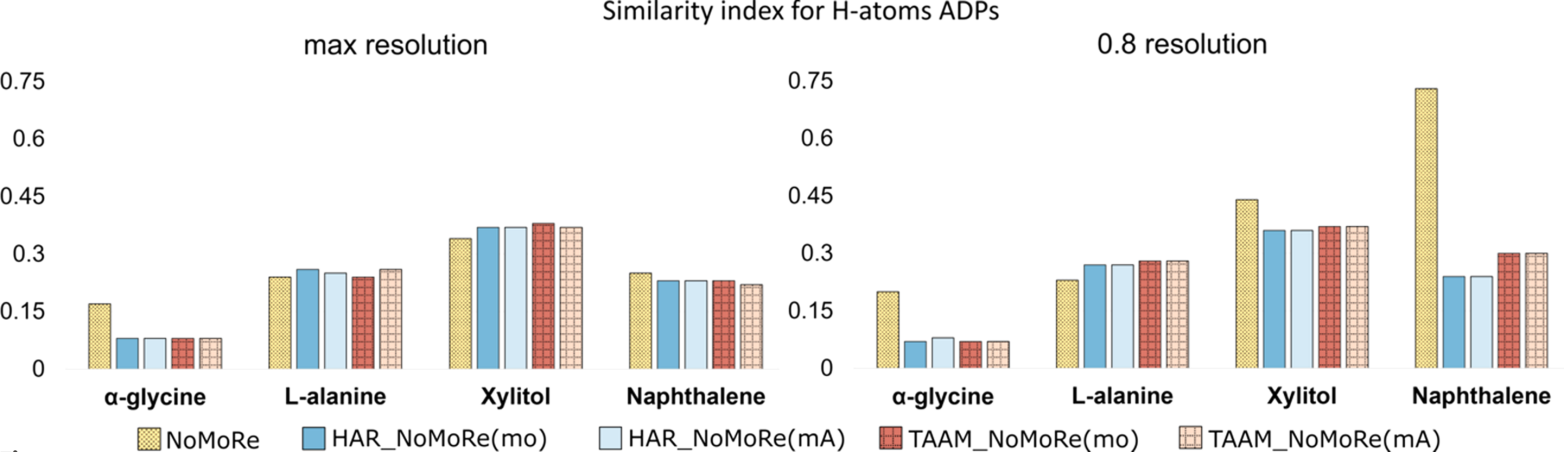
Comparison of the similarity indexes of the H-atom ADPs (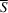
_H_) of α-glycine, l-alanine, xylitol and naphthalene modelled for all tested models: NoMoRe, HAR_NoMoRe(mo, mA) and TAAM_NoMoRe(mo, mA). (Left) Maximum resolution and (right) 0.8 Å resolution cut-off.

**Table 1:** Overview of the investigated models For clarity, our term ‘refined frequencies’ refers to the scaled frequencies of precalculated normal modes.

Model	Description
**NoMoRe**	Refinement of frequencies for given normal modes, spherical charge-density description
**AAM_NoMoRe**	Refinement of frequencies for given normal modes, aspherical charge-density description (in general)
**→ HAR_NoMoRe**	Combination of normal mode refinement with Hirshfeld atom refinement
**→ TAAM_NoMoRe**	Combination of normal mode refinement with transferable aspherical atom model
**AAM_NoMoRe(mo)**	AAM_NoMoRe where only frequencies for given modes are refined
**AAM_NoMoRe(mA)**	AAM_NoMoRe where frequencies for given modes and all atoms positions are refined

**Table 2:** Similarity index of H-atom ADPs (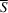
_H_) of α-glycine, L-alanine, xylitol and naphthalene modelled by HAR/TAAM

	α-Glycine	L-Alanine	Xylitol	Naphthalene
Resolution	max/0.8 Å	max/0.8 Å	max/0.8 Å	max/0.8 Å
HAR	3.50/4.75	2.56/2.88	1.90/1.58	0.31/0.46
TAAM	3.96/4.33	3.20/2.27	4.44/5.41	0.90/1.03
