# Supplementary material for: Advancing dynamic quantum crystallography: enhanced models for accurate structures and thermodynamic properties
Source: IUCrJ. 2025 Jan 1;12(Pt 1):123–36. doi: 10.1107/S2052252524011862 (PMC11707699; doi:10.1107/S2052252524011862)
Supplement: Supplementary file 2 [file m-12-00123-sup2.pdf]

IUCrJ

Volume 12 (2025)

Supporting information for article:

Advancing dynamic quantum crystallography: enhanced models for accurate structures and thermodynamic properties

Helena Butkiewicz, Michał Chodkiewicz, Anders Ø. Madsen and Anna A. Hoser

S1. Crystallographic data and refinement parameters

Table S1 Crystallographic data and refinement parameters of chosen model compounds, Independent Atom Model, max resolution.

Identification code	α-glycine	β-glycine	L-alanine	xylitol	naphthalene
Empirical formula	C2H5NO2	C2H5NO2	C3H7NO2	C5H12O5	C10H8
Formula weight/ g/mol	75.07	75.07	89.09	152.15	128.17
Temperature/K	90	100	123	122	100
Crystal system	monoclinic	monoclinic	orthorhombic	orthorhombic	monoclinic
Space group	$P\ 2_1/n$	$P\ 2_1$	$P\ 2_12_12_1$	$P\ 2_12_12_1$	$P\ 2_1/c$
a/Å	5.0880(3)	5.0755(2)	5.9534(5)	8.2625(2)	7.8248(2)
b/ Å	11.7957(5)	6.1735(2)	12.2772(10)	8.8996(2)	5.93490(10)
c/ Å	5.4609(3)	5.3879(2)	5.7882(5)	8.9228(2)	8.0997(2)
α/°	90	90	90	90	90
β/°	111.987(7)	113.423(4)	90	90	114.441(2)
γ/°	90	90	90	90	90
Volume/ $Å^3$	303.91(3)	154.911(11)	423.07(6)	656.12(3)	342.438(14)
Z	4	2	4	4	2
ρ_{calc}g/cm^3	1.641	1.609	1.399	1.540	1.243
μ/mm^{-1}	0.146	0.143	0.117	0.139	0.070
F(000)	160.1	80.1	192.2	328.3	136
Radiation	Mo Kα (λ = 0.71073)				
2Θ range for data collection/°	6.9 to 64.36	8.24 to 64.56	6.64 to 90.56	6.46 to 119.92	5.72 to 109.72
Reflections collected	5155	9901	3548	9942	4368
Independent reflections	1033[R_{int} = 0.0265, R_{sigma} = 0.0349]	1046[R_{int} = 0.0311, R_{sigma} = 0.0141]	3546[R_{int} = 0.046, R_{sigma} = 0.0247]	9942[R_{int} = 0.0171, R_{sigma} = 0.0317]	4368[R_{int} = 0.0305, R_{sigma} = 0.0198]
Data/restraints/parameters	1033/0/66	1046/1/66	3546/ 0/ 84	9942/0/139	4368/0/62
Goodness-of-fit on F^2	1.1197	1.1115	1.6500	1.1081	1.155
Final R indexes [I ≥ 2σ (I)]	R1 = 0.0337 wR2 = 0.0760	R1 = 0.0249 wR2 = 0.0675	R1 = 0.0215 wR2 = 0.0593	R1 = 0.0239 wR2 = 0.0557	R1 = 0.0300 wR2 = 0.0977
Final R indexes [all data]	R1 = 0.0416 wR2 = 0.0800	R1 = 0.0253 wR2 =0.0679	R1 = 0.0223 wR2 = 0.0597	R1 = 0.0298 wR2 = 0.0570	R1 = 0.0355 wR2 = 0.1005
Largest diff. peak/hole /e $Å^{-3}$	0.45/-0.28	0.05/-0.20	0.27/-0.24	0.32/-0.26	0.58/-0.21
Flack parameter	-	0.4(3)	-0.06(14)	0.01(15)	-
Weights	0.014571 0.137434	0.032407 0.041117	0.021236 0	0.016149 0	0.0572 0.0064
Extinction	-	-	0.279(19)	-	-

Table S2 Refinement parameters of chosen model compounds after HAR refinement. Max resolution.

Identification code	α-glycine	β-glycine	L-alanine	xylitol	naphthalene
Data/restraints/parameters	1033/0/91	1046/1/91	3546/ 0/ 119	9942/0/200	4368/0/82
Goodness-of-fit on F^2	1.0383	1.2309	1.2738	0.6356	0.6021
Final R indexes [I ≥ 2σ (I)]	R1 = 0.0214<br>wR2 = 0.0432	R1 = 0.0166<br>wR2 = 0.0444	R1 = 0.0129<br>wR2 = 0.0351	R1 = 0.0160<br>wR2 = 0.0298	R1 = 0.0140<br>wR2 = 0.0408
Final R indexes [all data]	R1 = 0.0289<br>wR2 = 0.0463	R1 = 0.0169<br>wR2 =0.0445	R1 = 0.0137<br>wR2 = 0.0353	R1 = 0.0219<br>wR2 = 0.0319	R1 = 0.0196<br>wR2 = 0.0454
Largest diff. peak/hole /e $Å^{-3}$	0.18/-0.19	0.10/-0.10	0.14/-0.13	0.16/-0.15	0.14/-0.10
Flack parameter	-	0.4(3)	-0.06(14)	0.02(15)	-
Weights	0 0	0.020718<br>0.007971	0 0	0.013087<br>0.003459	0.038926<br>0.01216
Extinction	-	-	0.368(13)	0.0393(15)	-

Table S3 Refinement parameters of chosen model compounds after TAAM refinement, max resolution.

Identification code	α-glycine	β-glycine	L-alanine	xylitol	naphthalene
Data/restraints/parameters	1033/0/91	1046/1/91	3546/ 0/ 119	9942/0/200	4368/0/82
Goodness-of-fit on F^2	1.0711	1.2359	1.1346	0.6400	0.9821
Final R indexes [I ≥ 2σ (I)]	R1 = 0.0219<br>wR2 = 0.0448	R1 = 0.0159<br>wR2 = 0.0425	R1 = 0.0117<br>wR2 = 0.0338	R1 = 0.0161<br>wR2 = 0.0305	R1 = 0.0135<br>wR2 = 0.0292
Final R indexes [all data]	R1 = 0.0294<br>wR2 = 0.0478	R1 = 0.0162<br>wR2 =0.0426	R1 = 0.0124<br>wR2 = 0.0341	R1 = 0.0220<br>wR2 = 0.0326	R1 = 0.0191<br>wR2 = 0.0305
Largest diff. peak/hole /e $Å^{-3}$	0.19/-0.20	0.11/-0.09	0.12/-0.10	0.15/-0.13	0.11/-0.12
Flack parameter	-	0.4(3)	-0.05(14)	0.02(15)	-
Weights	0 0	0.019409<br>0.007011	0.010647 0	0.013851<br>0.00386	0.008584 0
Extinction	-	-	0.386(13)	0.0432(16)	-

Table S4 Refinement parameters of chosen model compounds, Independent Atom Model. 0.8 Å resolution.

Identification code	α-glycine	β-glycine	L-alanine	xylitol	naphthalene
Data/restraints/parameters	619/0/66	630/1/ 66	869/ 0/ 84	1333/0/139	700/0/62
Goodness-of-fit on F^2	1.0648	1.2167	1.127	1.0769	1.091
Final R indexes [I ≥ 2σ (I)]	R1 = 0.0291<br>wR2 = 0.0727	R1 = 0.0 211<br>wR2 = 0.0 536	R1 = 0.0216<br>wR2 = 0.0609	R1 = 0.0196<br>wR2 = 0.0516	R1 = 0.0331<br>wR2 = 0.0900
Final R indexes [all data]	R1 = 0.0328<br>wR2 = 0.0757	R1 = 0.0 213<br>wR2 =0.0 537	R1 = 0.0216<br>wR2 = 0.0610	R1 = 0.0198<br>wR2 = 0.0518	R1 = 0.0336<br>wR2 = 0.0906
Largest diff. peak/hole /e $Å^{-3}$	0.32/-0.30	0.25/-0.18	0.17/-0.16	0.23/-0.17	0.26/-0.21
Flack parameter	-	0.7(3)	0.25(11)	-0.2(3)	-
Weights	0.026011<br>0.186724	0.014247<br>0.063096	0.040800<br>0.029600	0.034745<br>0.086602	0.049600<br>0.091800
Extinction	-	-	0.46(4)	-	-

Table S5 Refinement parameters of chosen model compounds after HAR refinement. 0.8 Å resolution.

Identification code	α-glycine	β-glycine	L-alanine	xylitol	naphthalene
Data/restraints/parameters	619/0/91	630/1/ 91	869/ 0/ 119	1333/0/200	700/0/82
Goodness-of-fit on F^2	1.1088	1.1918	1.1310	0.6595	1.0436
Final R indexes [I ≥ 2σ (I)]	R1 = 0.0160<br>wR2 = 0.0374	R1 = 0.0130<br>wR2 = 0.0393	R1 = 0.0086<br>wR2 = 0.0253	R1 = 0.0065<br>wR2 = 0.0169	R1 = 0.0079<br>wR2 = 0.0202
Final R indexes [all data]	R1 = 0.0194<br>wR2 = 0.0396	R1 = 0.0132<br>wR2 =0.0394	R1 = 0.0086<br>wR2 = 0.0253	R1 = 0.0067<br>wR2 = 0.0170	R1 = 0.0082<br>wR2 = 0.0204
Largest diff. peak/hole /e $Å^{-3}$	0.13/-0.14	0.04/-0.23	0.06/-0.08	0.05/-0.04	0.04/-0.06
Flack parameter	-	0.6(5)	0.3(3)	-0.2(3)	-
Weights	0 0	0.021534<br>0.012372	0 0	0.010889<br>0.011729	0 0
Extinction	-	-	0.454(15)	0.0338(16)	-

Table S6 Refinement parameters of chosen model compounds after TAAM refinement. 0.8 Å resolution.

Identification code	α-glycine	β-glycine	L-alanine	xylitol	naphthalene
Data/restraints/parameters	1033/0/91	630/1/ 91	869/ 0/ 119	1333/0/200	700/0/82
Goodness-of-fit on F^2	1.1330	1.2424	0.9895	0.7033	1.1983
Final R indexes [I ≥ 2σ (I)]	R1 = 0.0 167<br>wR2 = 0.0 422	R1 = 0.0125<br>wR2 = 0.0378	R1 = 0.0076<br>wR2 = 0.0221	R1 = 0.0069<br>wR2 = 0.0179	R1 = 0.0084<br>wR2 = 0.0232
Final R indexes [all data]	R1 = 0.0200<br>wR2 = 0.0400	R1 = 0.0127<br>wR2 =0.0380	R1 = 0.0076<br>wR2 = 0.0222	R1 = 0.0070<br>wR2 = 0.0179	R1 = 0.0087<br>wR2 = 0.0234
Largest diff. peak/hole /e $Å^{-3}$	0.12/-0.16	0.04/-0.26	0.06/-0.07	0.05/-0.05	0.04/-0.06
Flack parameter	-	0.6(5)	0.3(3)	-0.2(3)	-
Weights	0.007959 0	0.019537<br>0.010213	0 0	0.010589<br>0.010985	0 0
Extinction	-	-	0.454(13)	0.0382(18)	-

Table S7 wR2 obtained from IAM, HAR, TAAM models and after NoMoRe, HAR_NoMoRe and TAAM_NoMoRe refinements.

	α-glycine	β-glycine	L-alanine	Xylitol	Naphthalene
max resolution					
IAM	0.0800	0.0678	0.0597	0.0570	0.1002
NoMoRe	0.0881	0.0781	0.0993	0.0589	0.1055
HAR	0.0463	0.0445	0.0353	0.0319	0.0454
HAR_NoMoRe(mo)	0.0568	0.0418	0.0727	0.0503	0.0367
HAR_NoMoRe(mA)	0.0556	0.0398	0.0715	0.0486	0.0362
TAAM	0.0478	0.0426	0.0341	0.0326	0.0305
TAAM_NoMoRe(mo)	0.0560	0.0406	0.0723	0.0484	0.0358
TAAM_NoMoRe(mA)	0.0554	0.0391	0.0713	0.0481	0.0357
0.8 Å resolution					
IAM	0.0757	0.0533	0.0610	0.0518	0.0906
NoMoRe	0.0858	0.0612	0.0833	0.0658	0.1063
HAR	0.0396	0.0394	0.0253	0.0170	0.0204
HAR_NoMoRe(mo)	0.0521	0.0421	0.0503	0.0378	0.0341
HAR_NoMoRe(mA)	0.0503	0.0394	0.0440	0.0302	0.0321
TAAM	0.0422	0.0380	0.0222	0.0179	0.0234
TAAM_NoMoRe(mo)	0.0510	0.0535	0.0459	0.0305	0.0607
TAAM_NoMoRe(mA)	0.0501	0.0500	0.0441	0.0292	0.0580

Table S8 R1 obtained from IAM, HAR, TAAM models and after NoMoRe, HAR_NoMoRe and TAAM_NoMoRe refinements.

	α-glycine	β-glycine	L-alanine	Xylitol	Naphthalene
max resolution					
IAM	0.0416	0.0253	0.0223	0.0294	0.0355
NoMoRe	0.0452	0.0302	0.0339	0.0394	0.0387
HAR	0.0289	0.0169	0.0137	0.0219	0.0196
HAR_NoMoRe(mo)	0.0321	0.0209	0.0237	0.0316	0.0222
HAR_NoMoRe(mA)	0.0320	0.0204	0.0233	0.0311	0.0222
TAAM	0.0294	0.0162	0.0124	0.0220	0.0191
TAAM_NoMoRe(mo)	0.0320	0.0206	0.0235	0.0310	0.0218
TAAM_NoMoRe(mA)	0.0319	0.0201	0.0232	0.0311	0.0219
0.8 Å resolution					
IAM	0.0328	0.0213	0.0216	0.0198	0.0336
NoMoRe	0.0381	0.0257	0.0274	0.0236	0.0406
HAR	0.0194	0.0132	0.0086	0.0067	0.0082
HAR_NoMoRe(mo)	0.0237	0.0212	0.0137	0.0131	0.0109
HAR_NoMoRe(mA)	0.0232	0.0203	0.0132	0.0108	0.0109
TAAM	0.0200	0.0127	0.0076	0.0070	0.0088
TAAM_NoMoRe(mo)	0.0233	0.0240	0.0132	0.0105	0.0267
TAAM_NoMoRe(mA)	0.0229	0.0227	0.0133	0.0104	0.0262

S2. I/σ(I) vs. Resolution plots for IAM of all model compounds

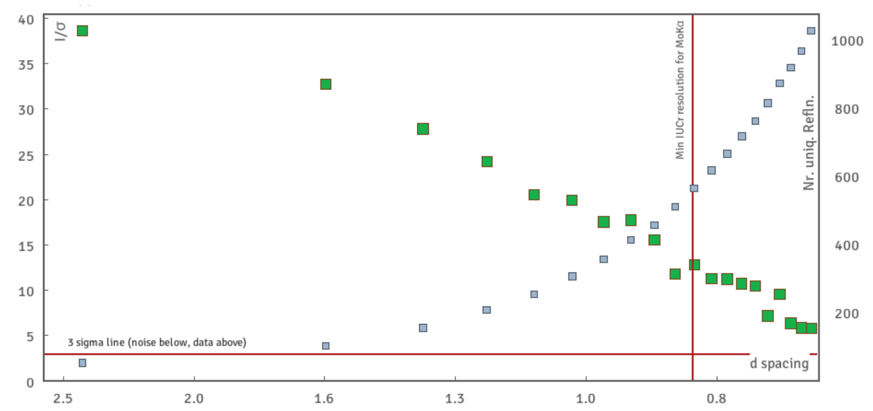

Figure S1 I/σ(I) vs. Resolution plots for α-glycine

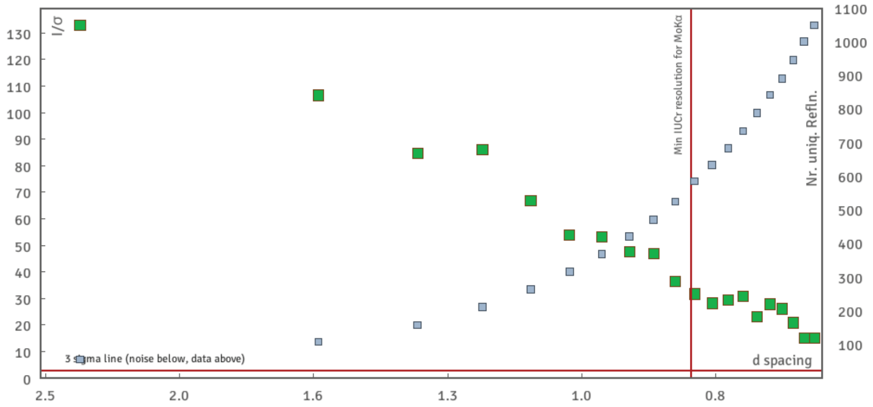

Figure S2 I/σ(I) vs. Resolution plots for β-glycine

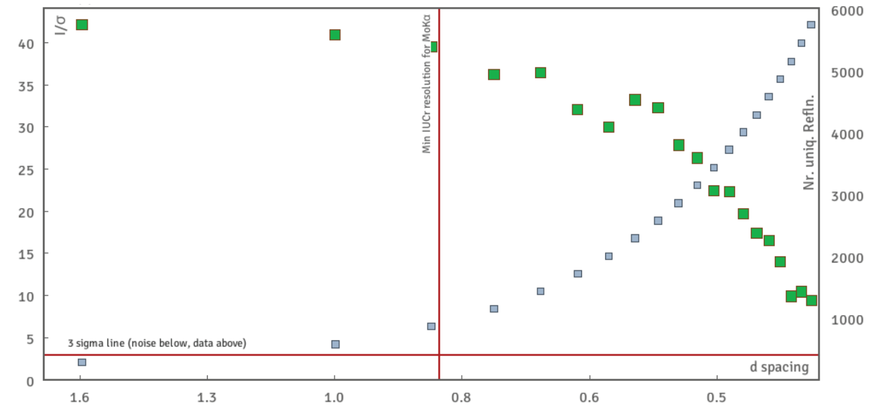

Figure S3 I/σ(I) vs. Resolution plots for L-alanine

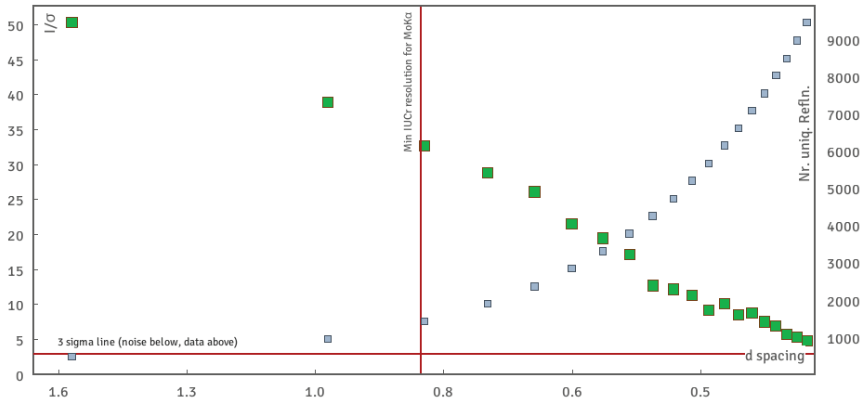

Figure S4 I/σ(I) vs. Resolution plots for Xylitol

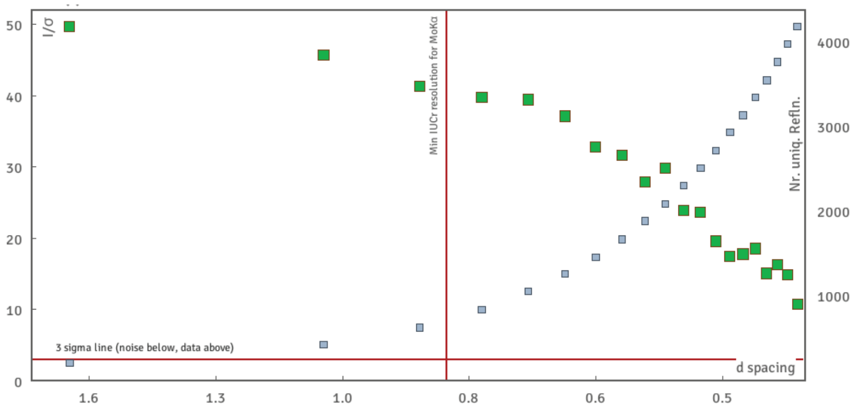

Figure S5 I/σ(I) vs. Resolution plots for Naphthalene

S3. Fractal dimensional plots for σ-glycine

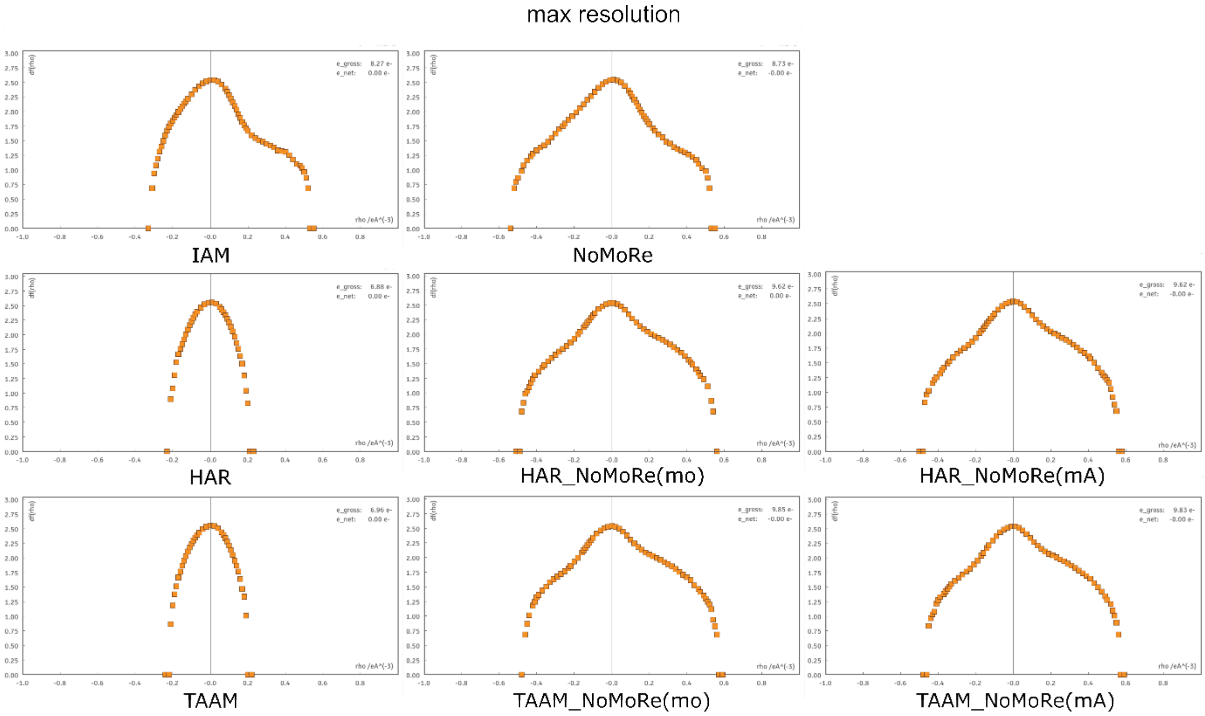

Figure S6 Fractal dimensional plots for α-glycine, for models: IAM, NoMoRe, HAR, HAR_NoMoRe, TAAM and TAAM_NoMoRe. Plots generated for max resolution data.

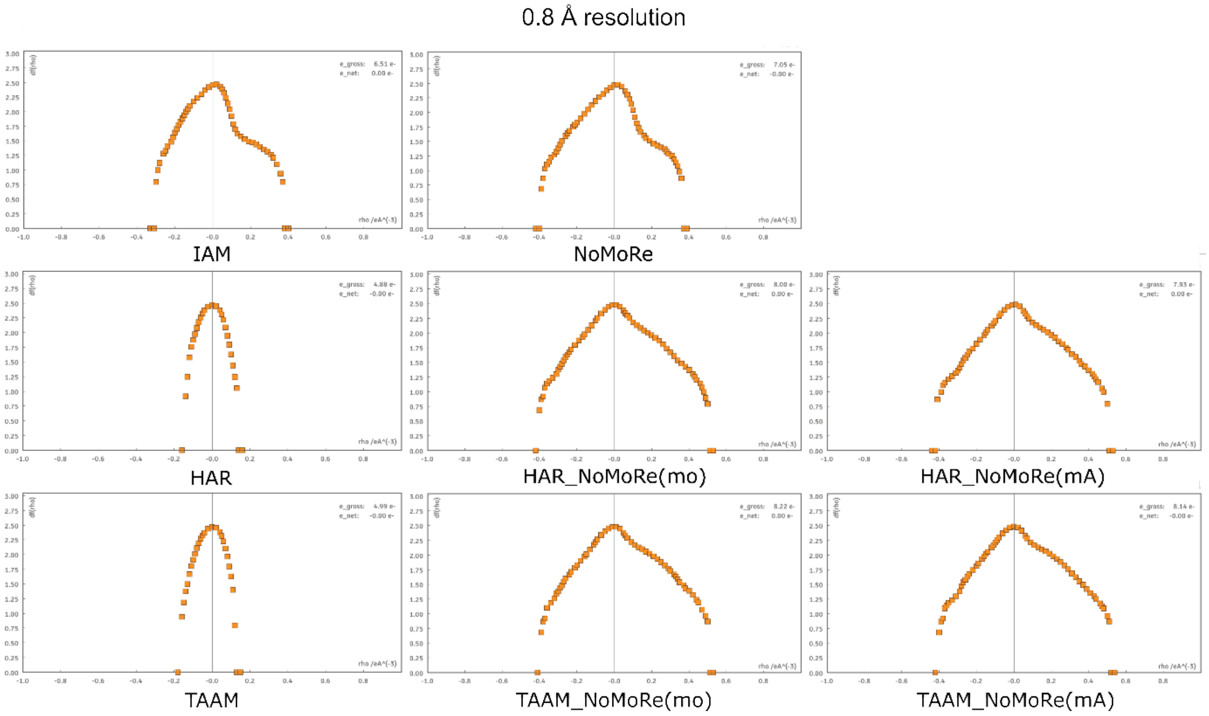

Figure S7 Fractal dimensional plots for α-glycine, for models: IAM, NoMoRe, HAR, HAR_NoMoRe, TAAM and TAAM_NoMoRe. Plots generated for data cut in 0.8 Å.

S4. Residual maps for β-glycine, L-alanine, Xylitol and Naphthalene

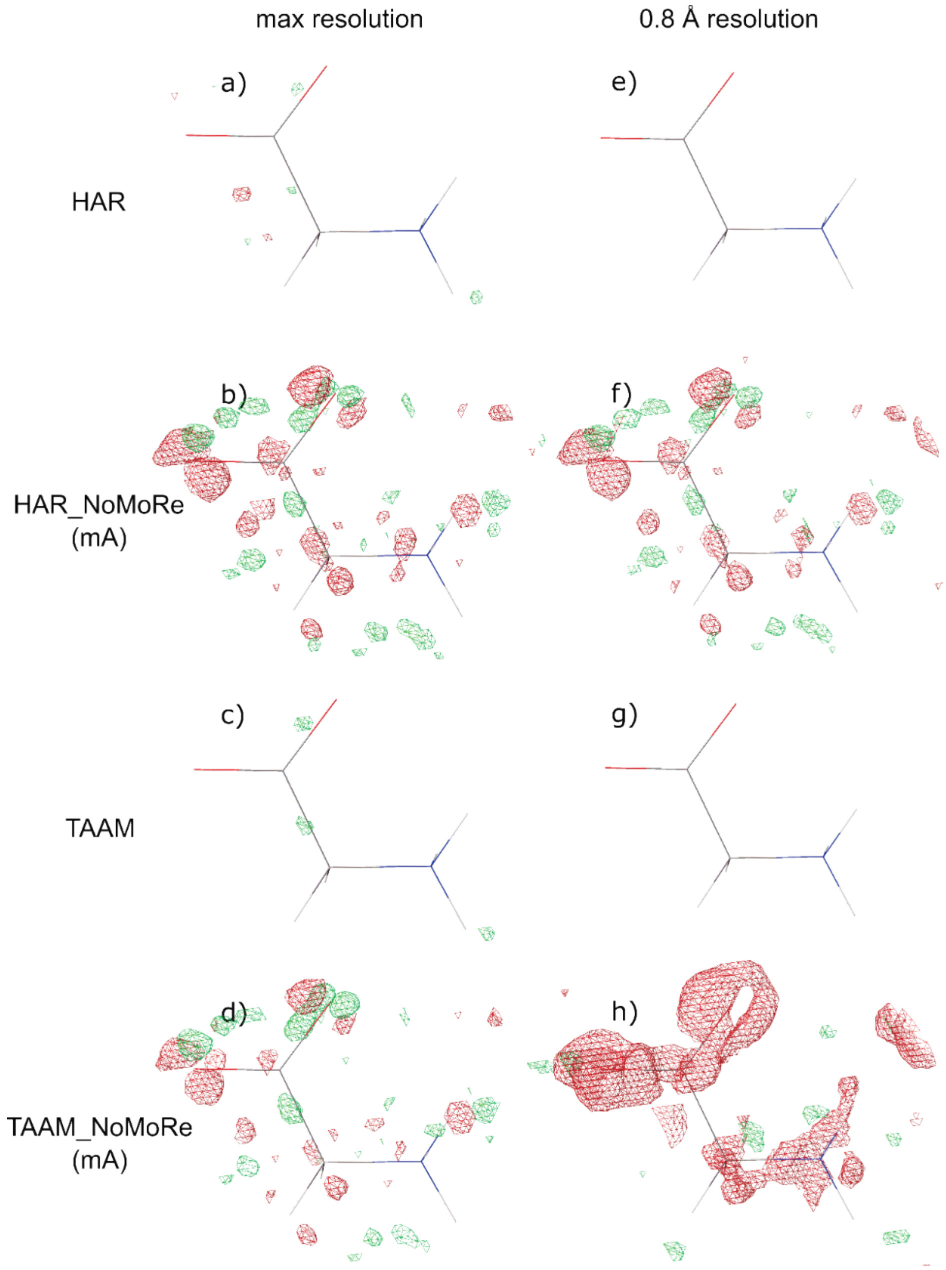

Figure S8 Residual density isosurfaces of β-glycine polymorph for max resolution and resolution cut in 0.8 Å. Maps after HAR, HAR_NoMoRe, TAAM, TAAM_NoMoRe refinement are compared. Isosurface level for 0.10 eÅ^{-1}, green positive, red negative.

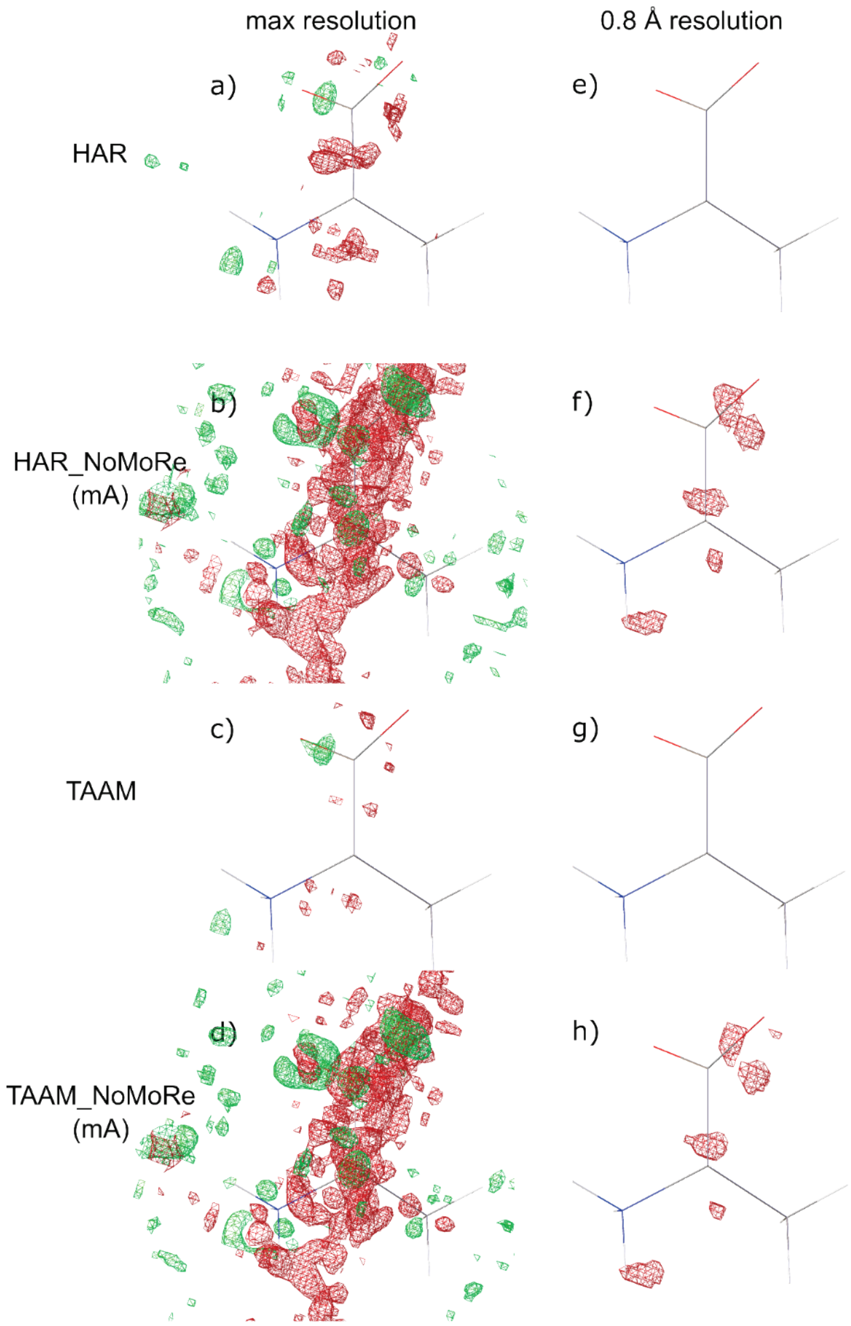

Figure S9 Residual density isosurfaces of L-alanine for max resolution and resolution cut in 0.8 Å. Maps after HAR, HAR_NoMoRe, TAAM, TAAM_NoMoRe refinement are compared. Isosurface level for 0.10 eÅ^{-1}, green positive, red negative.

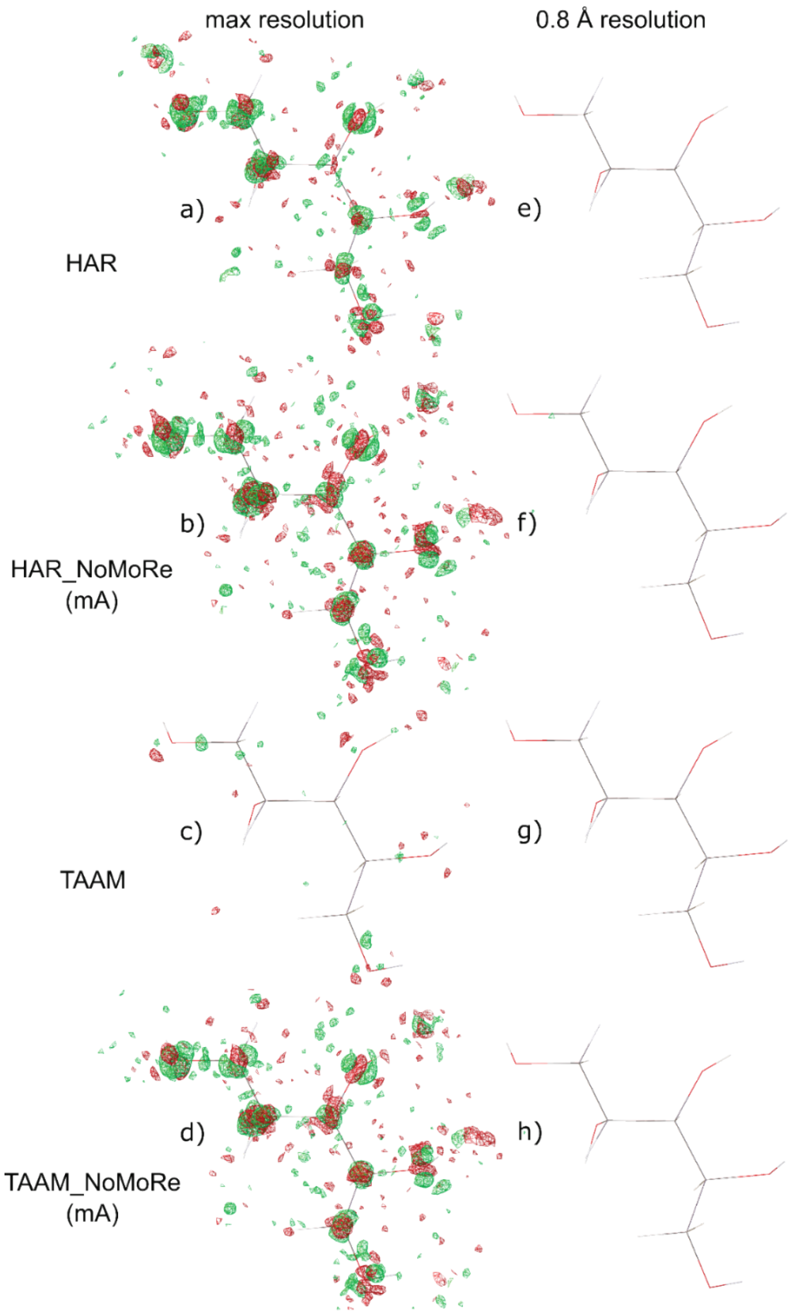

Figure S10 Residual density isosurfaces of xylitol for max resolution and resolution cut in 0.8 Å. Maps after HAR, HAR_NoMoRe, TAAM, TAAM_NoMoRe refinement are compared. Isosurface level for 0.12 eÅ^{-1}, green positive, red negative.

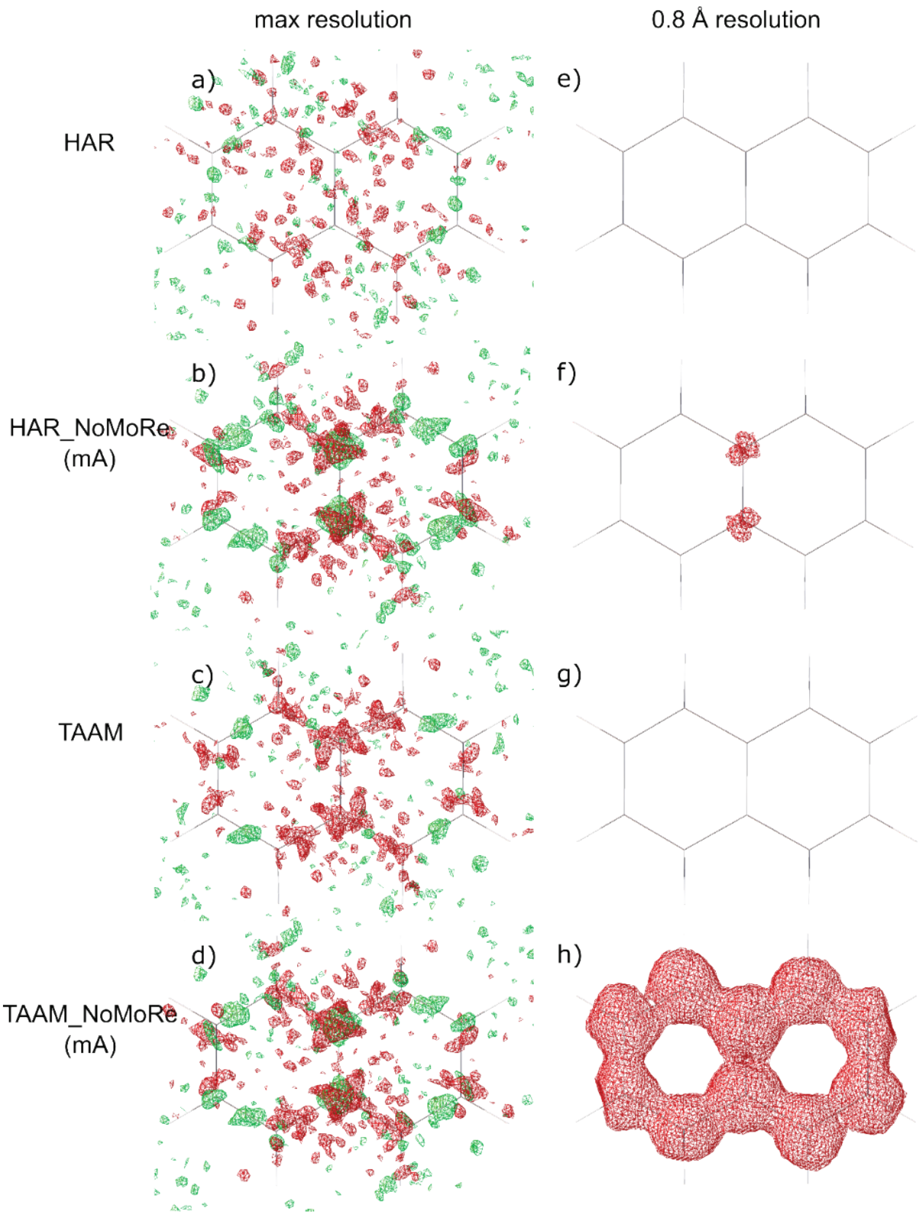

Figure S11 Residual density isosurfaces of naphthalene for max resolution and resolution cut in 0.8 Å. Maps after HAR, HAR_NoMoRe, TAAM, TAAM_NoMoRe refinement are compared. Isosurface level for 0.08 eÅ^{-1}, green positive, red negative.

S5. Geometry and Similarity Index

For bond lengths comparison, we calculated root mean square (d_{RMS}) for H-bonds, according to the equation:

$$d_{RMS} = \sqrt{\frac{\sum_{i=1}^{n} d_i^2}{n}}$$

Where d_i is the difference in length of the same bond of molecule obtained from refinement of neutron and X-ray diffraction data.

To judge the H-angles accuracy, as in the case of the bond lengths, we calculated root mean square (A_{RMS}) for angles with hydrogen atom involved, according to the equation:

$$A_{RMS} = \sqrt{\frac{\sum_{i=1}^{n} A_i^2}{n}}$$

where A_i is a difference in degrees of the same angle of molecule obtained from neutron and X-ray diffraction measurements.

Table S9 Root mean square for X-H bond lengths of structures obtained from HAR/TAAM and all tested models: HAR_NoMoRe(mo, mA) and TAAM_NoMoRe(mo, mA). Results are expressed in Å.

	α-glycine	L-alanine	Xylitol	Naphthalene
		max resolution		
HAR	0.011(8)	0.018(7)	0.017(5)	0.012(5)
HAR_NoMoRe(mo)	0.011(8)	0.018(7)	0.017(5)	0.012(5)
HAR_NoMoRe(mA)	0.013(6)	0.046(7)	0.040(5)	0.018(5)
TAAM	0.012(10)	0.037(7)	0.042(5)	0.015(5)
TAAM_NoMoRe(mo)	0.012(10)	0.037(7)	0.042(5)	0.015(5)
TAAM_NoMoRe(mA)	0.011(6)	0.047(5)	0.040(5)	0.018(5)
		0.8 Å resolution		
HAR	0.011(7)	0.015(6)	0.025(7)	0.013(5)
HAR_NoMoRe(mo)	0.011(7)	0.015(6)	0.025(7)	0.013(5)
HAR_NoMoRe(mA)	0.014(5)	0.043(5)	0.046(5)	0.017(6)
TAAM	0.011(12)	0.031(7)	0.042(6)	0.017(5)
TAAM_NoMore(mo)	0.011(12)	0.031(7)	0.042(6)	0.017(5)
TAAM_NoMore(mA)	0.013(7)	0.043(6)	0.044(5)	0.016(5)

Table S10 Root mean square for H-angles of structures obtained from HAR/TAAM and all tested models: HAR_NoMoRe(mo, mA) and TAAM_NoMoRe(mo, mA). Data are expressed in °.

	α-glycine	L-alanine	Xylitol	Naphthalene
max resolution				
HAR	1.4(5)	0.9(5)	0.5(2)	0.3(3)
HAR_NoMoRe(mo)	1.4(5)	0.9(5)	0.5(2)	0.3(3)
HAR_NoMoRe(mA)	1.4(4)	1.2(4)	0.8(3)	0.3(2)
TAAM	1.2(6)	0.5(4)	0.6(3)	0.3(3)
TAAM_NoMoRe(mo)	1.2(6)	0.5(4)	0.6(3)	0.3(3)
TAAM_NoMoRe(mA)	1.3(4)	1.1(3)	0.8(3)	0.3(2)
0.8 Å resolution				
HAR	1.4(5)	0.9(4)	0.4(3)	0.3(3)
HAR_NoMoRe(mo)	1.4(5)	0.9(4)	0.4(3)	0.3(3)
HAR_NoMoRe(mA)	1.3(4)	1.3(4)	0.8(3)	0.3(2)
TAAM	1.2(6)	0.9(4)	0.6(3)	0.3(3)
TAAM_NoMoRe(mo)	1.2(6)	0.9(4)	0.6(3)	0.3(3)
TAAM_NoMoRe(mA)	1.3(4)	1.3(4)	0.8(3)	0.4(2)

Table S11 Similarity index for non-hydrogen ($\bar{S}_{nonH}$) and hydrogen ($\bar{S}_H$) atoms ADPs using back-end library hikari(Tchoń & Makal, 2021)

	α-glycine<br>$\bar{S}_{nonH}/\bar{S}_H$	L-alanine<br>$\bar{S}_{nonH}/\bar{S}_H$	Xylitol<br>$\bar{S}_{nonH}/\bar{S}_H$	Naphthalene<br>$\bar{S}_{nonH}/\bar{S}_H$
max resolution				
NoMoRe	0.48/0.17	0.26/0.24	0.28/0.34	0.44/0.25
HAR	0.15/3.50	0.18/2.56	0.14/1.90	0.44/0.31
HAR_NoMoRe(mo)	0.11/0.08	0.28/0.26	0.29/0.37	0.42/0.23
HAR_NoMoRe(mA)	0.12/0.08	0.26/0.25	0.28/0.37	0.42/0.23
TAAM	0.13/3.96	0.17/3.20	0.16/4.44	0.43/0.90
TAAM_NoMoRe(mo)	0.13/0.08	0.25/0.24	0.29/0.38	0.42/0.23
TAAM_NoMoRe(mA)	0.13/0.08	0.27/0.26	0.29/0.37	0.42/0.22
0.8 Å resolution				
NoMoRe	0.67/0.20	0.23/0.23	0.30/0.44	1.01/0.73
HAR	0.24/4.75	0.24/2.88	0.16/1.58	0.44/0.46
HAR_NoMoRe(mo)	0.13/0.07	0.26/0.27	0.37/0.36	0.40/0.24
HAR_NoMoRe(mA)	0.14/0.08	0.26/0.27	0.34/0.36	0.39/0.24
TAAM	0.22/4.33	0.20/2.27	0.22/5.41	0.46/1.03
TAAM_NoMoRe(mo)	0.12/0.07	0.28/0.28	0.35/0.37	0.38/0.30
TAAM_NoMoRe(mA)	0.14/0.07	0.29/0.28	0.35/0.37	0.38/0.30

Table S12 Equivalent isotropic displacement parameters for hydrogen atoms for all tested models of chosen compounds. The values are presented in Å^2.

	α-glycine	L-alanine	Xylitol	Naphthalene
	Ueq_H	Ueq_H	Ueq_H	Ueq_H
neutron	4.1498	2.9107	1.5363	2.1834
max resolution				
NoMoRe	4.5176	3.0606	1.5298	2.4313
HAR	4.1972	3.2634	2.2790	2.2790
HAR_NoMoRe(mo)	4.3209	3.0531	1.5157	2.3875
HAR_NoMoRe(mA)	4.3359	3.0608	1.5175	2.3905
TAAM	5.2576	3.2255	1.8551	1.8551
TAAM_NoMoRe(mo)	4.2766	3.0733	1.5182	2.3961
TAAM_NoMoRe(mA)	4.2569	3.0598	1.5214	2.3936
0.8 Å resolution				
NoMoRe	4.8720	3.3312	1.6277	2.8653
HAR	4.3660	1.5888	2.2632	5.1909
HAR_NoMoRe(mo)	4.3171	3.1485	1.4952	2.4625
HAR_NoMoRe(mA)	4.3431	3.1484	1.4938	2.4614
TAAM	5.4265	3.5597	1.7813	2.4935
TAAM_NoMoRe(mo)	4.2602	3.1412	1.5057	2.3961
TAAM_NoMoRe(mA)	4.2599	3.1266	1.5060	2.3936

Table S13 .Equivalent isotropic displacement parameters for non-hydrogen atoms for all tested models of chosen compounds. The values are presented in Å2.

	α-glycine	L-alanine	Xylitol	Naphthalene
	Ueq_{nonH}	Ueq_{nonH}	Ueq_{nonH}	Ueq_{nonH}
neutron	1.5531	1.1310	0.5567	1.0674
max resolution				
NoMoRe	2.1185	1.4751	0.6842	1.1362
HAR	1.8189	1.4908	0.6614	1.1174
HAR_NoMoRe(mo)	1.8257	1.4569	0.6691	1.1095
HAR_NoMoRe(mA)	1.8265	1.4565	0.6694	2.3875
TAAM	1.7863	1.4978	0.6659	1.1293
TAAM_NoMoRe(mo)	1.7592	1.4649	0.6695	1.1144
TAAM_NoMoRe(mA)	1.7606	1.4567	0.6749	2.3961
0.8 Å resolution				
NoMoRe	2.3992	1.7349	0.7809	1.4641
HAR	1.8089	1.5888	0.6428	2.6385
HAR_NoMoRe(mo)	1.8241	1.5502	0.6420	1.1660

HAR_NoMoRe(mA)	1.8299	1.5486	0.6422	1.1641
TAAM	1.8001	1.5563	0.6566	1.1668
TAAM_NoMoRe(mo)	1.7650	1.5442	0.6544	1.3845
TAAM_NoMoRe(mA)	1.7565	1.5362	0.6544	1.3811

S6. Evaluation of heat capacity

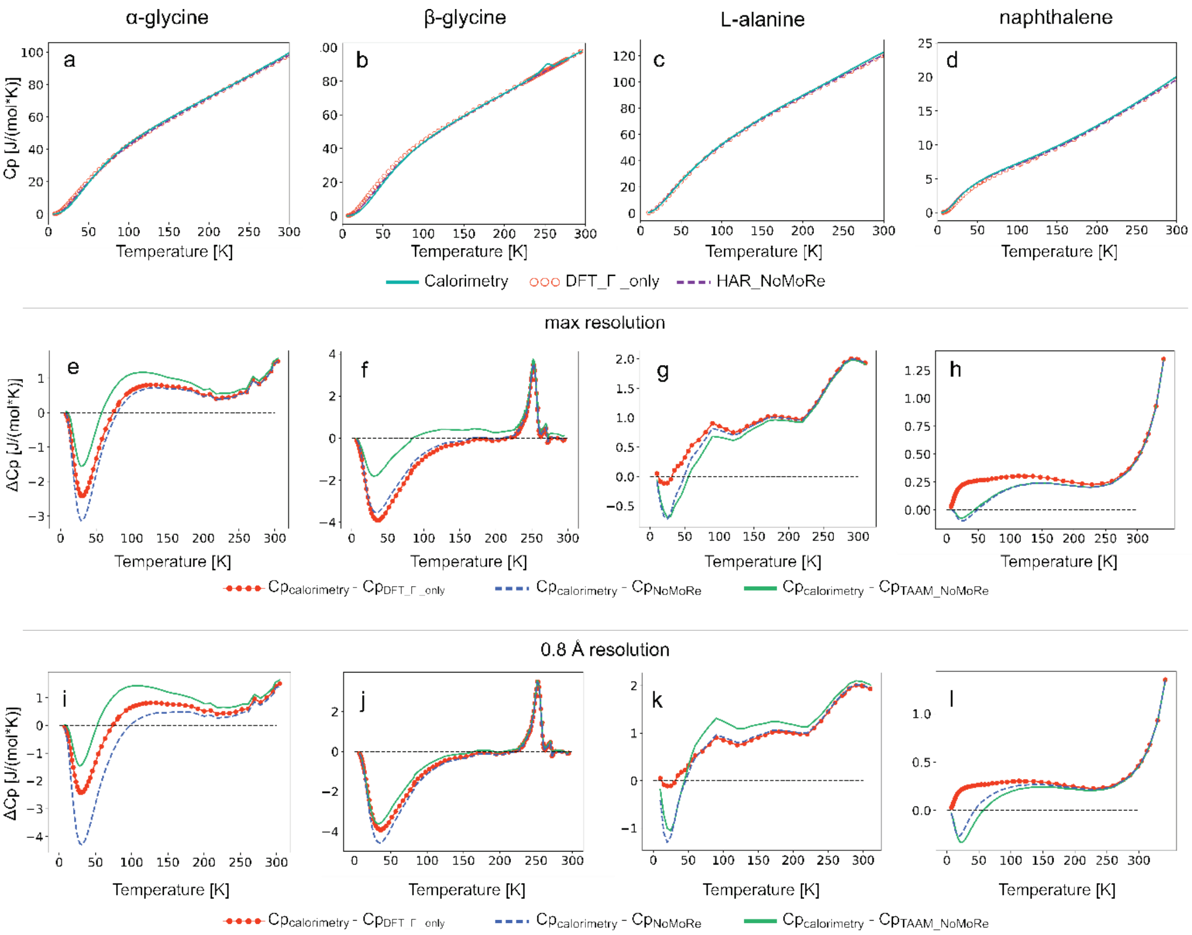

Figure S12(a–d) Heat capacity for five compounds obtained from calorimetry (green solid line), DFT Γ-point calculations with acoustic mode frequencies of 50 cm^{-1} (orange circles), and TAAM_NoMoRe (violet dashed line). (e–h) Difference between heat capacity from calorimetry and DFT Γ-point calculations with acoustic mode frequencies of 50 cm^{-1} (orange dots), TAAM_NoMoRe (solid green line) and NoMoRe (dashed blue line). (i-l) Same as (e-h), but for data cut-off in 0.8 Å resolution. Heat capacity was computed only for temperatures for which the calorimetric data were available. Plots are generated for: (a), (e) and (i) α-glycine, (b), (f) and (j) β-glycine, (c), (g) and (k) L-alanine, (d), (h) and (l) naphthalene.

S7. Refined frequencies for given normal modes

Table S14 Refined frequencies for different models of α -glycine. Modes that were not refined are greyed out.

	DFT	HAR_NoMoRe		TAAM_NoMoRe	
		mo	mA	mo	mA
max resolution					
**1**	-6.301	49.382(30)	53.019(51)	59.322(392)	55.599(60)
**2**	-5.632	52.766(60)	53.014(51)	59.034(69)	57.195(57)
**3**	-5.537	108.601(269)	93.147(318)	114.632(799)	94.096(234)
**4**	53.027	65.142(56)	61.684(38)	58.411(116)	67.127(67)
**5**	61.224	78.856(127)	70.386(76)	72.752(78)	71.765(66)
**6**	67.120	103.853(190)	101.445(151)	85.874(1139)	97.000(201)
**7**	82.709	71.507(58)	89.434(164)	100.177(165)	80.750(75)
**8**	84.952	84.950	84.950	84.950	84.950
**9**	92.611	66.723(63)	65.398(59)	62.664(46)	66.877(56)
**10**	103.061	200.960(262)	144.700(132)	124.468(232)	183.661(517)
**11**	123.508	123.510	123.510	123.510	123.510
**12**	127.854	127.850	127.850	127.850	127.850
**13**	152.259	59.730(16)	60.330(21)	59.333(18)	57.930(18)
0.8 Å resolution					
**1**		47.515(51)	49.759(47)	54.420(57)	50.171(91)
**2**		53.234(56)	55.207(61)	62.741(130)	59.615(82)
**3**		110.862(395)	93.860(284)	107.514(375)	108.773(436)
**4**		69.605(105)	61.887(74)	65.689(85)	69.743(108)
**5**		84.014(112)	80.570(80)	78.374(89)	80.563(237)
**6**		104.924(227)	100.297(220)	90.596(226)	104.218(244)
**7**		63.978(59)	83.347(138)	73.124(108)	69.721(70)
**8**		84.950	84.950	84.950	84.950
**9**		64.925(70)	64.062(62)	59.995(61)	64.129(53)
**10**		169.298(322)	132.389(245)	153.216(211)	204.410(357)
**11**		123.510	123.510	123.510	123.510
**12**		127.850	127.850	127.850	127.850
**13**		61.755(26)	59.541(20)	60.373(22)	59.261(22)

Table S15 Refined frequencies for different models of β-glycine.

	DFT	HAR_NoMoRe		TAAM_NoMoRe	
		mo	mA	mo	mA
max resolution					
**1**	-6.3868	62.592(15)	61.067(11)	64.822(13)	61.898(12)
**2**	-5.4484	44.912(3)	44.908(3)	45.486(3)	45.542(3)
**3**	-5.3053	142.088(171)	116.885(68)	146.794(81)	130.154(83)
**4**	66.0906	79.684(21)	83.682(18)	78.533(23)	84.911(29)
**5**	91.1993	62.283(7)	65.602(6)	63.273(7)	64.849(8)

0.8 Å resolution				
**1**	62.359(11)	60.012(11)	56.321(11)	56.054(13)
**2**	44.908(3)	44.827(3)	40.353(2)	40.421(2)
**3**	133.422(108)	115.163(92)	92.768(45)	94.833(65)
**4**	80.546(15)	87.379(22)	69.123(16)	69.164(18)
**5**	63.120(6)	65.763(9)	58.537(7)	58.886(9)

Table S16 Refined frequencies for different models of L-alanine. Refined modes are bolded.

	DFT	HAR_NoMoRe		TAAM_NoMoRe	
		mo	mA	mo	mA
max resolution					
**1**	-7.0959	40.464(1)	40.079(1)	40.181(1)	40.205(1)
**2**	-6.2765	45.620(5)	46.298(5)	44.890(4)	45.652(5)
**3**	-5.7614	76.961(44)	78.548(59)	75.305(26)	74.802(34)
**4**	47.7135	44.140(5)	41.791(5)	42.387(5)	43.595(5)
**5**	60.6508	58.115(9)	55.532(7)	55.528(8)	59.471(9)
**6**	77.7873	124.900(71)	117.994(85)	124.074(35)	107.600(44)
7	96.0502	96.050	96.050	96.050	96.050
**8**	99.0953	77.463(5)	78.317(5)	75.860(5)	77.172(4)
**9**	99.9186	77.746(7)	88.396(10)	87.175(11)	84.988(8)
10	105.5465	105.550	105.550	105.550	105.550
11	108.5907	108.590	108.590	108.590	108.590
12	112.3233	112.320	112.320	112.320	112.320
**13**	112.7423	73.180(5)	85.086(7)	93.926(12)	74.480(6)
0.8 Å resolution					
**1**		37.008(4)	37.758(4)	37.867(4)	37.910(4)
**2**		42.687(12)	41.290(16)	39.474(11)	44.123(19)
**3**		64.627(74)	60.692(43)	60.833(81)	68.250(93)
**4**		42.394(17)	44.203(19)	45.776(12)	40.988(19)
**5**		57.662(34)	60.563(33)	63.187(19)	53.677(31)
**6**		108.930(156)	120.984(70)	131.870(145)	211.605(351)
7		96.050	96.050	96.050	96.050
**8**		78.334(11)	76.051(13)	75.668(12)	77.464(14)
**9**		89.144(28)	80.954(21)	76.370(27)	78.468(20)
10		105.550	105.550	105.550	105.550
11		108.590	108.590	108.590	108.590
12		112.320	112.320	112.320	112.320
**13**		69.825(13)	69.417(13)	74.731(20)	74.057(18)

Table S17 Refined frequencies for different models of xylitol.

	DFT	HAR_NoMoRe		TAAM_NoMoRe	
		mo	mA	mo	mA
		max resolution			
**1**	-6.4828	32.817(2)	33.352(3)	33.663(2)	32.740(3)
**2**	-5.8015	28.019(2)	28.036(2)	27.872(2)	27.525(2)
**3**	-4.6422	58.799(32)	69.215(40)	74.265(30)	63.643(30)
**4**	49.0212	47.523(9)	47.529(9)	48.124(9)	50.259(11)
**5**	55.3224	41.052(6)	39.335(5)	38.967(7)	40.639(6)
**6**	60.6019	66.133(19)	63.593(16)	60.472(14)	65.960(18)
**7**	67.6958	56.965(22)	70.843(27)	73.799(19)	58.446(16)
**8**	91.1207	72.365(12)	54.467(8)	51.581(10)	63.203(9)
		0.8 Å resolution			
**1**		33.142(8)	33.694(7)	32.266(6)	32.160(6)
**2**		28.160(4)	28.811(4)	28.101(4)	28.227(5)
**3**		53.140(45)	56.599(71)	59.793(67)	55.042(66)
**4**		50.760(23)	47.386(27)	48.143(22)	47.690(25)
**5**		44.957(21)	43.061(20)	40.145(16)	41.170(20)
**6**		63.278(41)	63.379(39)	66.810(43)	67.615(33)
**7**		66.809(58)	87.230(38)	66.005(47)	70.292(37)
**8**		79.262(47)	62.585(58)	79.702(47)	77.681(37)

Table S18 Refined frequencies for different models of naphthalene.

	DFT	HAR_NoMoRe		TAAM_NoMoRe	
		mo	mA	mo	mA
		max resolution			
**1**	-5.4969	44.373(7)	42.155(9)	42.887(5)	40.625(7)
**2**	-5.2028	41.454(2)	41.287(3)	40.866(1)	40.852(3)
**3**	-4.9700	37.243(1)	37.186(2)	36.857(1)	36.763(2)
**4**	48.1538	57.032(11)	57.647(8)	55.890(6)	55.462(8)
**5**	52.3898	51.021(4)	51.123(5)	51.791(3)	51.996(6)
**6**	59.8897	50.093(6)	49.485(4)	50.490(4)	50.949(5)
**7**	74.8192	60.709(11)	68.233(23)	64.793(12)	75.626(33)
**8**	81.9605	66.591(7)	67.921(10)	64.633(5)	63.641(4)
**9**	86.5941	92.572(16)	89.495(19)	99.558(17)	102.029(13)
**10**	105.4188	106.869(16)	105.433(19)	110.741(16)	116.458(18)
**11**	129.3164	100.619(8)	94.355(4)	108.081(10)	113.276(13)
**12**	142.2359	126.565(13)	156.273(29)	119.394(16)	107.400(12)
		0.8 Å resolution			
**1**		40.067(17)	39.243(26)	32.901(8)	35.966(25)
**2**		41.070(6)	41.578(7)	35.088(6)	35.098(5)
**3**		35.578(3)	35.943(4)	31.480(4)	31.332(3)
**4**		58.265(22)	56.019(15)	57.025(13)	57.797(15)
**5**		48.858(10)	48.215(11)	46.593(13)	46.581(10)
**6**		47.109(10)	48.394(9)	48.045(7)	47.400(7)

**7**	71.579(64)	76.739(126)	80.562(75)	57.375(70)
**8**	69.691(15)	65.318(16)	66.777(11)	68.323(15)
**9**	88.578(28)	101.545(52)	96.915(38)	90.188(30)
**10**	148.726(96)	122.636(48)	105.287(55)	122.724(77)
**11**	100.304(14)	113.792(30)	97.789(17)	93.752(13)
**12**	136.877(45)	115.932(31)	135.293(32)	149.799(54)

S8. Comparison of refinements with different weighting schemes and extinction parameter

For all models preceding AAM_NoMoRe refinement, weights (WGHT) were considered, and in the case of L-alanine and xylitol, extinction (EXTI) was also taken into account. However, NoMoRe refinement streamlines the weighting scheme to a simple 'one over sigma' approach. This means that each data point is weighted inversely proportional to its uncertainty (sigma), simplifying the refinement process while still accounting for the reliability of each measurement. Therefore, we opted to prepare all starting models without taking WGHT and EXTI under consideration during refinement and conducted AAM_NoMoRe refinements. In case of refinement without EXTI we present only L-alanine and xylitol due to the varied contributions of extinction parameter to their refinements (e.g., HAR model: 0.37 for the first compound and 0.04 for the second). Data for all models are present below.

S8.1. Refinement results with 1/sigma weighting scheme

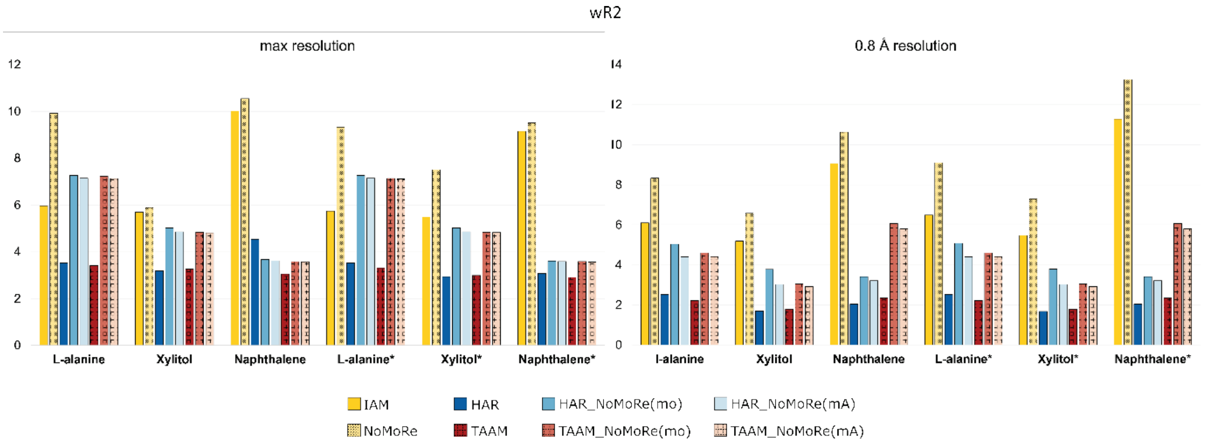

Figure S13 Comparison of wR2 obtained from IAM, HAR and TAAM models and after NoMoRe and HAR/TAAM_NoMoRe refinements for L-alanine, xylitol and naphthalene. Data that do not include weights during refinement are marked with an asterisk. Left - data for max resolution, right - cutoff data in 0.8 Å resolution.

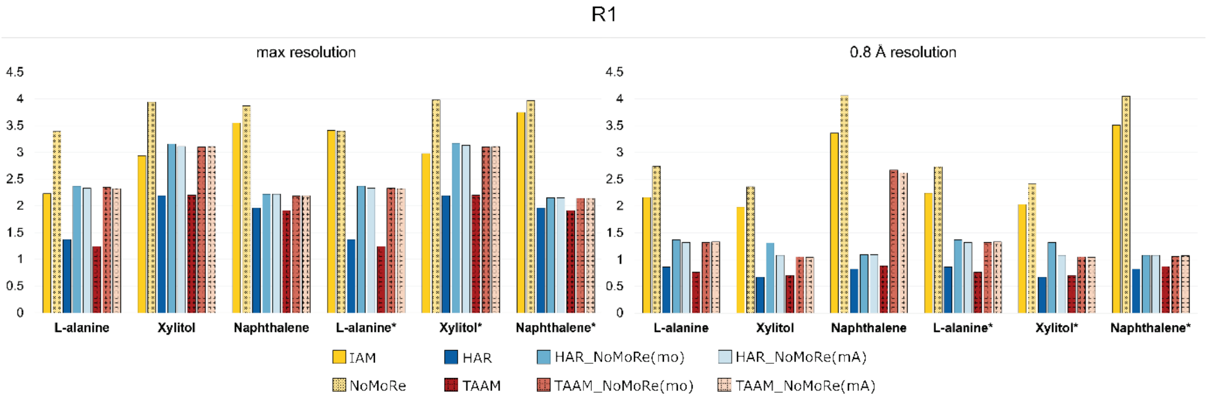

Figure S14 Comparison of R1 obtained from IAM, HAR and TAAM models and after NoMoRe and HAR/TAAM_NoMoRe refinements for L-alanine, xylitol and naphthalene. Data that do include weights 1/sigma during refinement are marked with an asterisk. Left - data for max resolution, right - cutoff data in 0.8 Å resolution.

Figure S13 shows wR2 data for selected compounds at max resolution and 0.8 Å cut-off, with and without weights and extinction during refinement (Table S25). For max resolution data, naphthalene shows the most significant weighting scheme impact. NoMoRe uses a simplified 'one over sigma' weighting approach. Differences range from 0.1 to 1.46 pp for AAM models and 0.01 to 1.03 pp for AAM_NoMoRe. For L-alanine, HAR refinement set weights to 0 0, resulting in no wR2 change for HAR and normal mode refinements. For xylitol, AAM models showed ~0.25 pp lower wR2, while AAM_NoMoRe wR2 remained unchanged with or without WGHT. The same holds for 0.8 Å cut-off data. The weight parameter impact is more pronounced in cut-off data for IAM and NoMoRe, with higher wR2 values than weighted data. Differences are 0.29 – 2.22 pp for IAM and 0.72 – 2.63 pp for NoMoRe. In AAM and normal mode refinements for naphthalene and L-alanine, weights automatically

set to 0 0. As previous, R1 values comparison was also conducted and its value changes corresponds with changes of the wR2 values (see Fig. S14 and Table S26).

Figures S14 and S15 show that WGHT has a negligible influence on molecular geometry, with dRMS differences not exceeding 0.007 Å and A_{RMS} changes only noticeable in the TAAM_NoMoRe(mo) model for naphthalene, likely due to rounding (Tables S27 and S28).

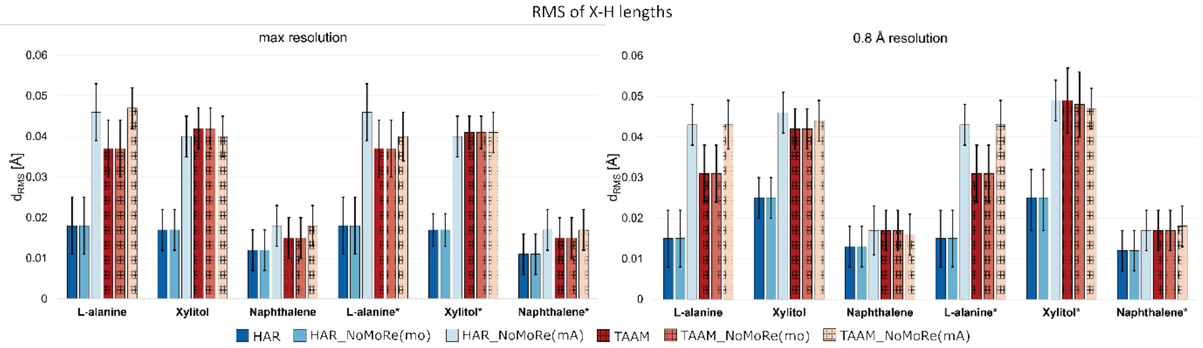

Figure S15 Comparison of root mean square for H-bonds of structures obtained from HAR/TAAM and all tested models: HAR_NoMoRe and TAAM_NoMoRe. Data that do not include weights are marked with an asterisk. Left – max resolution, right – 0.8 Å resolution.

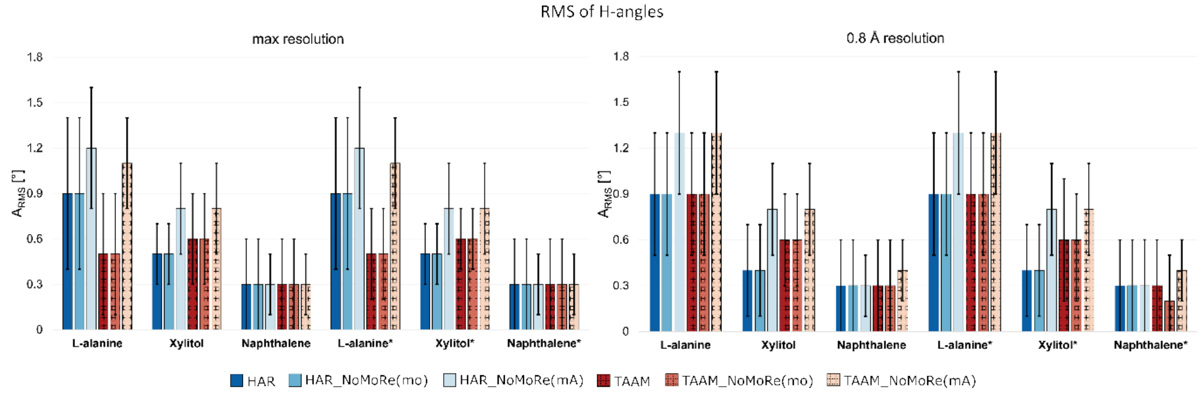

Figure S16 Comparison of root mean square for H-angles of structures obtained from HAR/TAAM and all tested models: HAR_NoMoRe and TAAM_NoMoRe. Data that do not include weights are marked with an asterisk. Left – max resolution, right – 0.8 Å resolution.

Figure S17 compares the similarity index for hydrogen atoms with and without considering WGHT during refinement (all data can be found in Table S9) . The inclusion of weights during refinement significantly impacts the $\bar{S}_H$ value for the NoMoRe model in naphthalene (cut-off resolution data), where this value is almost three times higher than for the maximum resolution data. However, it still does not exceed a value of 1.

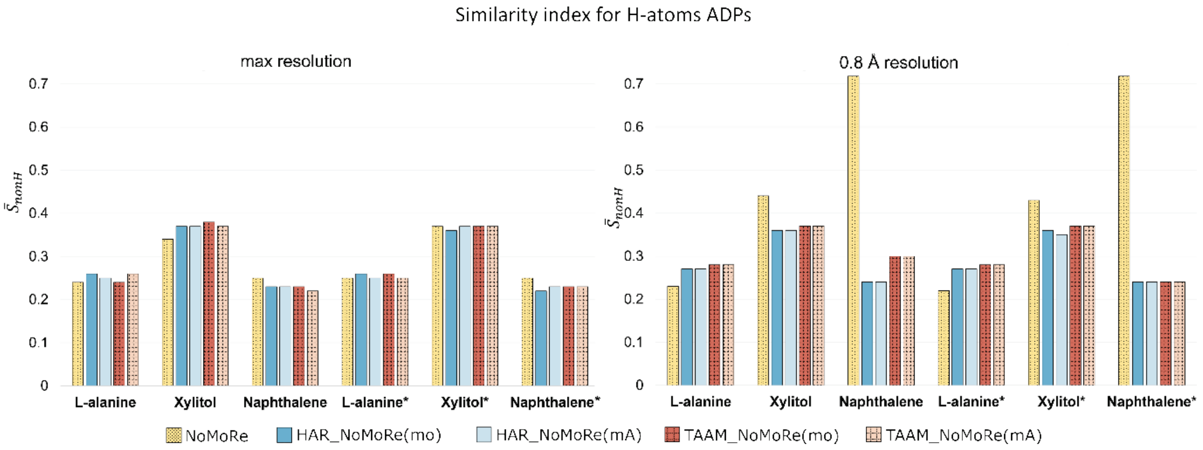

Figure S17 Comparison of similarity indexes of H-atom ADPs ($\bar{S}_H$) of L-alanine, xylitol and naphthalene modelled by all tested models: NoMoRe, HAR_NoMoRe and TAAM_NoMoRe. Data that do not include weights are marked with an asterisk. Left – max resolution, right – 0.8 Å resolution.

S8.1.1. Crystallographic data and refinement parameters

Table S19 Refinement parameters of chosen model compounds when weights were not take into account during refinement. Max resolution.

Identification code	α-glycine	β-glycine	L-alanine	xylitol	naphthalene
Goodness-of-fit on F^2	1.963	3.9341	2.0610	1.2710	3.1975
Final R indexes [I ≥ 2σ (I)]	R1 = 0.0357<br>wR2 = 0.0859	R1 = 0.0264<br>wR2 = 0.0597	R1 = 0.0215<br>wR2 = 0.573	R1 = 0.0239<br>wR2 = 0.0540	R1 = 0.0320<br>wR2 = 0.0912
Final R indexes [all data]	R1 = 0.0437<br>wR2 = 0.0888	R1 = 0.0269<br>wR2 =0.0598	R1 = 0.0223<br>wR2 = 0.574	R1 = 0.0298<br>wR2 = 0.0549	R1 = 0.0320<br>wR2 = 0.0915
Largest diff. peak/hole /e $Å^{-3}$	0.44/-0.35	0.37/-0.26	0.27/-0.24	0.34/-0.24	0.55/-0.20

Table S20 Refinement parameters of chosen model compounds after HAR refinement. Max resolution.

Identification code	α-glycine	β-glycine	L-alanine	xylitol	naphthalene
Data/restraints/parameters	1033/0/91	1046/1/91	3546/ 0/ 119	9942/0/200	4368/0/82
Goodness-of-fit on F^2	1.0385	2.1734	1.2738	0.6817	1.0779
Final R indexes [I ≥ 2σ (I)]	R1 = 0.0214<br>wR2 = 0.0432	R1 = 0.0166<br>wR2 = 0.0326	R1 = 0.0129<br>wR2 = 0.0351	R1 = 0.0160<br>wR2 = 0.0277	R1 = 0.0140<br>wR2 = 0.0297
Final R indexes [all data]	R1 = 0.0289<br>wR2 = 0.0464	R1 = 0.0170<br>wR2 =0.0326	R1 = 0.0137<br>wR2 = 0.0353	R1 = 0.0219<br>wR2 = 0.0294	R1 = 0.0196<br>wR2 = 0.0308
Largest diff. peak/hole /e $Å^{-3}$	0.18/-0.19	0.11/-0.10	0.14/-0.13	0.16/-0.14	0.13/-0.10

Table S21 Refinement parameters of chosen model compounds after TAAM refinement. Max resolution.

Identification code	α-glycine	β-glycine	L-alanine	xylitol	naphthalene
Data/restraints/parameters	1033/0/91	1046/1/91	3546/ 0/ 119	9942/0/200	4368/0/82

Goodness-of-fit on F^2	1.0711	2.0615	1.1942	0.6934	1.0194
Final R indexes [I ≥ 2σ (I)]	R1 = 0.0219<br>wR2 = 0.0448	R1 = 0.0158<br>wR2 = 0.0309	R1 = 0.0117<br>wR2 = 0.0329	R1 = 0.0161<br>wR2 = 0.0282	R1 = 0.0135<br>wR2 = 0.0280
Final R indexes [all data]	R1 = 0.0294<br>wR2 = 0.0478	R1 = 0.0161<br>wR2 =0.0309	R1 = 0.0124<br>wR2 = 0.0331	R1 = 0.0220<br>wR2 = 0.0299	R1 = 0.0191<br>wR2 = 0.0291
Largest diff. peak/hole /e Å^{-3}	0.19/-0.20	0.13/-0.09	0.12/-0.10	0.15/-0.13	0.11/-0.12

Table S22 Refinement parameters of chosen model compounds when weights were not take into account. 0.8 Å resolution.

Identification code	α-glycine	β-glycine	L-alanine	xylitol	naphthalene
Data/restraints/parameters	619/0/66	630/1/66	869/ 0/ 84	1333/0/140	700/0/62
Goodness-of-fit on F^2	2.3103	4.5242	2.836	2.5620	5.687
Final R indexes [I ≥ 2σ (I)]	R1 = 0.0310<br>wR2 = 0.0822	R1 = 0.0220<br>wR2 = 0.0546	R1 = 0.0224<br>wR2 = 0.0649	R1 = 0.0202<br>wR2 = 0.0547	R1 = 0.0345<br>wR2 = 0.1124
Final R indexes [all data]	R1 = 0.0349<br>wR2 = 0.0845	R1 = 0.0222<br>wR2 =0.0546	R1 = 0.0224<br>wR2 = 0.0649	R1 = 0.0203<br>wR2 = 0.0547	R1 = 0.0351<br>wR2 = 0.1128
Largest diff. peak/hole /e Å^{-3}	0.28/-0.35	0.22/-0.24	0.16/-0.19	0.26/-0.14	0.30/-0.21

Table S23 Refinement parameters of chosen model compounds after HAR refinement when weights were not take into account. 0.8 Å resolution.

Identification code	α-glycine	β-glycine	L-alanine	xylitol	naphthalene
Goodness-of-fit on F^2	1.1088	2.4838	1.1310	0.8079	1.0436
Final R indexes [I ≥ 2σ (I)]	R1 = 0.0160<br>wR2 = 0.0374	R1 = 0.0126<br>wR2 = 0.0293	R1 = 0.0086<br>wR2 = 0.0253	R1 = 0.0066<br>wR2 = 0.0168	R1 = 0.0079<br>wR2 = 0.0202
Final R indexes [all data]	R1 = 0.0194<br>wR2 = 0.0396	R1 = 0.0128<br>wR2 =0.0293	R1 = 0.0086<br>wR2 = 0.0253	R1 = 0.0067<br>wR2 = 0.0168	R1 = 0.0082<br>wR2 = 0.0204
Largest diff. peak/hole /e Å^{-3}	0.13/-0.14	0.08/-0.08	0.06/-0.08	0.05/-0.04	0.04/-0.06

Table S24 Refinement parameters of chosen model compounds after TAAM refinement when weights were not take into account. 0.8 Å resolution.

Identification code	α-glycine	β-glycine	L-alanine	xylitol	naphthalene
Goodness-of-fit on F^2	1.1587	2.4049	0.9895	0.8581	1.1983
Final R indexes [I ≥ 2σ (I)]	R1 = 0.0166<br>wR2 = 0.0394	R1 = 0.0122<br>wR2 = 0.0283	R1 = 0.0076<br>wR2 = 0.0221	R1 = 0. 0069<br>wR2 = 0. 0178	R1 = 0.0084<br>wR2 = 0.0232
Final R indexes [all data]	R1 = 0.0200<br>wR2 = 0.0414	R1 = 0.0124<br>wR2 =0.0284	R1 = 0.0076<br>wR2 = 0.0222	R1 = 0. 0070<br>wR2 = 0. 0179	R1 = 0.0087<br>wR2 = 0.0234
Largest diff. peak/hole /e Å^{-3}	0.12/-0.16	0.09/-0.07	0.06/-0.07	0.05/-0.05	0.04/-0.06

Table S25 wR2 obtained from IAM, HAR, TAAM models and after NoMoRe, AAM_NoMoRe refinements, when weights were not take into account during refinement.

	α-glycine	β-glycine	L-alanine	Xylitol	Naphthalene

		max resolution			
IAM	0.0888	0.0598	0.0574	0.0549	0.0915
NoMoRe	0.0723	0.0671	0.0933	0.0751	0.0952
HAR	0.0464	0.0326	0.0353	0.0294	0.0308
HAR_NoMoRe(mo)	0.0440	0.0409	0.0727	0.0503	0.0361
HAR_NoMoRe(mA)	0.0427	0.0390	0.0715	0.0486	0.0359
TAAM	0.0478	0.0309	0.0331	0.0299	0.0291
TAAM_NoMoRe(mo)	0.0557	0.0396	0.0714	0.0484	0.0359
TAAM_NoMoRe(mA)	0.0554	0.0393	0.0712	0.0483	0.0356
		0.8 Å resolution			
IAM	0.0845	0.0546	0.0649	0.0547	0.1128
NoMoRe	0.0714	0.0623	0.0901	0.0730	0.1326
HAR	0.0396	0.0293	0.0253	0.0168	0.0204
HAR_NoMoRe(mo)	0.0416	0.0385	0.0508	0.0379	0.0340
HAR_NoMoRe(mA)	0.0403	0.0363	0.0440	0.0303	0.0320
TAAM	0.0414	0.0284	0.0222	0.0179	0.0234
TAAM_NoMoRe(mo)	0.0510	0.0371	0.0459	0.0305	0.0320
TAAM_NoMoRe(mA)	0.0501	0.0363	0.0441	0.0292	0.0314

Table S26 R1 obtained from IAM, HAR, TAAM models and after NoMoRe, AAM_NoMoRe refinements, when weights were not take into account during refinement.

	α-glycine	β-glycine	L-alanine	Xylitol	Naphthalene
		max resolution			
IAM	0.0458	0.0269	0.0341	0.0298	0.0375
NoMoRe	0.0461	0.0306	0.0340	0.0398	0.0397
HAR	0.0305	0.0170	0.0137	0.0219	0.0196
HAR_NoMoRe(mo)	0.0386	0.0274	0.0237	0.0317	0.0215
HAR_NoMoRe(mA)	0.0382	0.0267	0.0233	0.0313	0.0215
TAAM	0.0294	0.0161	0.0124	0.0220	0.0191
TAAM_NoMoRe(mo)	0.0394	0.0264	0.0233	0.0310	0.0214
TAAM_NoMoRe(mA)	0.0393	0.0265	0.0232	0.0311	0.0214
		0.8 Å resolution			
IAM	0.0379	0.0222	0.0224	0.0203	0.0351
NoMoRe	0.0393	0.0259	0.0273	0.0241	0.0405
HAR	0.0215	0.0128	0.0086	0.0067	0.0082
HAR_NoMoRe(mo)	0.0354	0.0238	0.0137	0.0132	0.0108
HAR_NoMoRe(mA)	0.0348	0.0232	0.0132	0.0108	0.0108
TAAM	0.0200	0.0124	0.0076	0.0070	0.0087
TAAM_NoMoRe(mo)	0.0366	0.0226	0.0132	0.0105	0.0106
TAAM_NoMoRe(mA)	0.0363	0.0226	0.0133	0.0104	0.0107

S8.1.2. Residual maps

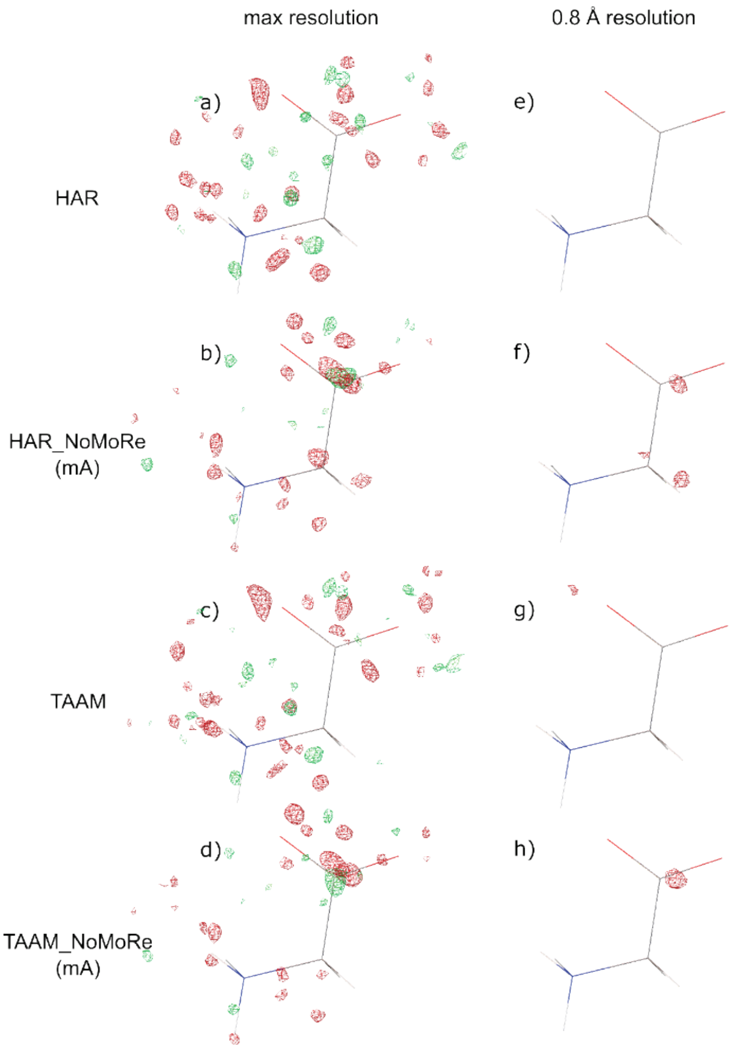

Figure S18 Residual density isosurfaces of α-glycine polymorph for max resolution and resolution cut in 0.8 Å. Maps after HAR, HAR_NoMoRe, TAAM, TAAM_NoMoRe refinement are compared. Isosurface level for 0.16 eÅ^{-1}, green positive, red negative. Weights were not take into account during refinement.

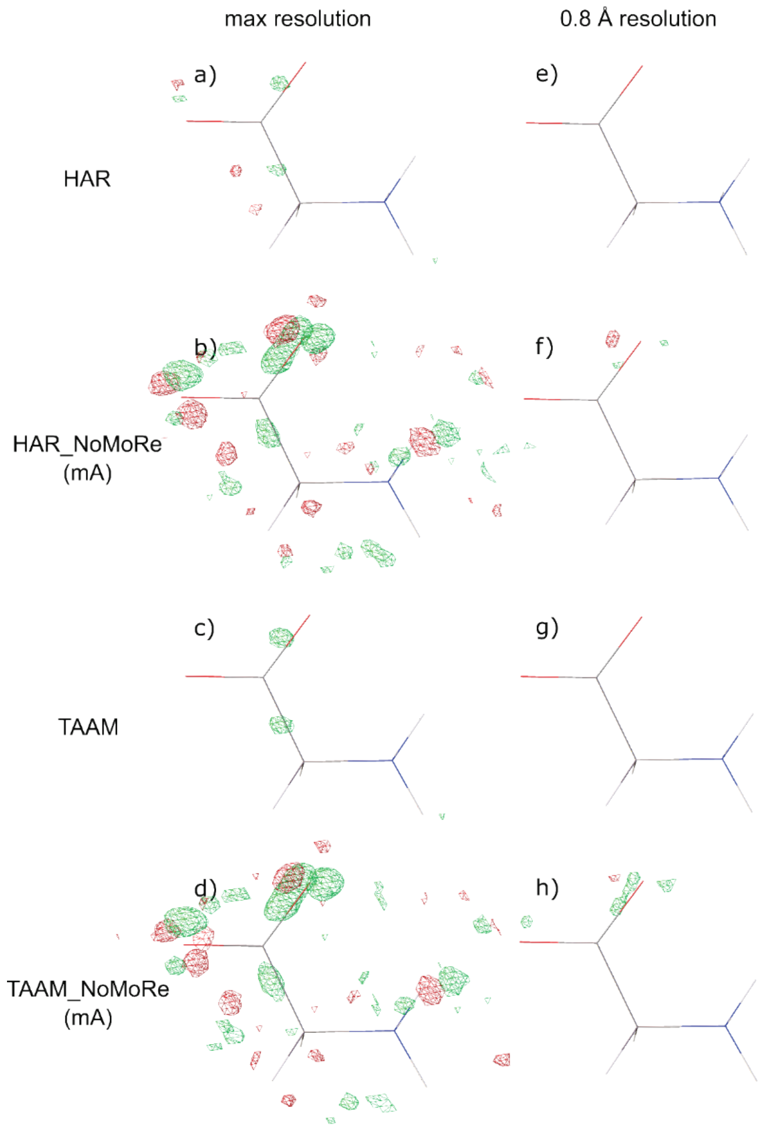

Figure S19 Residual density isosurfaces of β-glycine polymorph for max resolution and resolution cut in 0.8 Å. Maps after HAR, HAR_NoMoRe, TAAM, TAAM_NoMoRe refinement are compared. Isosurface level for 0.10 eÅ^{-1}, green positive, red negative. Weights were not take into account during refinement.

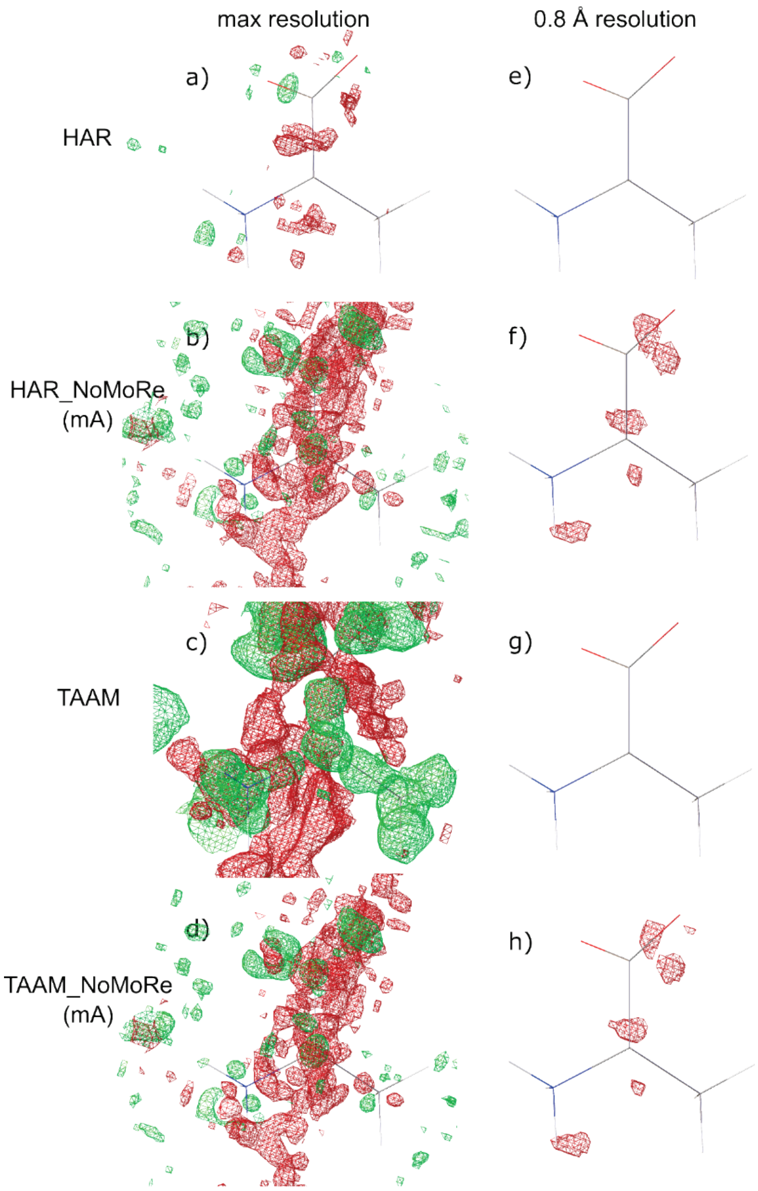

Figure S20 Residual density isosurfaces of L-alanine for max resolution and resolution cut in 0.8 Å. Maps after HAR, HAR_NoMoRe, TAAM, TAAM_NoMoRe refinement are compared. Isosurface level for 0.10 eÅ^{-1}, green positive, red negative. Weights were not take into account during refinement.

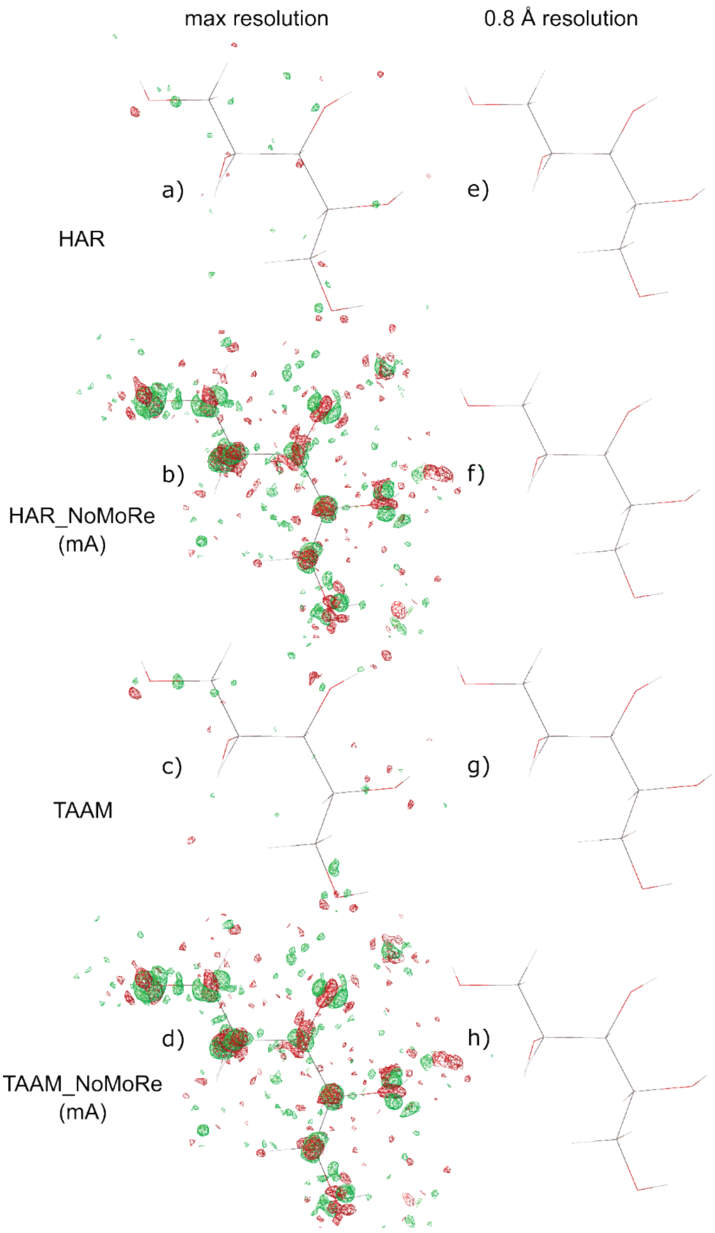

Figure S21 Residual density isosurfaces of xylitol for max resolution and resolution cut in 0.8 Å. Maps after HAR, HAR_NoMoRe, TAAM, TAAM_NoMoRe refinement are compared. Isosurface level for 0.12 eÅ^{-1}, green positive, red negative. Weights were not take into account during refinement.

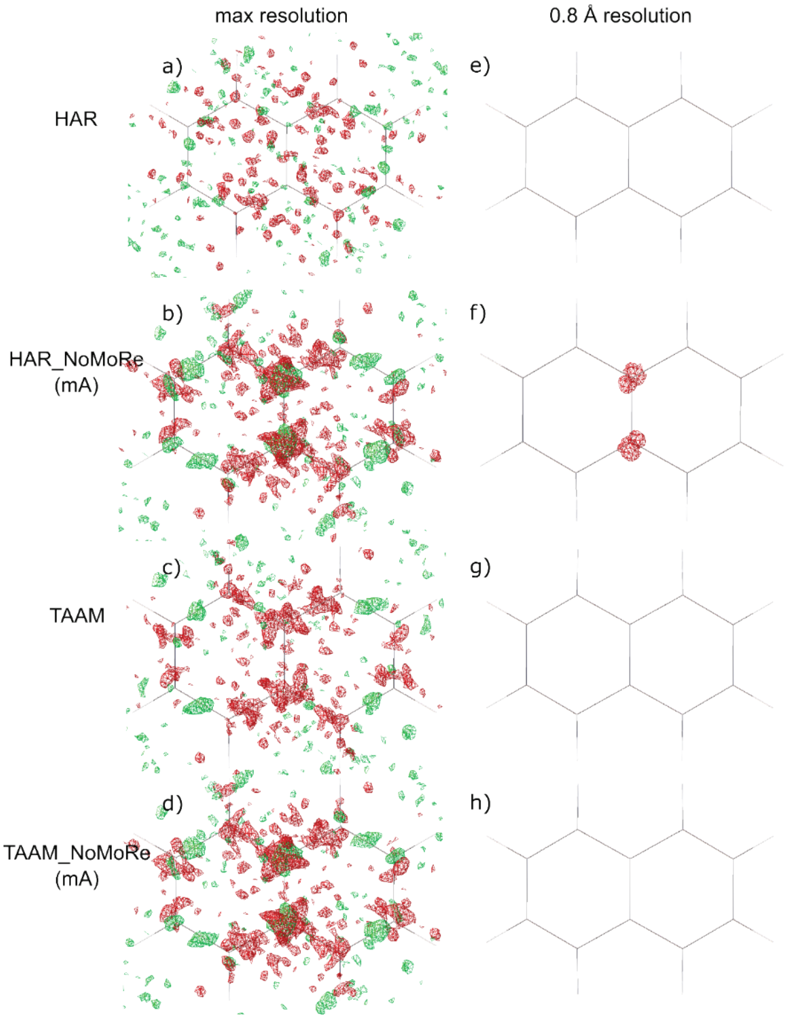

Figure S22 Residual density isosurfaces of naphthalene for max resolution and resolution cut in 0.8 Å. Maps after HAR, HAR_NoMoRe, TAAM, TAAM_NoMoRe refinement are compared. Isosurface level for 0.08 eÅ^{-1}, green positive, red negative. Weights were not take into account during refinement.

S8.1.3. Geometry and Similarity Index

Table S27 Root mean square for X-H bond lengths of structures obtained from HAR/TAAM and all tested models: HAR_NoMoRe(mo, mA) and TAAM_NoMoRe(mo, mA). Weights were not take into account during refinement. Results are expressed in Å.

	α-glycine	L-alanine	Xylitol	Naphthalene
max resolution				
HAR	0.022(9)	0.018(7)	0.017(4)	0.011(5)
HAR_NoMoRe(mo)	0.022(9)	0.018(7)	0.017(4)	0.011(5)
HAR_NoMoRe(mA)	0.026(4)	0.046(7)	0.040(5)	0.017(5)
TAAM	0.012(10)	0.037(7)	0.041(4)	0.015(5)
TAAM_NoMoRe(mo)	0.012(10)	0.037(7)	0.041(4)	0.015(5)
TAAM_NoMoRe(mA)	0.013(6)	0.040(6)	0.041(5)	0.017(5)
0.8 Å resolution				
HAR	0.028(9)	0.015(7)	0.025(7)	0.012(5)
HAR_NoMoRe(mo)	0.028(9)	0.015(7)	0.025(7)	0.012(5)
HAR_NoMoRe(mA)	0.030(4)	0.043(5)	0.049(5)	0.017(5)
TAAM	0.011(12)	0.031(7)	0.049(8)	0.017(5)
TAAM_NoMore(mo)	0.011(12)	0.031(7)	0.048(8)	0.017(5)
TAAM_NoMore(mA)	0.013(7)	0.043(6)	0.047(5)	0.018(5)

Table S28 Root mean square for H-angles of structures obtained from HAR/TAAM and all tested models: HAR_NoMoRe(mo, mA) and TAAM_NoMoRe(mo, mnH, mA). Weights were not take into account during refinement. Data are expressed in °.

	α-glycine	L-alanine	Xylitol	Naphthalene
max resolution				
HAR	1.8(6)	0.9(5)	0.5(2)	0.3(3)
HAR_NoMoRe(mo)	1.8(6)	0.9(5)	0.5(2)	0.3(3)
HAR_NoMoRe(mA)	1.9(3)	1.2(4)	0.8(3)	0.3(2)
TAAM	1.2(6)	0.5(3)	0.6(2)	0.3(3)
TAAM_NoMoRe(mo)	1.2(6)	0.5(3)	0.6(2)	0.3(3)
TAAM_NoMoRe(mA)	1.3(3)	1.1(3)	0.8(3)	0.3(2)
0.8 Å resolution				
HAR	1.8(7)	0.9(4)	0.4(3)	0.3(3)
HAR_NoMoRe(mo)	1.8(7)	0.9(4)	0.4(3)	0.3(3)
HAR_NoMoRe(mA)	1.8(3)	1.3(4)	0.8(3)	0.3(3)
TAAM	1.3(6)	0.9(4)	0.6(4)	0.3(3)
TAAM_NoMoRe(mo)	1.2(6)	0.9(4)	0.6(4)	0.2(3)
TAAM_NoMoRe(mA)	1.3(4)	1.3(4)	0.8(3)	0.4(2)

Table S29 Similarity index for non-hydrogen ($\bar{S}_{nonH}$) and hydrogen ($\bar{S}_{H}$) atoms ADPs using back-end library hikari(Tchoń & Makal, 2021). Weights were not take into account during refinement.

	α-glycine	L-alanine	Xylitol	Naphthalene
	$\bar{S}_{nonH}/\bar{S}_{H}$	$\bar{S}_{nonH}/\bar{S}_{H}$	$\bar{S}_{nonH}/\bar{S}_{H}$	$\bar{S}_{nonH}/\bar{S}_{H}$
max resolution				
NoMoRe	1.60\2.41	0.26\0.25	0.27\0.37	
HAR	0.17\25.50	0.18\2.56	0.14\1.91	0.44\0.31
HAR_NoMoRe(mo)	0.13\0.07	0.28\0.26	0.28\0.36	0.42\0.23
HAR_NoMoRe(mA)	0.12\0.07	0.26\0.25	0.29\0.37	0.42\0.23
TAAM	0.13\3.96	0.18\3.15	0.16\4.45	0.43\0.90
TAAM_NoMoRe(mo)	0.12\0.07	0.28\0.26	0.29\0.37	0.42\0.23
TAAM_NoMoRe(mA)	0.12\0.07	0.27\0.25	0.29\0.37	0.42\0.22
NoMoRe	1.61\2.38	0.21\0.22	0.30\0.44	
HAR	0.30\26.68	0.24\2.88	0.16\1.63	0.44\0.46
HAR_NoMoRe(mo)	0.14\0.07	0.26\0.27	0.36\0.36	0.40\0.24
HAR_NoMoRe(mA)	0.14\0.07	0.26\0.27	0.35\0.35	0.39\0.24
TAAM	0.23\4.40	0.20\2.27	0.22\5.71	0.46\1.03
TAAM_NoMoRe(mo)	0.13\0.08	0.28\0.28	0.35\0.37	0.38\0.30
TAAM_NoMoRe(mA)	0.11\0.07	0.29\0.28	0.36\0.37	0.38\0.30

S8.1.4. Refined frequencies for given normal modes, when weights were not take into account during refinement

Table S30 Refined frequencies for different models of α-glycine. Modes that were not refined are greyed out. Weights were not take into account during refinement.

	HAR_NoMoRe		TAAM_NoMoRe	
	mo	mA	mo	mA
max resolution				
**1**	60.758(70)	56.327(51)	51.504(73)	55.886(51)
**2**	67.729(93)	62.498(93)	57.282(86)	60.370(71)
**3**	103.005(229)	99.657(229)	97.180(316)	107.828(433)
**4**	65.051(47)	70.931(84)	67.465(88)	61.082(91)
**5**	69.053(71)	75.277(51)	75.787(91)	77.398(97)
**6**	114.675(166)	112.132(454)	133.562(167)	83.407(233)
**7**	95.306(147)	79.832(68)	88.186(105)	89.745(70)
**8**	84.950	84.950	84.950	84.950
**9**	51.694(24)	55.498(30)	66.014(64)	59.621(102)
**10**	116.554(118)	164.925(191)	113.467(178)	188.117(216)
**11**	123.510	123.510	123.510	123.510
**12**	127.850	127.850	127.850	127.850
**13**	67.234(17)	65.401(19)	58.315(16)	61.409(21)
0.8 Å resolution				
**1**	62.898(140)	62.506(105)	54.961(50)	50.567(68)

**2**	61.492(78)	59.751(65)	58.461(50)	56.698(69)
**3**	101.290(540)	97.850(226)	98.414(426)	108.612(345)
**4**	72.013(199)	79.477(94)	62.875(174)	69.215(56)
**5**	74.221(127)	68.181(62)	75.475(63)	78.497(75)
**6**	93.449(201)	114.990(404)	94.691(372)	109.953(154)
**7**	80.820(72)	74.938(56)	85.694(120)	72.042(152)
**8**	84.950	84.950	84.950	84.950
**9**	52.377(25)	54.510(32)	64.669(47)	69.363(43)
**10**	147.419(244)	151.609(131)	144.235(138)	176.143(242)
**11**	123.510	123.510	123.510	123.510
**12**	127.850	127.850	127.850	127.850
**13**	78.549(33)	76.127(41)	59.446(16)	57.769(18)

Table S31 Refined frequencies for different models of β-glycine. Weights were not take into account during refinement.

	HAR_NoMoRe		TAAM_NoMoRe	
	mo	mA	mo	mA
		max resolution		
**1**	63.579(14)	63.338(10)	64.401(12)	59.778(13)
**2**	46.100(3)	46.280(3)	46.451(3)	46.329(3)
**3**	134.150(117)	127.873(92)	147.434(132)	107.234(57)
**4**	84.819(30)	84.886(18)	84.562(20)	101.479(49)
**5**	65.917(8)	66.566(7)	65.371(7)	71.783(11)
		0.8 Å resolution		
**1**	64.211(17)	61.496(14)	66.955(14)	64.525(15)
**2**	46.321(3)	46.508(4)	47.041(4)	47.234(4)
**3**	133.819(148)	111.809(93)	139.726(235)	128.764(108)
**4**	81.592(29)	89.232(34)	79.309(17)	83.922(25)
**5**	66.097(11)	69.290(12)	66.307(13)	67.209(9)

Table S32 Refined frequencies for different models of L-alanine. Modes that were not refined are greyed out. Weights were not take into account during refinement.

	HAR_NoMoRe		TAAM_NoMoRe	
	mo	mA	mo	mA
		max resolution		
**1**	40.464(1)	40.079(1)	40,358(1)	40,414(1)
**2**	45.620(5)	46.298(5)	49,618(7)	46,679(5)
**3**	76.961(44)	78.548(59)	86,158(57)	76,441(37)
**4**	44.140(5)	41.791(5)	41,243(4)	42,384(4)
**5**	58.115(9)	55.532(7)	54,854(7)	57,049(8)
**6**	124.900(71)	117.994(85)	103,116(33)	118,530(59)
**7**	96.050	96.050	96,050	96,050
**8**	77.463(5)	78.317(5)	74,895(4)	76,388(4)

**9**	77.746(7)	88.396(10)	90,513(9)	82,794(9)
**10**	105.550	105.550	105,550	105,550
**11**	108.590	108.590	108,590	108,590
**12**	112.320	112.320	112,320	112,320
**13**	73.180(5)	85.086(7)	71,757(6)	77,638(7)
0.8 Å resolution				
**1**	37.008(4)	37.758(4)	37.867(4)	37.910(4)
**2**	42.687(12)	41.290(16)	39.474(11)	44.123(19)
**3**	64.627(74)	60.692(43)	60.833(81)	68.250(93)
**4**	42.394(17)	44.203(19)	45.776(12)	40.988(19)
**5**	57.662(34)	60.563(33)	63.187(19)	53.677(31)
**6**	108.930(156)	120.984(70)	131.870(145)	211.605(351)
**7**	96.050	96.050	96.050	96.050
**8**	78.334(11)	76.051(13)	75.668(12)	77.464(14)
**9**	89.144(28)	80.954(21)	76.370(27)	78.468(20)
**10**	105.550	105.550	105.550	105.550
**11**	108.590	108.590	108.590	108.590
**12**	112.320	112.320	112.320	112.320
**13**	65.606(12)	67.580(14)	64.600(11)	69.487(15)

Table S33 Refined frequencies for different models of xylitol. Weights were not take into account during refinement.

	HAR_NoMoRe		TAAM_NoMoRe	
	mo	mA	mo	mA
max resolution				
**1**	33.014(2)	33.152(3)	33.375(3)	31.825(2)
**2**	28.140(2)	27.710(2)	27.598(2)	27.720(2)
**3**	59.402(33)	55.899(26)	62.262(35)	60.625(25)
**4**	47.709(9)	50.268(10)	50.896(10)	49.898(12)
**5**	40.900(8)	42.046(7)	41.758(7)	39.579(6)
**6**	67.878(16)	66.056(18)	63.479(15)	81.187(29)
**7**	67.760(18)	74.605(25)	65.046(21)	65.015(22)
**8**	60.026(10)	57.038(8)	56.579(9)	65.240(13)
0.8 Å resolution				
**1**	34.651(9)	35.242(8)	34.465(7)	34.711(8)
**2**	29.504(5)	29.642(6)	29.379(5)	29.412(5)
**3**	56.476(68)	57.876(69)	54.327(52)	71.351(95)
**4**	49.555(26)	49.104(27)	48.759(26)	49.241(26)
**5**	46.497(24)	45.869(24)	44.265(21)	43.147(18)
**6**	64.930(48)	63.753(40)	64.903(39)	64.564(46)
**7**	65.279(42)	66.681(51)	70.019(52)	59.922(49)
**8**	88.458(58)	85.075(57)	96.000(62)	79.450(70)

Table S34 Refined frequencies for different models of naphthalene. Weights were not take into account during refinement.

	HAR_NoMoRe		TAAM_NoMoRe	
	mo	mA	mo	mA
		max resolution		
**1**	45.235(17)	42.352(7)	44.354(170)	44.372(11)
**2**	41.136(3)	41.487(3)	41.371(2)	41.190(3)
**3**	36.939(3)	37.262(2)	37.130(2)	37.019(3)
**4**	57.682(7)	56.414(8)	56.796(6)	57.061(6)
**5**	51.211(7)	50.479(4)	50.649(4)	50.799(6)
**6**	49.772(4)	50.467(5)	50.189(3)	50.116(5)
**7**	58.047(22)	66.238(17)	59.975(286)	59.884(17)
**8**	65.757(3)	65.172(6)	65.978(6)	65.389(6)
**9**	95.330(13)	97.342(18)	94.266(13)	95.797(14)
**10**	106.597(28)	100.830(19)	103.883(15)	105.495(22)
**11**	98.420(6)	102.091(9)	101.293(13)	97.757(8)
**12**	132.051(11)	124.441(15)	123.167(17)	133.482(13)
		0.8 Å resolution		
**1**	45.845(60)	42.009(18)	44.497(237)	47.703(26)
**2**	41.157(6)	41.204(7)	43.690(7)	42.766(8)
**3**	35.654(3)	35.759(5)	37.300(5)	36.898(4)
**4**	56.108(18)	56.886(15)	56.226(22)	54.623(26)
**5**	48.561(10)	48.419(13)	47.101(10)	48.469(11)
**6**	48.686(9)	48.139(9)	48.460(11)	49.466(14)
**7**	53.982(66)	62.942(41)	59.870(395)	54.519(26)
**8**	65.093(15)	67.098(13)	65.315(10)	64.263(15)
**9**	99.279(40)	95.012(31)	104.032(32)	104.277(57)
**10**	149.925(136)	140.930(61)	112.200(51)	126.885(74)
**11**	114.675(33)	97.395(23)	112.384(36)	113.444(36)
**12**	111.259(34)	142.785(47)	114.583(35)	114.357(48)

S8.2. Refinement results without refinement of extinction

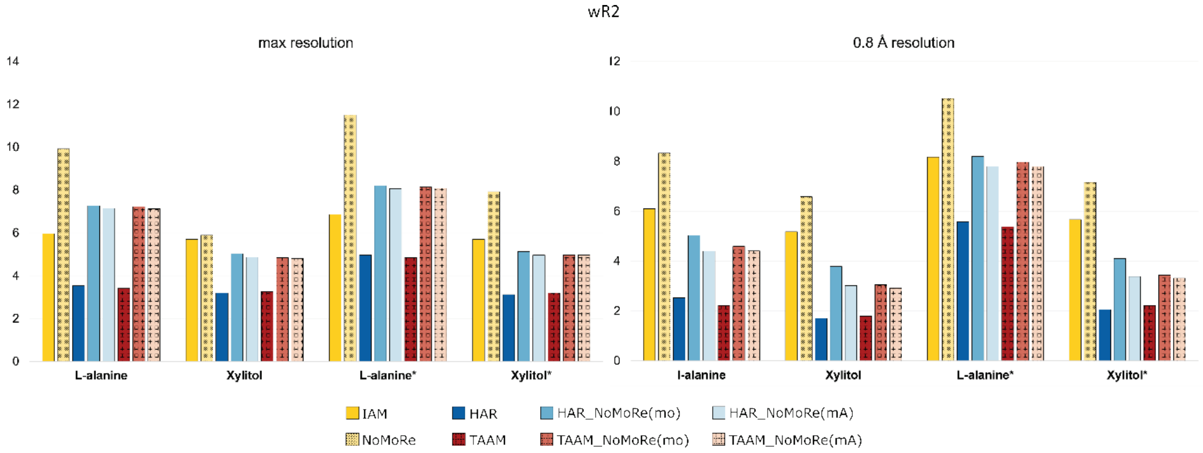

Figure S23 Comparison of wR2 obtained from IAM, HAR and TAAM models and after NoMoRe and HAR/TAAM_NoMoRe refinements for L-alanine and xylitol. Data that do not include extinction during refinement are marked with an asterisk. Left - data for max resolution, right - cutoff data in 0.8 Å resolution.

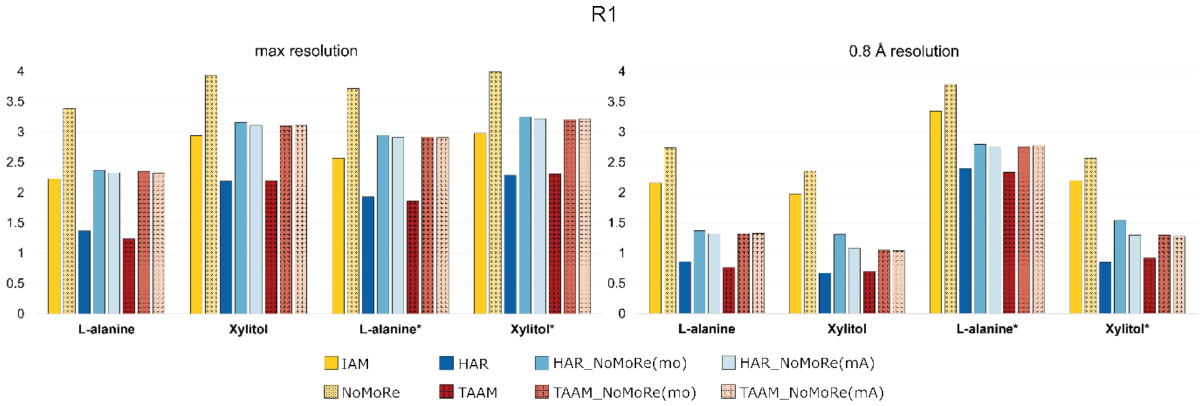

Figure S24 Comparison of R1 obtained from IAM, HAR and TAAM models and after NoMoRe and HAR/TAAM_NoMoRe refinements for L-alanine and xylitol. Data that do not include extinction during refinement are marked with an asterisk. Left - data for max resolution, right - cutoff data in 0.8 Å resolution.

Figures S23 and S24 show data for wR2 and R1 for selected compounds at maximum resolution and 0.8 Å cut-off, with and without extinction during refinement. For L-alanine, wR2 values increased without extinction, with differences around 1.5 pp for NoMoRe and aspherical atom models, and 1 pp for normal mode refinement models. For xylitol, wR2 decreased by 0.07 pp for both AAM models but increased by 0.1 pp for AAM_NoMoRe. At 0.8 Å cut-off, all wR2 values increased, ranging from 2.07 to 3.38 pp for L-alanine and 0.32 to 0.58 pp for xylitol. Changes in R1 values align with the variations in the wR2 values. All values have been collected in tables S39 and S40.

Geometry analysis (Figures S25 and S26, Tables S41 and S42) shows max resolution refinement without EXTI slightly affects bond lengths and angles. For L-alanine and xylitol, d_{RMS} decreased by no

more than 0.008 Å, except for HAR and HAR_NoMoRe(mo) in L-alanine, where it increased by 0.004 Å. At 0.8 Å cut-off, d_{RMS} values mostly increased by no more than 0.015 Å, except for xylitol (HAR and HAR_NoMoRe(mo)), which showed no change. ARMS values increased by no more than 0.5° or remained unchanged across both data ranges.

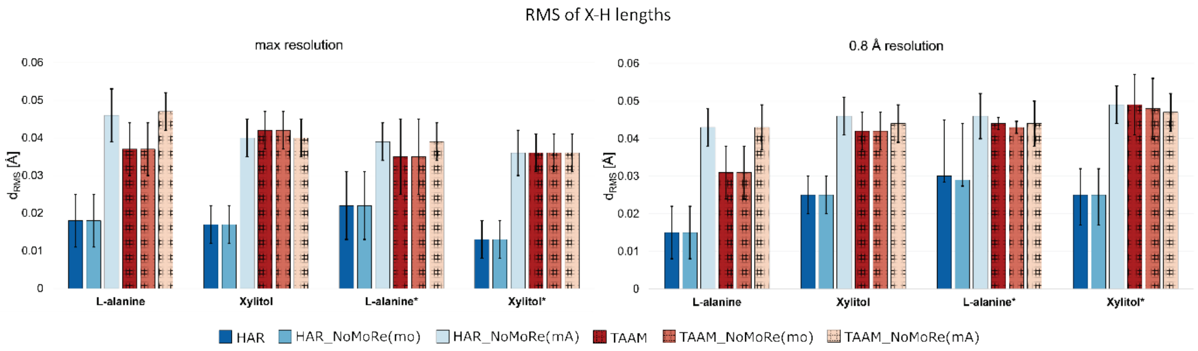

Figure S25 Comparison of root mean square for H-bonds of structures obtained from HAR/TAAM and all tested models: HAR_NoMoRe(mo, mA) and TAAM_NoMoRe(mo, mA). Data that do not include extinction are marked with an asterisk. Left – max resolution, right – 0.8 Å resolution.

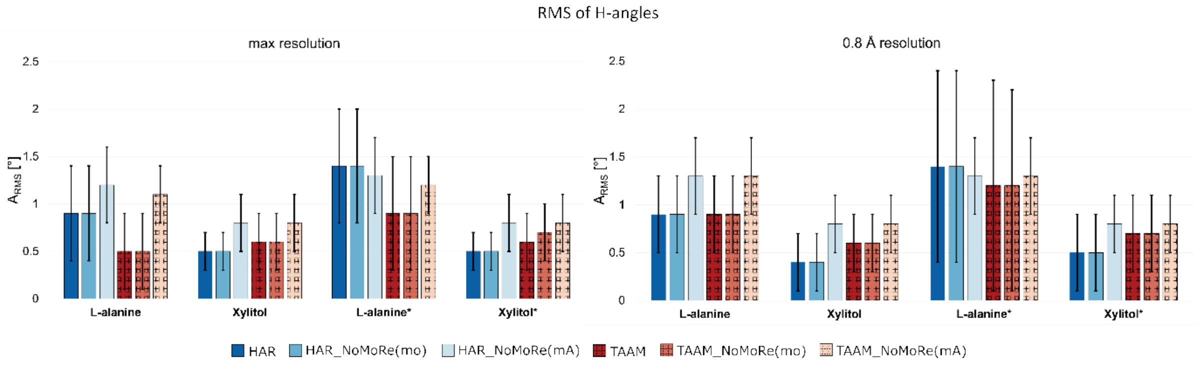

Figure S26 Comparison of root mean square for H-angles of structures obtained from HAR/TAAM and all tested models: HAR_NoMoRe(mo, mA) and TAAM_NoMoRe(mo, mA). Data that do not include extinction are marked with an asterisk. Left – max resolution, right – 0.8 Å resolution.

Figure S27 compares the similarity index for hydrogen atoms with and without considering extinction during refinement. As shown, including extinction during refinement has minimal impact on the shape of hydrogen atoms' ADPs. For numerical data see Table S43.

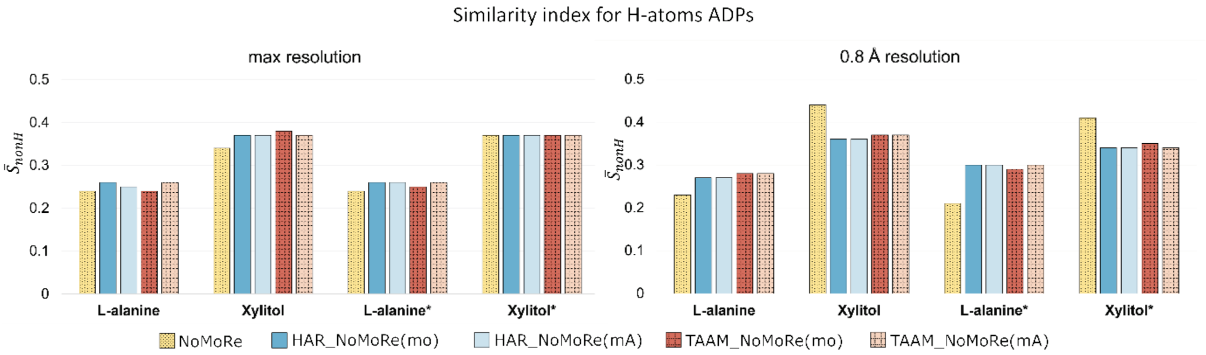

Figure S27 Comparison of similarity indexes of H-atom ADPs ($\bar{S}_H$) of L-alanine and xylitol by all tested models: NoMoRe, HAR_NoMoRe(mo, mA) and TAAM_NoMoRe(mo, mA). Data that do not include extinction are marked with an asterisk. Left – max resolution, right – 0.8 Å resolution.

These results suggest that even if the inclusion of weights (as in naphthalene) or weights and a minor contribution of extinction (as in xylitol) have little or no effect on statistics and geometries, in some cases, like in L-alanine, taking WGHT and EXTI into account during refinement results in a better-quality model.

S8.2.1. Crystallographic data and refinement parameters

Table S35 Refinement parameters of L-alanine when extinction were not take into account during refinement. Max resolution.

Identification code	IAM	HAR	TAAM
Goodness-of-fit on F^2	1.1455	1.0240	0.9965
Final R indexes [I ≥ 2σ (I)]	R1 = 0.0250<br>wR2 = 0.0679	R1 = 0.0186<br>wR2 = 0.0491	R1 = 0.0179<br>wR2 = 0.0480
Final R indexes [all data]	R1 = 0.0257<br>wR2 = 0.0686	R1 = 0.0193<br>wR2 = 0.0496	R1 = 0.0186<br>wR2 = 0.0485
Largest diff. peak/hole /e Å^{-3}	0.29/-0.54	0.28/-0.52	0.29/-0.54

Table S36 Refinement parameters of L-alanine when extinction were not take into account during refinement. 0.8 Å resolution.

Identification code	IAM	HAR	TAAM
Data/restraints/parameters	869/0/83	869/0/118	869/0/118
Goodness-of-fit on F^2	1.214	0.8698	0.8394
Final R indexes [I ≥ 2σ (I)]	R1 = 0.0334<br>wR2 = 0.0816	R1 = 0.0240<br>wR2 = 0.0558	R1 = 0.0233<br>wR2 = 0.0537
Final R indexes [all data]	R1 = 0.0335<br>wR2 = 0.0817	R1 = 0.0240<br>wR2 = 0.0558	R1 = 0.0234<br>wR2 = 0.0538
Largest diff. peak/hole /e Å^{-3}	0.28/-0.52	0.26/-0.47	0.26/-0.48

Table S37 Refinement parameters of xylitol when extinction were not take into account during refinement. Max resolution.

Identification code	IAM	HAR	TAAM
Goodness-of-fit on F^2	1.1081	0.7236	0.7413
Final R indexes [I ≥ 2σ (I)]	R1 = 0.0239<br>wR2 = 0.0557	R1 = 0.0169<br>wR2 = 0.0296	R1 = 0.0172<br>wR2 = 0.0304
Final R indexes [all data]	R1 = 0.0298<br>wR2 = 0.0570	R1 = 0.0229<br>wR2 = 0.0312	R1 = 0.0231<br>wR2 = 0.0319
Largest diff. peak/hole /e Å^{-3}	0.32/-0.26	0.20/-0.20	0.19/-0.21

Table S38 Refinement parameters of xylitol when extinction were not take into account during refinement. 0.8 Å resolution.

Identification code	IAM	HAR	TAAM
Data/restraints/parameters	1333/0/139	1333/0/199	1333/0/199
Goodness-of-fit on F^2	1.1049	0.9764	1.0042
Final R indexes [I ≥ 2σ (I)]	R1 = 0.0218<br>wR2 = 0.0566	R1 = 0.0084<br>wR2 = 0.0206	R1 = 0.0091<br>wR2 = 0.0222
Final R indexes [all data]	R1 = 0.0220<br>wR2 = 0.0567	R1 = 0.0084<br>wR2 = 0.0206	R1 = 0.0092<br>wR2 = 0.0222
Largest diff. peak/hole /e Å^{-3}	0.23/-0.19	0.08/-0.03	0.08/-0.10

Table S39 wR2 obtained from IAM, HAR, TAAM models and after NoMoRe, both AAM_NoMoRe refinements, when extinction were not take into account during refinement.

	L-alanine		Xylitol	
Resolution	max	0.8 Å	max	0.8 Å
IAM	0.0686	0.0817	0.0570	0.0567
NoMoRe	0.1151	0.1052	0.0793	0.0716
HAR	0.0496	0.0558	0.0312	0.0206
HAR_NoMoRe(mo)	0.0821	0.0820	0.0513	0.0410
HAR_NoMoRe(mA)	0.0807	0.0780	0.0496	0.0338
TAAM	0.0485	0.0538	0.0319	0.0222
TAAM_NoMoRe(mo)	0.0814	0.0797	0.0497	0.0344
TAAM_NoMoRe(mA)	0.0808	0.0779	0.0496	0.0332

Table S40 R1 obtained from IAM, HAR, TAAM models and after NoMoRe, both AAM_NoMoRe refinements, when extinction were not take into account during refinement.

	L-alanine		Xylitol	
Resolution	max	0.8 Å	max	0.8 Å
IAM	0.0257	0.0335	0.0298	0.0220
NoMoRe	0.0372	0.0379	0.0399	0.0257
HAR	0.0193	0.0240	0.0229	0.0085
HAR_NoMoRe(mo)	0.0295	0.0280	0.0325	0.0154

HAR_NoMoRe(mA)	0.0291	0.0275	0.0322	0.0130
TAAM	0.0186	0.0234	0.0231	0.0092
TAAM_NoMoRe(mo)	0.0292	0.0276	0.0321	0.0130
TAAM_NoMoRe(mA)	0.0291	0.0278	0.0322	0.0128

S8.2.2. Residual maps

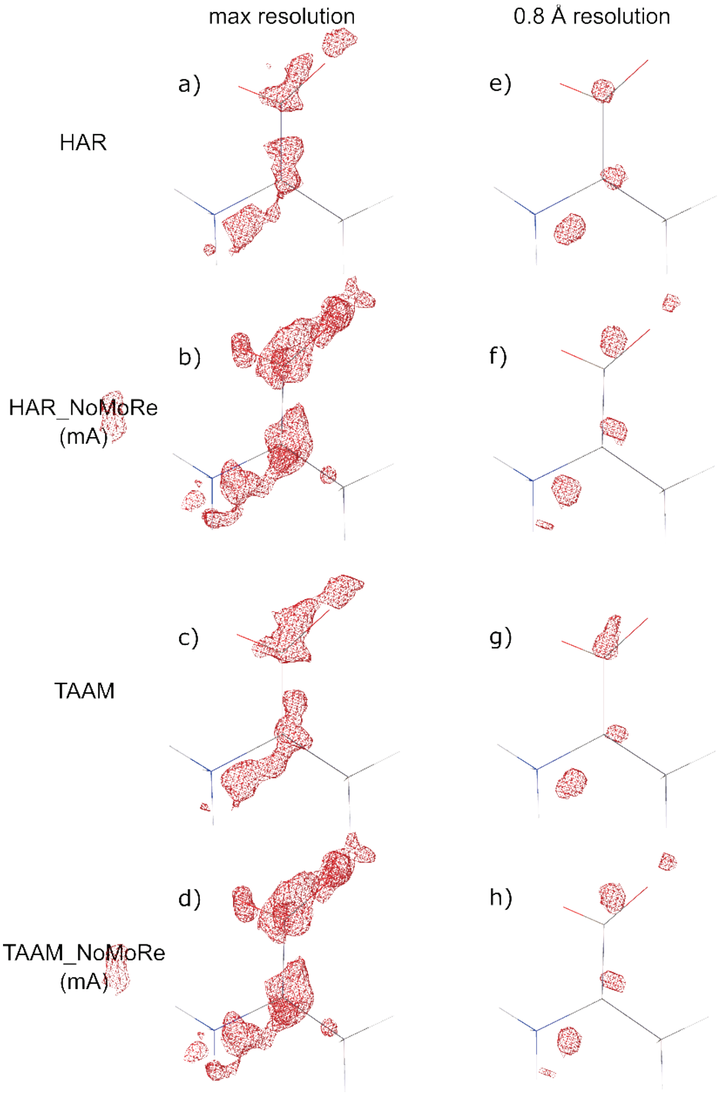

Figure S28 Residual density isosurfaces of L-alanine for max resolution and resolution cut in 0.8 Å, when extinction were not take into account during refinement. Maps after HAR, HAR_NoMoRe, TAAM, TAAM_NoMoRe refinement are compared. Isosurface level for 0.45 eÅ^{-1}, green positive, red negative.

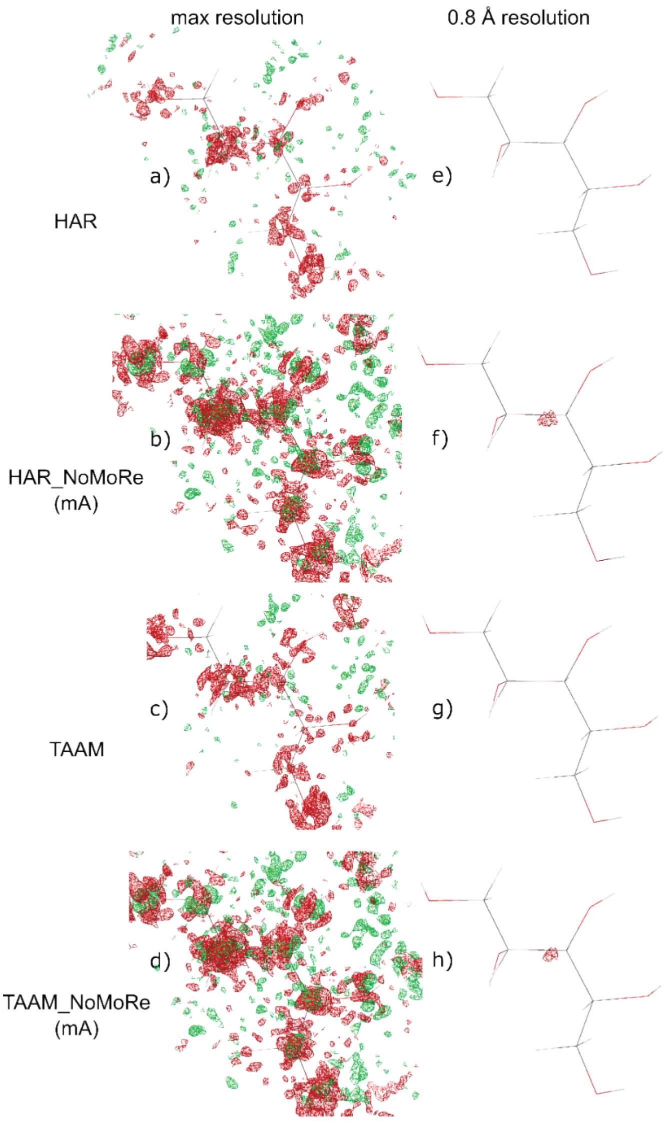

Figure S29 Residual density isosurfaces of xylitol for max resolution and resolution cut in 0.8 Å, when extinction were not take into account during refinement. Maps after HAR, HAR_NoMoRe, TAAM, TAAM_NoMoRe refinement are compared. Isosurface level for 0.12 eÅ^{-1}, green positive, red negative.

S8.2.3. Geometry and Similarity Index

Table S41 Root mean square for X-H bond lengths of structures obtained from HAR/TAAM and all tested models: HAR_NoMoRe(mo, mA) and TAAM_NoMoRe(mo, mA). Extinction were not take into account during refinement. Results are expressed in Å.

	L-alanine		Xylitol	
Resolution	max	0.8 Å	max	0.8 Å
HAR	0.022(9)	0.030(15)	0.013(5)	0.025(7)
HAR_NoMoRe(mo)	0.022(9)	0.029(15)	0.013(5)	0.025(7)
HAR_NoMoRe(mA)	0.039(5)	0.046(6)	0.036(6)	0.049(5)
TAAM	0.035(10)	0.044(16)	0.036(5)	0.049(8)
TAAM_NoMoRe(mo)	0.035(10)	0.043(16)	0.036(5)	0.048(8)
TAAM_NoMoRe(mA)	0.039(5)	0.044(6)	0.036(5)	0.047(5)

Table S42 Root mean square for H-angles of structures obtained from HAR/TAAM and all tested models: HAR_NoMoRe(mo, mA) and TAAM_NoMoRe(mo, mA). Extinction were not take into account during refinement. Results are expressed in °.

	L-alanine		Xylitol	
Resolution	max	0.8 Å	max	0.8 Å
HAR	1.4(6)	1.4(1.0)	0.5(2)	0.5(4)
HAR_NoMoRe(mo)	1.4(6)	1.4(1.0)	0.5(2)	0.5(4)
HAR_NoMoRe(mA)	1.3(4)	1.3(4)	0.8(3)	0.8(3)
TAAM	0.9(6)	1.2(1.1)	0.6(3)	0.7(4)
TAAM_NoMoRe(mo)	0.9(6)	1.2(1.1)	0.7(3)	0.7(4)
TAAM_NoMoRe(mA)	1.2(3)	1.3(4)	0.8(3)	0.8(3)

Table S43 Similarity index for non-hydrogen ($\bar{S}_{nonH}$) and hydrogen ($\bar{S}_H$) atoms ADPs using back-end library hikari(Tchoń & Makal, 2021). Extinction were not take into account during refinement.

	L-alanine $\bar{S}_{nonH}/\bar{S}_H$		Xylitol $\bar{S}_{nonH}/\bar{S}_H$	
Resolution	max	0.8 Å	max	0.8 Å
NoMoRe	0.25/0.24	0.21/0.21	0.27/0.37	0.29/0.41
HAR	0.20/5.16	0.57/33.10	0.14/2.37	0.15/2.51
HAR_NoMoRe(mo)	0.29/0.26	0.43/0.30	0.28/0.37	0.37/0.34
HAR_NoMoRe(mA)	0.29/0.26	0.44/0.30	0.29/0.37	0.36/0.34
TAAM	0.19/3.66	0.51/7.48	0.16/4.27	0.22/14.76
TAAM_NoMoRe(mo)	0.28/0.25	0.43/0.29	0.29/0.37	0.36/0.35
TAAM_NoMoRe(mA)	0.29/0.26	0.43/0.30	0.29/0.37	0.36/0.34

S8.2.4. Refined frequencies for given normal modes, when extinction was not take into account during refinement

Table S44 Refined frequencies for different models of L-alanine. Modes that were not refined are greyed out. Extinction were not take into account during refinement.

	HAR_NoMoRe		TAAM_NoMoRe	
	mo	mA	mo	mA
max resolution				
1	41,273(1)	41,153(2)	40,875(1)	41,518(2)
2	46,860(5)	48,003(5)	47,144(5)	46,764(6)
3	68,887(25)	80,244(44)	80,891(38)	85,961(58)
4	45,207(6)	42,601(4)	42,462(4)	44,083(6)
5	63,835(12)	57,184(8)	56,407(7)	59,051(9)
6	119,628(35)	131,175(67)	120,104(46)	103,658(34)
7	96,050	96,050	96,050	96,050
8	76,380(5)	77,307(5)	77,657(5)	74,525(4)
9	88,155(9)	83,407(8)	89,139(8)	79,944(8)
10	105,550	105,550	105,550	105,550
11	108,590	108,590	108,590	108,590
12	112,320	112,320	112,320	112,320
13	67,297(4)	75,930(7)	81,588(8)	74,158(6)
0.8 Å resolution				
1	44,749(6)	45,144(7)	45,080(6)	45,891(7)
2	49,878(17)	49,817(19)	55,747(26)	51,678(19)
3	108,261(184)	97,539(203)	99,875(218)	108,292(220)
4	46,500(16)	46,319(18)	43,753(16)	47,395(18)
5	63,523(27)	64,594(35)	60,301(27)	66,969(37)
6	151,860(161)	191,289(389)	183,653(426)	120,626(108)
7	96,050	96,050	96,050	96,050
8	74,839(11)	74,432(13)	76,560(12)	74,391(11)
9	74,742(16)	75,806(19)	93,449(26)	80,363(17)
10	105,550	105,550	105,550	105,550
11	108,590	108,590	108,590	108,590
12	112,320	112,320	112,320	112,320
13	67,964(13)	68,180(14)	64,963(13)	63,879(10)

Table S45 Refined frequencies for different models of xylitol. Extinction were not take into account during refinement.

	HAR_NoMoRe		TAAM_NoMoRe	
	mo	mA	mo	mA
	max resolution			
**1**	33.014(2)	33.152(3)	33.375(3)	31.825(2)
**2**	28.140(2)	27.710(2)	27.598(2)	27.720(2)
**3**	59.402(33)	55.899(26)	62.262(35)	60.625(25)
**4**	47.709(9)	50.268(10)	50.896(10)	49.898(12)
**5**	40.900(8)	42.046(7)	41.758(7)	39.579(6)
**6**	67.878(16)	66.056(18)	63.479(15)	81.187(29)
**7**	67.760(18)	74.605(25)	65.046(21)	65.015(22)
**8**	60.026(10)	57.038(8)	56.579(9)	65.240(13)
	0.8 Å resolution			
**1**	34,498(7)	35,658(9)	32,888(7)	33,204(8)
**2**	29,368(5)	29,715(5)	27,707(5)	28,054(6)
**3**	59,245(64)	63,289(75)	56,715(90)	53,295(69)
**4**	50,184(27)	48,787(24)	48,918(26)	46,963(25)
**5**	43,625(18)	42,850(20)	41,019(14)	42,544(23)
**6**	65,920(42)	61,228(38)	65,803(40)	62,972(38)
**7**	68,234(51)	66,730(42)	77,154(63)	69,867(45)
**8**	92,273(61)	91,498(59)	55,962(49)	59,909(70)

References

Tchoń, D. & Makal, A. (2021). *IUCrJ* **8**, 1006–1017.
